# Photothermal Laser Printing of Sub‐Micrometer Crystalline ZnO Structures

**DOI:** 10.1002/advs.202410771

**Published:** 2024-12-04

**Authors:** Matthias Steurer, Paul Somers, Kristian Kraft, Lukas Grünewald, Steven Kraus, Florian Feist, Bastian Weinert, Erich Müller, Stefanie Dehnen, Claus Feldmann, Yolita M. Eggeler, Christopher Barner‐Kowollik, Martin Wegener

**Affiliations:** ^1^ School of Chemistry and Physics and Centre for Materials Science Queensland University of Technology (QUT) 2 George Street Brisbane QLD 4000 Australia; ^2^ Institute of Nanotechnology (INT) Karlsruhe Institute of Technology (KIT) 76131 Karlsruhe Germany; ^3^ Laboratory for Electron Microscopy (LEM) Karlsruhe Institute of Technology (KIT) 76131 Karlsruhe Germany; ^4^ Institute of Inorganic Chemistry (AOC) Karlsruhe Institute of Technology (KIT) 76131 Karlsruhe Germany; ^5^ Institute of Applied Physics (APH) Karlsruhe Institute of Technology (KIT) 76131 Karlsruhe Germany

**Keywords:** electron backscatter diffraction, light‐to‐heat conversion, photothermal laser‐induced printing, single crystalline, transmission electron microscopy, zinc oxide

## Abstract

During light‐driven 3D additive manufacturing, an object represented in digital form is initially translated into a spatial distribution of light intensity (sequentially or in parallel), which then results in a spatial material distribution. To date, this process typically proceeds by photoexcitation of small functional molecules, leading to photochemically induced crosslinking of soft materials. Alternatively, thermal triggers can be employed, yet thermal processes are often slow and provide only low spatial localization. Nevertheless, sub‐micrometer ZnO structures for functional microelectronic devices have recently been laser‐printed. Herein, the photothermal laser‐printing of ZnO is advanced by *i*) introducing single‐crystalline rather than amorphous sub‐micrometer ZnO shapes that crystallize in the hexagonal ZnO wurtzite structure, *ii*) employing dimethyl sulfoxide (DMSO) instead of water, enabling higher local process temperatures without micro‐bubble formation, and *iii*) using substrates tailored for light absorption and heat management, resolving the challenge of light to heat conversion. Finally, the herein‐demonstrated ZnO printing requires no post‐processing and is a cleanroom‐free technique for the fabrication of crystalline semiconductors.

## Graphical abstract

The first sub‐micrometer single‐crystalline ZnO semiconductor based on photothermal laser printing of a single precursor dissolved in an organic solvent without post‐processing is introduced. Various single‐crystalline 2D geometries can be printed at low laser powers making this approach interesting for printing electronic devices. Additionally, the single‐crystalline ZnO shows non‐linear optical properties attractive for additive manufacturing of micro‐optical and micro‐piezoelectric devices.

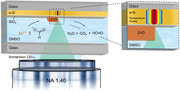

## 1 Introduction

Multi‐photon 3D laser printing of polymers has become a mature and widespread technology for the manufacturing of complex architectures on the µm‐ and nmscale, with applications covering micro‐optical components,^[^ ^1^ ^]^ micro‐robots,^[^ ^2^ ^]^ scaffolds for cell culture,^[^ ^3^ ^]^ and mechanical metamaterials.^[^ ^2^, ^4^ ^]^ In essence, a tightly focused laser beam is sent into a transparent and liquid ink and scanned in 3D along a desired path. Inside of the ink and the laser focus, the light interacts with an absorbing component, triggering a photochemical reaction that leads to the local solidification of the ink on the substrate's surface. After completing the printing step, the remaining unconsolidated still‐liquid material is simply rinsed off or dissolved.

Recently, efforts have been made to further enrich and complement (rather than replace) the materials possibilities of this technology by expanding it from organic to inorganic materials to improve the electric, mechanical, and optical properties of printed microstructures.^[^ ^5^, ^6^, ^7^, ^8^, ^9^ ^]^ One common approach to this is using an ink containing preformed nanoparticles of the target inorganic material.^[^ ^10^, ^11^ ^]^ The majority of these efforts, however, require post‐processing such as sintering of the printed structures at temperatures frequently exceeding 1000 °C.^[^ ^12^ ^]^ While the necessary temperatures have lately decreased significantly,^[^ ^13^ ^]^ this post‐processing method still complicates the development of heterogeneous multi‐material architectures, as it makes the integration of materials with different responses to heat treatments challenging. For example, one initially prints a polymer part using an organic ink. The polymer subsequently undergoes modification and transforms into a carbon‐based backbone due to subsequent high‐temperature process steps, thereby losing its original polymer properties.^[^ ^14^ ^]^ Likewise, inorganic multi‐material architectures with different post‐processing conditions and temperatures for the ingredient materials appear difficult to realize and their production remains time‐consuming.

Semiconducting ZnO deposited by laser‐induced hydrothermal synthesis with sub‐µm feature sizes has recently allowed for the direct laser printing of functional microelectronics devices and circuits in a facile process based on a transparent aqueous ink (with no nanoparticles).^[^ ^15^ ^]^ Importantly, the printed structures did not require any post‐processing other than a washing step to rinse off the unused ink. A 532 nm continuous‐wave (CW) laser at power levels of merely a few mW was sufficient to print.^[^ ^15^ ^]^ Prior to printing the ZnO, metals such as Pt and Ag were printed via a different approach, namely by two‐photon reduction of metal salts in an aqueous solution.^[^ ^15^ ^]^ While neither of these materials was printed in a truly 3D manner (containing overhanging parts), 3D layer‐by‐layer heterostructures were realized, for example, vertical electrical diodes.^[^ ^15^ ^]^ We emphasize again that these process steps are completely compatible with established multi‐photon 3D polymer laser printing.

Herein, we investigate an alternative approach to photothermal sub‐µm laser printing of ZnO, featuring four key aspects to serve as a cleanroom‐free technique for processing fully laser‐printed circuits. First – and perhaps most importantly – we demonstrate the deposition of sub‐µm thin wires that crystallize in the hexagonal ZnO wurtzite structure as single crystals, rather than amorphous or polycrystalline material. This is locally evaluated and confirmed for various printed ZnO shapes using analytical electron microscopy and diffraction techniques. The lack of centrosymmetry of these hexagonal ZnO unit cells^[^ ^16^ ^]^ opens the door for secondharmonic generation, to the linear electro‐optic effect and to printed piezoelectric elements. To avoid misunderstandings, we note that ZnO crystals of various shapes have been generated by numerous means in the literature.^[^ ^17^, ^18^, ^19^, ^20^, ^21^, ^22^, ^23^, ^24^, ^25^ ^]^ However, our simple additive approach allows for the controlled positioning of ZnO crystals at targeted locations and with sub‐µm wire widths – completely without the need for lithographic processes in a cleanroom and post‐processing heat treatments. Second, by moving from a water‐based ZnO ink^[^ ^15^ ^]^ to one based on DMSO, the boiling point increases from 100 to 189 °C, enabling higher local laser‐induced temperatures, and thereby a larger process window without bubble formation. Third, any photothermal approach necessarily requires a light‐absorbing material. Previously,^[^ ^15^ ^]^ this meant that we could not print an isolated ZnO wire on the substrate, which was a transparent glass. Here, we introduce substrates tailored for photothermal printing in terms of light absorption and – at the same time – for heat management. The latter is critical for the printing of sub‐µm structures. Fourth, we print in the so‐called sandwich mode rather than through the substrate,^[^ ^15^ ^]^ potentially enabling the facile laser printing of multi‐material architectures in a single light‐focusing mode, e.g., inside of a microfluidic cell^[^ ^26^ ^]^ suitable for laser printing under conditions of high optical numerical aperture (NA).

## 2 Results and Discussion

### 2.1 Photothermal Laser‐Induced Printing of ZnO

Photothermal laser‐induced printing has been used to fabricate metals and semiconductors by converting light from an incident CW laser into a tight temperature maximum, leading to a chemical reaction if the peak temperature is sufficiently large. This printing process is desirable as it removes the need of bulky, expensive fs‐pulsed lasers. In one example of photothermal printing, Zarzar et al.^[^ ^27^ ^]^ utilized a 750 nm CW laser to heat previously printed Pt wires by photoreduction with a fs‐pulsed laser and deposit transition metal oxides as well as transition metals. We recently showed the potential of photothermal laser‐induced printing of amorphous ZnO using Pt wires as an absorber for a 532 nm CW laser to fabricate electronic devices such as diodes and memristors.^[^ ^15^ ^]^ In both cases, the structuring of the photothermally printed material was limited to the locations and dimensions of the preprinted metal absorber.

**Figure 1:**
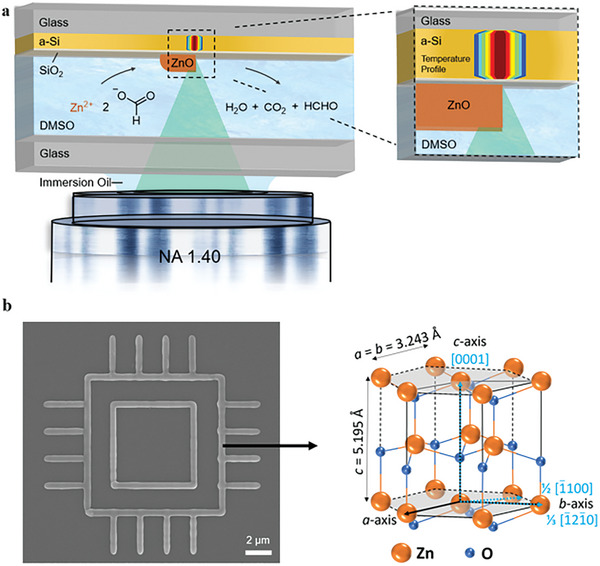
a) Scheme of photothermally laser printing ZnO. A CW laser at 532 nm wavelength is tightly focused on the surface of a Si/SiO_2_ coated glass substrate using a microscope objective lens (NA = 1.4). The DMSO‐based (Zn(HCO_2_)_2_) ink is contained between a microscope cover glass and the substrate in the sandwich printing mode. Upon absorption of the laser light by the amorphous Si layer (a‐Si, 265 nm) a thermal reaction of (Zn(HCO_2_)_2_) to ZnO occurs. A thin layer of SiO_2_ (18 nm) provides an electrically insulating layer on the substrate. b) Scanning electron microscope (SEM) of example single crystal laser‐printed ZnO with sub‐µm linewidth. Schematic representation of hexagonal wurtzite ZnO lattice (Zn orange, O blue). The bold, black lines indicate a unit cell. The gray area represents the base plane. Atomic radii are not to scale.

Our herein‐developed approach (**Figure** **1**a) makes use of a 532 nm CW laser tightly focused on the surface of a Si/SiO_2_ coated borosilicate glass coverslip (22×22×0.17 mm) which is inserted in the printing system in the sandwich mode. In the sandwich mode, the ink is located between a glass window and the substrate with the laser light passing through the ink to reach the substrate. We chose this orientation to enable future potential laser printing of multi‐material architectures in a single light‐focusing mode. The thin amorphous Si layer acting as an absorber for the 532 nm CW laser converts the laser light to heat, with a steep temperature gradient between the laser focal point and the surroundings. At sufficiently high laser powers, this results in spatially confined thermally driven reactions, e.g., the decomposition or oxidation on the ink/substrate interface. To provide and maintain the steep temperature gradient, the absorbing layer is chosen to feature a low thermal conductivity. Thus, we chose amorphous Si as the absorber for the 532 nm wavelength light. At the same time, the 532 nm laser radiation is not absorbed by the ink, ensuring that there is no photochemical process happening (see Section S1, Supporting Information). A thin layer of SiO_2_ (18 nm measured by transmission electron microscopy (TEM)) is sputtered on top of the Si resulting in an insulating surface for the fabrication of microelectronic devices. By moving the sample relative to the incident laser beam, any 2D structure, e.g., wires or circles can be fabricated. Here, we print sub‐µm singlecrystalline ZnO wires (refer to the example in Figure 1b) with a linewidth of close to 0.5 µm using laser powers around 2 mW (2.25 MW cm^−2^) and a laser scanning speed of 1 µm s^−1^ by the decomposition of zinc formate (Zn(HCO_2_)_2_) in DMSO. We chose this printing speed to facilitate the growth of single crystals which prefer a slow growth rate.^[^ ^28^ ^]^ We note that, in photopolymerization reactions, the printing speed can be significantly increased with higher light intensities as the chemical reactions continue to proceed after the energy has been deposited.^[^ ^29^ ^]^ The crystal growth process here is diffusion‐limited and therefore runs into problems at higher laser scan speeds (refer to Section S4, Supporting Information). A pulsed laser source could possibly be beneficial to better modulate the temperature profile during photothermal printing, allowing more control during the printing.^[^ ^30^, ^31^ ^]^ However, we do not explore this aspect in this work. The printing process is completed with a simple rinsing procedure using common solvents and no other postprocessing.

The choice of substrate is non‐trivial. Along with Si/SiO_2_ coated glass coverslips, we investigated the printing of ZnO directly on a commercially available Si wafer. We observed no ZnO printed on the Si wafer up to the max printing laser power tested (few 10s of mW). We attribute this observation primarily to two aspects. First, the absorption is lower and the thermal conductivity is higher in crystalline Si (wafer) than in amorphous Si (sputtered layer).^[^ ^32^ ^]^ Second, relative to the size of the laser focus region, the wafer provides an entire half‐space that can conduct heat away whereas the thin film conducts heat primarily in a 2D plane as the underneath glass coverslip acts strongly as an insulating layer. When the generated heat is rapidly conducted away from the laser focal point, a sufficiently large temperature is not reached to initiate the chemical reaction (refer to Section S2, Supporting Information). Substrates with Si thicknesses up to 265 nm were successfully fabricated, while cracking of the Si layer was observed for thicker coatings in our system. The 265 nm (determined by TEM) Si layer was used moving forward as it required the least laser power to print, attributed to the thicker layer providing more material to absorb more of the laser. In all cases, as the laser power increases, more heat is generated and the dimensions of the structures increase from ≈0.5 µm to several µm (refer to the printing parameter sweep Sections S3 and S4, Figure S5, Supporting Information). Thus, the dimensions of the intended structures can be tuned regarding the laser power to enable repeatable printing of small uninterrupted sub‐µm linewidth single‐crystalline ZnO structures (Figure 1b).

Photothermal laser‐induced printing previously used a solution of a metal precursor and a reducing or oxidizing agent in water,^[^ ^7^, ^9^ ^]^ requiring changing of the pH of the ink by adding acids or bases such as hydrochloric acid or ammonia to dissolve or modify the metal precursor.^[^ ^15^ ^]^ Additionally, this contributed to an undesirable incompatibility between different inks/materials and limited the application of the ink.^[^ ^15^ ^]^ As for the solvent, water has a low boiling point of 100 °C, which often causes bubbles to form during the printing that disrupt the printing process.^[^ ^33^ ^]^ Such interference can lead to an increased feature size or interrupt the patterning completely. Here, we improved the ink formulation for printing ZnO using a saturated solution of (Zn(HCO_2_)_2_) in DMSO. Zinc formate readily decomposes to the Zn^2+^ precursor and DMSO exhibits a higher boiling point than water at 189 °C allowing a larger processing temperature before bubble formation. We propose that the temperature increase caused by the laser irradiation leads to decomposition of Zn(HCO_2_)_2_ and forms ZnO accompanied by gaseous products, observed for the thermal decomposition of Zn(HCO_2_)_2_ in the literature, of H_2_O, CO_x_^[^ ^34^, ^35^ ^]^ and HCO_2_CH_3_^[^ ^36^, ^37^ ^]^ produced by the Tishchenko reaction of HCHO in the presence of weakly basic ZnO (Figure 1a). Furthermore, the formation of ZnO by thermal decomposition of Zn(HCO_2_)_2_ is observed in an oxygen‐rich atmosphere as well as under anoxic conditions.^[^ ^38^, ^39^ ^]^ The proposed mechanism of ZnO formation follows two steps described in the literature (determined by thermogravimetry of bulk Zn(HCO_2_)_2_.^[^ ^38^, ^40^ ^]^ First, a dehydration process of Zn(HCO_2_)_2_ dihydrate at temperatures exceeding 100 °C takes place. Second, the anhydrous Zn(HCO_2_)_2_ decomposes at temperatures around 270–310 °C leading to ZnO. In the literature^[^ ^36^ ^]^ the chemical reactions describing the decomposition step are divided into two stages. In Stage I, the formate anion decomposes to an intermediate hydride anion and carbon dioxide. In Stage II, the hydride anion then reacts with the formate anion likely leading to H_2_CO_2_^2−^,^[^ ^36^, ^37^ ^]^ which spontaneously decomposes to O^2−^ and HCHO yielding ZnO. The zinc oxide formation is enhanced by the decrease of the ink viscosity during laser irradiation,^[^ ^41^, ^42^ ^]^ enabling fast diffusion of surrounding O_2_ to the heated substrate surface. In general, we observe no microbubble formation through in situ optical imaging during printing. This indicates that the formation of gaseous products is small and not an issue as well as indicating that the process temperature does not boil the DMSO, suggesting that the dehydration‐ and decomposition temperatures in DMSO are lower than observed for the decomposition of bulk Zn(HCO_2_)_2_ in the literature.^[^ ^38^, ^40^ ^]^ Finally, DMSO also has a closer refractive index to the objective lens design (i.e., that of the glass coverslip) than other highly polar solvents,^[^ ^43^ ^]^ allowing for better focusing of the laser light (i.e., a smaller focus spot, see Section S5 and Figure S6, Supporting Information) through the ink in the sandwich mode. We investigate the microstructure and chemical composition of the printed ZnO material in the following.

### 2.2 Crystallinity of Printed ZnO Objects

A detailed characterization was performed to reveal the crystalline structure of the printed ZnO objects. A workflow was developed to derive structural information at high resolution on comparably large lateral scales, succeeding our previous exploratory efforts.^[^ ^44^ ^]^ After polishing the µm‐sized ZnO structures by focused‐ion‐beam (FIB) milling, we apply electron backscatter diffraction (EBSD), a surface‐sensitive diffraction technique in the scanning electron microscope (SEM), on printed wires, circles, and angles to analyze their crystallinity on the µm‐scale.

**Figure 2:**
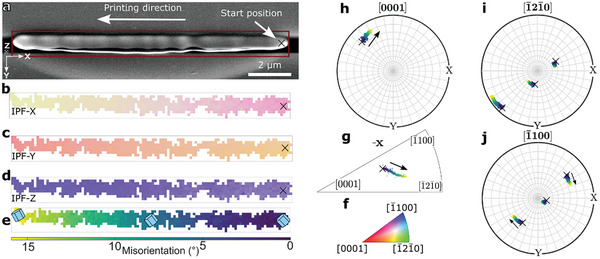
Analysis of the crystallinity of a photothermally laser‐printed 10.4 µm long ZnO wire by electron microscopy and diffraction. a) Top‐view SE‐SEM image shows the wire with an FIB polished surface necessary for conducting EBSD measurements. The outer coordinate system (*x*, *y*, *z*) was chosen as shown in (Figure 2a), where *x* and *y* are the lateral directions parallel to the surface and *z* is the surface normal. For this wire, the printing direction is ‐*x*, as indicated. b–d) Color‐coded IPF maps of the polished ZnO surface, with the corresponding color legend given in the stereographic triangle shown in (f). e) Misorientation plot of ZnO relative to the reference orientation located at the start of the printing process (marked with a cross in b–d). The total scalar misorientation is ≈16° with a misorientation rate of 1.7° µm^−1^. Schematics of the local orientation of the hexagonal lattice at three positions are overlaid. f) Color legend for IPF maps of ZnO. g) IPFs in printing direction (*‐x*). h‐j PF for the [0001], $\left[\bar{1}2\bar{1}0\right]$, and $\left[\bar{1}100\right]$ directions. The arrows in the (inverse) PFs indicate the direction of the crystal rotation.

Initially, the crystallinity and structural integrity were analyzed along the entire length of a printed ZnO wire with a nominal length of 10 µm using EBSD, as shown in **Figure** **2**. The top‐view secondary electron (SE) image reveals an actual length of 10.4 µm of the ZnO wire and shows the FIB‐polished surface (Figure 2a) needed to perform EBSD mapping within the marked rectangular area. However, the length of the polished EBSD surface is 9.4 µm. One must emphasize that EBSD analysis requires a predefined outer coordinate system to draw connections to the material's crystalline orientation. The outer coordinate system (*x*, *y*, *z*) was chosen as shown in (Figure 2a), where *x* and *y* are the lateral directions parallel to the surface and *z* is the surface normal. For this wire, the printing direction is ‐*x*. The directions *x*, *y*, and *z* have the same meaning in the inverse pole figure (IPF) maps (Figure 2b–d), within the IPF (Figure 2g), and within the pole figures (PF)s (Figure 2h–j).

The color code for the IPF maps shows the crystalline orientation (given by the stereographic triangle in Figure 2f) parallel to one of the given real‐space directions (*x*, *y*, or *z*). Only the central, FIB‐polished area of the ZnO wire yielded useful EBSD patterns for subsequent automated indexing, resulting in unindexed patterns (white pixels) toward the edges of the IPF maps (Figure 2b–d). Nanometer‐sized pores in the ZnO structure were observed (not shown here, cf. Section S6, Supporting Information), which may locally contribute to a poor EBSD pattern quality that cannot be indexed anymore. The automated indexing procedure was successful for the hexagonal ZnO wurtzite structure (*P*6_3_ *mc*, space group 186, schematically shown in Figure 1b) upon all indexed patterns in the various measured printed ZnO objects.

One single general color in each IPF map indicates that one large ZnO grain, or, in other words, a single crystal has formed, as opposed to a polycrystalline or an amorphous structure (Figure 2b–d). However, within the single crystal, a color gradient along the printing direction is observed in all three IPF maps (Figure 2b–d), indicating that the crystal orientation of the ZnO grain varies along the ZnO wire.

This aspect becomes clearer by calculating and displaying the overall scalar misorientation between all orientations in the map to a reference orientation (defining 0°) chosen at the start of the printing (Figure 2e). The misorientation values increase toward the highest measured angles at the final printing position. Note that the scalar misorientation values correspond to the minimum value of all symmetrically equivalent orientations. The total scalar misorientation was measured to be ≈16° over the entire printed wire, corresponding to an average rotation rate of 1.7° µm^−1^. For the printed ZnO wire in Figure 2, the lattice rotates continuously as the mean rotation rate is linear. In contrast, we also observed non‐constant rotation rates on other wires (refer to Section S7 and Figure S10, Supporting Information).

A similar continuous rotation of the crystal lattice has been observed, e.g., for branches of spherulitic crystals,^[^ ^45^, ^46^ ^]^ transrotational crystals,^[^ ^47^, ^48^ ^]^ or in laser‐heated glasses.^[^ ^49^, ^50^ ^]^ For other liquid inkbased printing of ZnO,^[^ ^51^, ^52^ ^]^ agglomerated nanowires were observed.

To understand the growth of the ZnO wurtzite single crystal in the direct laser writing process, we first investigate the initial heterogeneous nucleation of the crystal on the amorphous substrate. This investigation is exemplified by the printed ZnO wire shown in Figure 2 and is representative of the 24 wires analyzed (refer to Section S8 and Figures S12–S17, Supporting Information for texture analyses of all wires). In Figure 2, the orientation of the crystal in the IPF‐X map at the print starting point (marked with a cross in Figure 2b), is initially aligned in a random crystallographic direction (here around $\left[\bar{9}\;11\;\bar{2}\;10\right]$) at the start of the printing process and rotates toward the $\left[\bar{1}2\bar{1}0\right]$ direction until the end of the printed wire (Figure 2g). Alternatively, the rotation of the crystal lattice can also be observed in the PFs (Figure 2h–j). The values in the PFs are color‐coded with their misorientation values shown in Figure 2e. The PF in the $\left[\bar{1}100\right]$ direction (Figure 2j) indicates that the crystal's $\left\langle \bar{1}100\right\rangle$‐direction is initially closely aligned with the *z*‐direction. With progressing crystal growth following the laser writing direction the crystal lattice then approximately rotates clockwise around this crystal direction (marked with arrows Figure 2j), that is, an in‐plane rotation (in the *xy*‐plane) of the ($\bar{1}100$) planes parallel to the surface around the *z*‐direction. The rotation behavior of the lattice is depicted using color‐coded scalar misorientation values, as shown in Figure 2e. For a more intuitive visualization of lattice rotation, Figure 2e illustrates the corresponding rotation of a schematic hexagonal ZnO unit cell in real space at three positions along the printed wire.

Another interesting crystallographic direction is [0001], which is depicted in the PF in (Figure 2h). For the ZnO wurtzite structure, the *c*‐axis, here referred to as the [0001] direction, is known to crystallize the fastest.^[^ ^53^ ^]^ As a result, ZnO nanowires^[^ ^52^ ^]^ (and another similar example)^[^ ^51^ ^]^ tend to have their longest axis along the [0001] direction. Additionally, ZnO films exhibit lamellar growth, with the [0001] direction parallel to the surface normal.^[^ ^40^ ^]^ In our case, the [0001] direction of the crystal rotates toward the ‐*y* real‐space direction around the *z‐*direction (marked with an arrow in Figure 2h). Notably, the [0001] direction does *not* rotate toward the surface normal *z* for the shown ZnO wire, indicating that the preferred crystallographic growth direction [0001] in ZnO does not necessarily align with the surface normal (*z*‐direction) during printing. However, some investigated ZnO wires showed a tendency toward [0001]||*z*‐oriented growth along the printing direction (see Section S8 and Figure S12, Supporting Information), suggesting that [0001]||*z* growth is possible in the laser writing process depending on the initial orientation during nucleation at the starting point of printing and subsequently growth depending on the laser‐printing direction relative to lattice orientation.^[^ ^49^ ^]^ In addition, the [0001] direction also does not tend toward the printing direction *x* (Figure 2g; Section S8, Figure S15, Supporting Information for other wires), indicating that the growth front is not parallel to *x* but instead inclined relative to the printing direction due to interactions with the substrate surface. For completeness, the PF in the $\left[\bar{1}2\bar{1}0\right]$ direction is also shown (Figure 2i).

**Figure 3:**
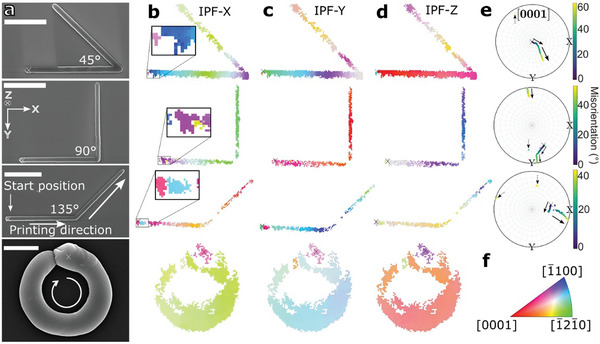
EBSD analysis of different laser‐printed ZnO shapes. a) SE‐SEM top‐view images of ZnO structures featuring 45, 90, and 135° angles, as well as a circle with a 1 µm radius, printed in a clockwise direction. Scale bars correspond to 5 µm for the angles and 1 µm for the circle. The starting positions of the printing process are marked in the SEM images, IPF maps, and PFs with a cross. The chosen outer coordinate system indicates the real‐space directions (*x*, *y*, *z*). b–d) Color‐coded IPF maps for the three angles and one circle. Small ZnO grains are at the start position in the magnified IPF‐X maps of the angles. e) PF for the [0001] direction. The arrows in the PFs indicate the direction of the crystal rotation. f) Stereographic triangle with a color legend for IPF maps (b–d).

The laser‐printed single crystalline straight wires have demonstrated, on average, that they nucleate with an unspecified initial orientation on the amorphous substrate, and the lattice subsequently undergoes continuous rotation during growth. To further investigate the influence of the printing direction, we examined different printed ZnO structures, including *i*) shapes with varying angles and *ii*) circles with different diameters, as shown in **Figure** **3**; Section S7, Figures S9 and S11 (Supporting Information). This also confirms that our approach can print shapes other than straight wires.

As previously, the starting point of the printing process is denoted by a cross. Initially, for all angles, printing commences with the horizontal part along the real‐space *x‐*direction. Subsequently, it progresses toward predefined angles (45, 90, and 135°), with the printing direction marked for the 135° angle in Figure 3a. For the circle, the printing starts at the 12o'clock position and continues clockwise. The radius of the shown circle is 1 µm, but other radii were investigated as well (refer to Section S7 and Figure S11, Supporting Information). Similar to Figure 2, all IPF maps show smooth color gradients suggesting primarily a single large ZnO grain with a rotating lattice and an independent primary starting orientation (Figure 3b–d, IPF map legend given in Figure 3f). However, closer inspection of the IPF maps reveals a few extra smaller grains located at the starting point of the printing (refer to the enlarged inset in Figure 3b–d). The polycrystalline structure observed at the starting point may be attributed to the initial ZnO nucleation on the amorphous substrate. The absence of structural guidance from the substrate results in random nucleation and growth of ZnO grains,^[^ ^47^ ^]^ possibly caused by the initial temperature: if the temperature is higher in the initial phase of printing, the formation of smaller grains and a higher nucleation density are more prominent. Multiple nucleated ZnO grains with different orientations grow at the starting position of the laser, which are visible as dots in the PF (refer to the dashed arrows in Figure 3e) before only one of these initial orientations, i.e., a single, primary orientation, proceeds to grow and rotate during the printing process. The resulting orientation is denoted as “primary orientation” in the following.

The short wire in Figure 2 shows only one primary orientation without extra grains in the beginning, indicating that the extra grains at the starting point do not form every time or even slight differences in printing parameters change the local temperature conditions hence trigger different solid nucleation mechanisms. Comparing the different printed shapes, we observed that the primary orientation of the crystal differs in each case (Figure 3b–d). This suggests that the initial orientation of the ZnO seed forming the primary ZnO orientation along the printed structure is likely random. Additional measurements on other ZnO structures shown in the Supporting Information support this observation (Section S8, Supporting Information) as the starting orientation of the primary orientation is always different.

The total scalar misorientations for the 45, 90, 135° angles and the 1 µm‐circle are 60.54, 52.37, 49.25, and 3.63°, respectively. The total lengths of the angles or circumference of the circle are 21.46, 21.02, 20.08, and 4.41 µm, which corresponds to average rotations rates of 2.82, 2.49, 2.45, and 0.83° µm^−1^ for 45, 90, 135° angles and the 1 µm‐circle, respectively. Note, that the reference positions for the misorientation calculations are chosen from the start of the primary orientation rather than a random grain at the (possibly) polycrystalline starting point of the printing. The total scalar misorientation and rotation rates for the shown angles are higher than for the short ZnO wire (1.7° µm^−1^, Figure 2). However, no clear trend in differences in average rotation rates between straight wires, angles, and circles was observed within experimental errors and statistics of our measured samples. The rotation rates for the additionally measured 21 wires in the Supporting Information (Figure S12, Supporting Information) vary from 0.19 to 6.66° µm^−1^. The average rotation rates for angles from Figure 3 and Figure S9 (Supporting Information) are between 0.73 and 2.99° µm^−1^ and those of the circles from Figure 3 and Figure S11 (Supporting Information) are between 0.83 and 1.81° µm^−1^. A statistical *t*‐test for independent sampling was used to check whether the differences in the mean values of the rotation rates of the wires (3.13 ± 1.56)° µm^−1^, angles (2.26 ± 1.04)° µm^−1^ and circles (1.30 ± 0.49)° µm^−1^ are statistically significant. The errors indicate the standard deviations for *N*  =  21, *N*  =  4, and *N*  =  3 for wires, angles, and circles, respectively. A significance level of 0.05 and a homogeneous variance were assumed. The two‐tailed *p*‐value for the comparison between wires and circles is 0.06, for wires and angles is 0.30, and for circles and angles is 0.21. Since the values are all higher than 0.05, the observed differences between the measurements are not statistically significant. The aforementioned otherwise‐grown rotating crystals with rotating lattices exhibit rotation rates up to 90° µm^−1^,^[^ ^45^, ^48^, ^50^ ^]^ while rotating crystals with lower thickness tend to rotate faster.^[^ ^40^ ^]^ Therefore, the differences in rotation rate are likely affected by the total height of the printed objects parallel to the surface normal *z*, which itself is also affected by the printing speed and laser power (refer to Section S4 and Figure S5, Supporting Information). In addition, differences in the crystal structure, preferred growth directions, and the presence of pores in our printed objects may alter the rotation rate.

Finally, the rotation rate also depends on the laser‐writing direction. However, we found no consistent changes in lattice rotation, regardless of the printing direction. This is likely because the crystalline ZnO is printed on an amorphous substrate, leading to random initial orientations. Therefore, the substrate does not predefine the crystal orientation and the printing direction does not matter. However, when printing from an already‐formed crystal seed, changes in the printing direction influence the lattice rotation direction and rotation rate, with variations depending on the current orientation relative to the printing direction, as observed for the printed angles. The average rotation rates for the horizontal part of 45, 90, and 135° angles are 4.90, 3.93, and 3.35° µm^−1^. In contrast, the average rotation rates for the diagonal part of the 45, 90, and 135° angles are 2.98, 3.13, and 4.99° µm^−1^. Thus, the rotation rate is smaller for the 45 and 90° angles after bending and slightly larger for the 135° angle. This indicates that the rotation of the ZnO crystals depends on the direction of printing. Further angles and their rotation rates are shown in Section S7 and Figure S9 (Supporting Information). Such a dependence of the crystal rotation and rotation rate on the printing direction has been also observed for rotating Sb_2_S_3_ crystals.^[^ ^49^ ^]^

Indeed, the printed angles have a clearly defined change in printing direction, whereas the straight wires display no change or the circles have a continuous change in printing direction, respectively. Therefore, the angles provide insights into the interplay between lattice rotation and printing direction. Video S1 (Supporting Information) shows background‐subtracted EBSD patterns at different positions along the angles. A significant change in the moving direction of the EBSD patterns is observed for all angles when the printing direction changes. This demonstrates that the latter influences the direction of the lattice rotation and, as a result, also the rotation rate, in agreement with literature for laser‐printed Sb_2_O_3_ in glass.^[^ ^49^, ^54^ ^]^ The directional change in EBSD‐pattern movement seems to correlate with the angle value. For example, for the 90° angle, the EBSD‐pattern movement is initially roughly horizontal at the horizontal part of the wire. After the 90° angle, the EBSD pattern moves in the vertical direction, i.e., a ≈90° rotation is observed for the patterns similar to the printed 90° angle (Video S1, Supporting Information). As a result, clear changes in the corresponding (inverse) PFs for the angles for a change in orientation are observed, and example [0001] PFs for the three shown angles are displayed in Figure 3e. Note that the exact angle between the focus‐scan direction and the orientation change in the PFs does not necessarily agree due to *i*) the stereographic projection and *ii*) the chosen PF direction (here [0001]).

Besides the representative ZnO structures in Figures 2 and 3, similar EBSD measurements were performed on more examples, and additional printing parameters are shown in the Supporting Information (refer to Sections S4, S7, and S9, Figures S4, S9, and S11, Supporting Information). For instance, to determine whether the lattice rotation reaches saturation, a straight ZnO wire with a length of 103.5 µm, ten times longer than the wire printed in Figure 2, was measured. However, the length of the polished EBSD surface is 98.6 µm. The lattice rotation rate remains constant over the entire length and exhibits a total scalar misorientation of 18.7°. This corresponds to an average rotation rate of 0.19° µm^−1^ (refer to Section S9, Supporting Information). Along with the printing direction, the printing speed and laser power are fabrication parameters that influence the quality of the ZnO crystals. Increasing laser power results in more deposition of ZnO material on the substrate, which decreases the printable resolution. Conversely, higher printing speeds lead to reduced material deposition and an increase in unindexed patterns, indicating lower‐quality ZnO crystals (refer to Section S4, Supporting Information).

We summarize the EBSD results as follows. The printed ZnO structures are single‐crystalline with a continuous lattice rotation. The rotation rate and direction were different for each investigated ZnO structure. The rotation direction is influenced by the printing direction, but other than that, no clear mechanism or preference for the rotation rate and direction can be established based on the exhibited results. The initial ZnO orientation is random on the amorphous SiO_2_ substrate. A single‐ or polycrystalline starting point may form and a primary orientation is subsequently established upon printing. For a polycrystalline seed, the primary orientation may be influenced by the spatial location of the initial grain relative to the printing direction. Specifically, the grain closest to the moving laser focus tends to preferentially grow and establish the primary orientation. Another possibility is that the favorable primary ZnO orientation is chosen based on the ability of the crystal to release stress in the crystal lattice by the formation of point defects and dislocations^[^ ^45^, ^55^, ^56^ ^]^ or alternatively a gradual change in interatomic distances with forming dislocations.^[^ ^48^ ^]^ In any case, the crystal then continues to grow with the primary orientation but also *i*) changes toward a different crystallographic orientation and *ii*) interacts with the substrate and surrounding ink, which ultimately results in the observed rotating lattice. A systematically preferred growth direction of [0001]||printing‐direction or alternatively [0001]||*z* of the ZnO wires, angles, and circles was not observed (refer to Section S8 and Figures S12, S15 and S16, Supporting Information). The growth front may not be aligned with either of these orientations due to interaction with the substrate.

Regarding preferential orientations, hexagonal ZnO typically grows fastest along the [0001] orientation as observed for laser‐grown^[^ ^52^, ^53^ ^]^ or otherwise produced, e.g., ZnO nanowires.^[^ ^53^, ^57^, ^58^ ^]^ The preferred growth direction of the ZnO nanowire is the [0001]‐axis parallel to the growth direction, which is typically the substrate surface normal (here *z*). As the sample surface‐sensitive EBSD^[^ ^59^, ^60^ ^]^ will not provide information down to the ZnO/SiO_2_ interface, complementary cross‐section TEM was performed and is discussed in the following section.

### 2.3 High‐Resolution Analytical TEM Diagnostic of the Inner Structural Arrangement of the Printed ZnO

**Figure 4:**
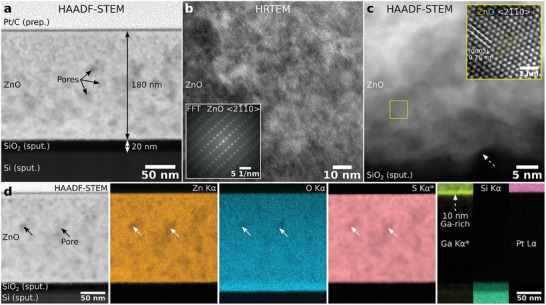
Analytical TEM analyses of the ZnO wire cross‐section conducted after the EBSD measurements shown in Figure 2.a) Cross‐section overview HAADF‐STEM image showing the porous ZnO film and the deposited SiO_2_ and Si layers. High‐resolution (S)TEM images of the ZnO film b) in the middle and c) close to the ZnO/SiO_2_ interface (cf. dashed arrow) showing a single ZnO $\left\langle 2\bar{1}\bar{1}0\right\rangle$ zone‐axis orientation. The insets show b) the FFT of the HRTEM image with overlaid simulated spot positions and c) a magnified region of the HAADF‐STEM image with the Zn atomic columns appearing as white dots. d) HAADF‐STEM signal and corresponding elemental maps. The maps marked with a star (*) were denoised with PCA. The Ga, Si, and Pt signals do not vary along the horizontal direction, so only one‐third of each map is shown for brevity. A 10 nm thin Ga‐rich surface layer is observed which is created by the FIB polishing process for the preceding EBSD measurements.

Additional cross‐section scanning transmission electron microscopy (STEM) analyses were conducted after EBSD measurements on the ZnO wire shown in Figure 2a to analyze the crystal structure and chemical composition (**Figure** **4**). High‐angle annular dark‐field (HAADF) STEM overview imaging shows a homogeneous ZnO layer thickness of about ≈180 nm for this particular TEM sample (Figure 4a). The ZnO surface observed in Figure 4a is especially flat and not representative of the as‐printed ZnO surface roughness due to the applied FIB polishing nearly parallel to the surface for the preceding EBSD measurements. In contrast, as‐printed ZnO wires have slight thickness variations (see Section S10 and Figure S19b, Supporting Information). The SiO_2_ layer thickness is close to 20 nm. The Si layer below SiO_2_ is about 265 nm thick (refer to Section S10, Supporting Information). The Pt/C layer on top of ZnO is deposited during TEMsample preparation and acts as a protection layer.

A high number density of dark areas with diameters of a few nm is observed in the HAADF‐STEM image, indicative of a locally reduced scattering power (Figure 4a). This reduction in HAADF‐STEM intensity can be explained by the presence of nm‐sized pores, which are also observed in surface‐sensitive SEM imaging (refer to Section S6, Supporting Information). The pores may be caused by the rapid cooling of the ZnO by the moving laser focal spot and the steep temperature gradient during the photothermal laser‐induced printing. Despite the pores, the ZnO layer shows a highly oriented single‐crystalline structure as observed by representative high‐resolution (S)TEM images (Figure 4b,c). Similar to the HAADF‐STEM image in Figure 4a, the cloud‐like intensity variations in the shown high‐resolution images are mainly caused by pores. The high‐resolution TEM (HRTEM) phase‐contrast image of the ZnO crystal lattice in Figure 4b shows a 95 nm × 95 nm wide field‐of‐view taken near the center of the ZnO layer. The corresponding fast Fourier transform (FFT, the squared modulus value of the FFT is shown) of the whole image shows a distinct set of spots resulting from the periodicities of the lattice indicating a single ZnO orientation (see inset in Figure 4b). The lattice spacings fit with the ZnO $\left\langle 2\bar{1}\bar{1}0\right\rangle$ zone‐axis orientation by comparison of the measured lattice spacing and angles with simulations.^[^ ^61^ ^]^ The simulated spot positions are marked with dashed circles in the FFT. In a few HRTEM images, nm‐sized precipitates of either misoriented ZnO or other Zn‐containing crystal phases were detected (refer to Figure S24, Supporting Information). These precipitates seem to be coherently embedded in the ZnO matrix. Due to their small size and relatively low number density (not determined), such precipitates do not contribute significantly to the EBSD signal and were, hence, not observed in our initial EBSD measurements.

The denoised^[^ ^62^ ^]^ HAADF‐STEM image acquired close to the ZnO/SiO_2_ interface shows the same ZnO $\left\langle 2\bar{1}\bar{1}0\right\rangle$ orientation as the HRTEM image (Figure 4c, FFT not shown here). No additional ZnO orientations (or precipitates) are observed in this representative image, confirming that the ZnO film grows primarily with a single orientation from the ZnO/SiO_2_ interface up to the ZnO surface. The ZnO/SiO_2_ interface is not atomically flat but shows thickness variations of a few nm (see dashed arrow in Figure 4c). The SiO_2_ roughness cannot be determined from our TEM analyses since we measure a projection along the electron‐beam direction. The oriented ZnO film grows directly on the amorphous SiO_2_ substrate without forming other interfacial layers in alignment with the results by Choi et al.^[^ ^63^ ^]^ It is reported that a polycrystalline ZnO layer may also form at the substrate interface which then transforms to ⟨0001⟩‐oriented ZnO for larger film thickness.^[^ ^53^, ^58^ ^]^

A small region of the HAADF‐STEM image is magnified in the inset in Figure 4c. At high magnification, the atomic columns of Zn with *Z*  =  30 appear bright in HAADF‐STEM *Z*‐contrast imaging (Figure 4b), and their positions are in good agreement with the ZnO $\left\langle 2\bar{1}\bar{1}0\right\rangle$ zone‐axis orientation (marked with the overlaid model generated with VESTA.^[^ ^64^ ^]^ The atomic columns of O (*Z*  =  8) are not visible due to the relatively low atomic number of O compared to Zn. Additional aberration‐corrected HRTEM imaging at the ZnO/SiO_2_ interface confirmed the amorphous structure of SiO_2_ and Si (Section S11, Supporting Information). The spatial distribution of elements within the cross‐section sample measured by energy‐dispersive X‐ray spectroscopy (EDXS) shows the expected high Zn and O signals in the ZnO layer (Figure 4d). The measured O concentration in ZnO is lower than the expected value of 50 at‐% O for stoichiometric ZnO (Section S10 and Figures S20 and S21, Supporting Information). This aspect can be mostly explained by the X‐ray absorption effects of low‐energy O Kα X‐rays in the relatively thick TEM sample (≈120 to 150 nm). The observed lack of photoluminescence in the visible‐light spectrum also indicates the formation of stoichiometric ZnO without many defects and O vacancies (refer to Section 2.4). Still, O vacancies may be present in the ZnO film which can modify its electronic and optical properties.^[^ ^65^, ^66^ ^]^ The Zn and O signals are reduced at the pore regions (cf. arrows in Figure 4d), indicating that the pores are voids and not filled with Zn‐ or O‐containing residual ink. Minor concentrations of S and C (< 1 at‐%) were detected in ZnO, which likely originate from the DMSO (C_2_H_6_OS, Section S10 and Figures S20 and S21, Supporting Information). However, C may also result from C contamination of the TEM sample and is therefore challenging to measure reliably in trace concentrations. As a result, the genuine C signal from the sample is convoluted with possible C‐contamination signal. Still, the locally increased C signal seems to correlate with the pore positions, which suggests that most C is present in the pores (Figure S22, Supporting Information). The C allotrope could not be determined from STEM‐EDXS measurements.

To investigate the spatial distribution of S (and Ga), we denoised the dataset (see methods) using principal component analysis (PCA) to increase the signal‐to‐noise ratio of the STEM‐EDXS dataset. The PCA‐derived elemental maps for S and Ga are denoted with a star (*) symbol in Figure 4d. Notably, S is only detected in the ZnO film, confirming that residual S is incorporated into the printed ZnO film (cf. S map in Figure 4d). Similar to Zn and O, the S signal is reduced near the pores. This indicates that S is not present as residual DMSO/ink in the pores but is incorporated into the ZnO crystal lattice. Indeed, S‐doping into ZnO is possible without notably affecting the ZnO lattice parameters (see, e.g., references^[^ ^67^, ^68^, ^69^ ^]^ for S). For a few embedded nm‐sized precipitates, the observed lattice spacings fit either with ZnO or with possible Zn‐containing phases such as ZnSO_4_ (refer to Section S12, Supporting Information). However, since *i*) the S signal is homogeneously distributed in the ZnO matrix and *ii*) only a few sparsely distributed precipitates were observed, most S is likely embedded in the ZnO lattice rather than the precipitates. Similar to the possible presence of O vacancies, this may affect the electronic properties of the printed ZnO film.

The elemental maps of Pt and Si in Figure 4d mostly show the location of the protective Pt/C layer, and the Ar^+^‐sputtered SiO_2_ and Si layers. Since the elemental distribution along the horizontal direction does not noticeably vary for these elements, only one‐third of each map is shown for brevity. The SiO_2_ layer shows the expected Si and O signals. For the Si layer, about 2.5 at‐% Ar resulting from prolonged Ar magnetron sputtering was detected (refer to Section S10, Figures S20 and S21, Supporting Information). The PCA‐derived Ga map shows a Ga‐rich surface layer on top of ZnO with a thickness of about 10 nm, which corresponds to the dark line on top of ZnO visible in the HAADF‐STEM images. This layer is amorphized ZnO created during Ga^+^‐FIB polishing of ZnO for EBSD measurements.

As a final aspect regarding TEM, the rotation of the ZnO lattice observed by EBSD is also observed by TEM (see Section S13, Supporting Information for a detailed discussion). Overall, the TEM measurements confirm that the laser‐printed ZnO film is single‐crystalline from the ZnO/SiO_2_ interface up to the ZnO surface. The absence of a polycrystalline ZnO layer at the ZnO/SiO_2_ interface suggests that the crystal grows from the already‐printed ZnO crystal and not from new ZnO seeds on the SiO_2_ surface. Chemical analysis reveals that the pores are voids and that trace concentrations of S (and possibly C) from DMSO are embedded into the ZnO lattice. Additional TEM analyses of an as‐printed ZnO wire (i.e., without any FIB polishing for EBSD measurements) can be found in the supplementary information.

### 2.4 Non‐Linear Optical Effects in Printed ZnO

**Figure 5:**
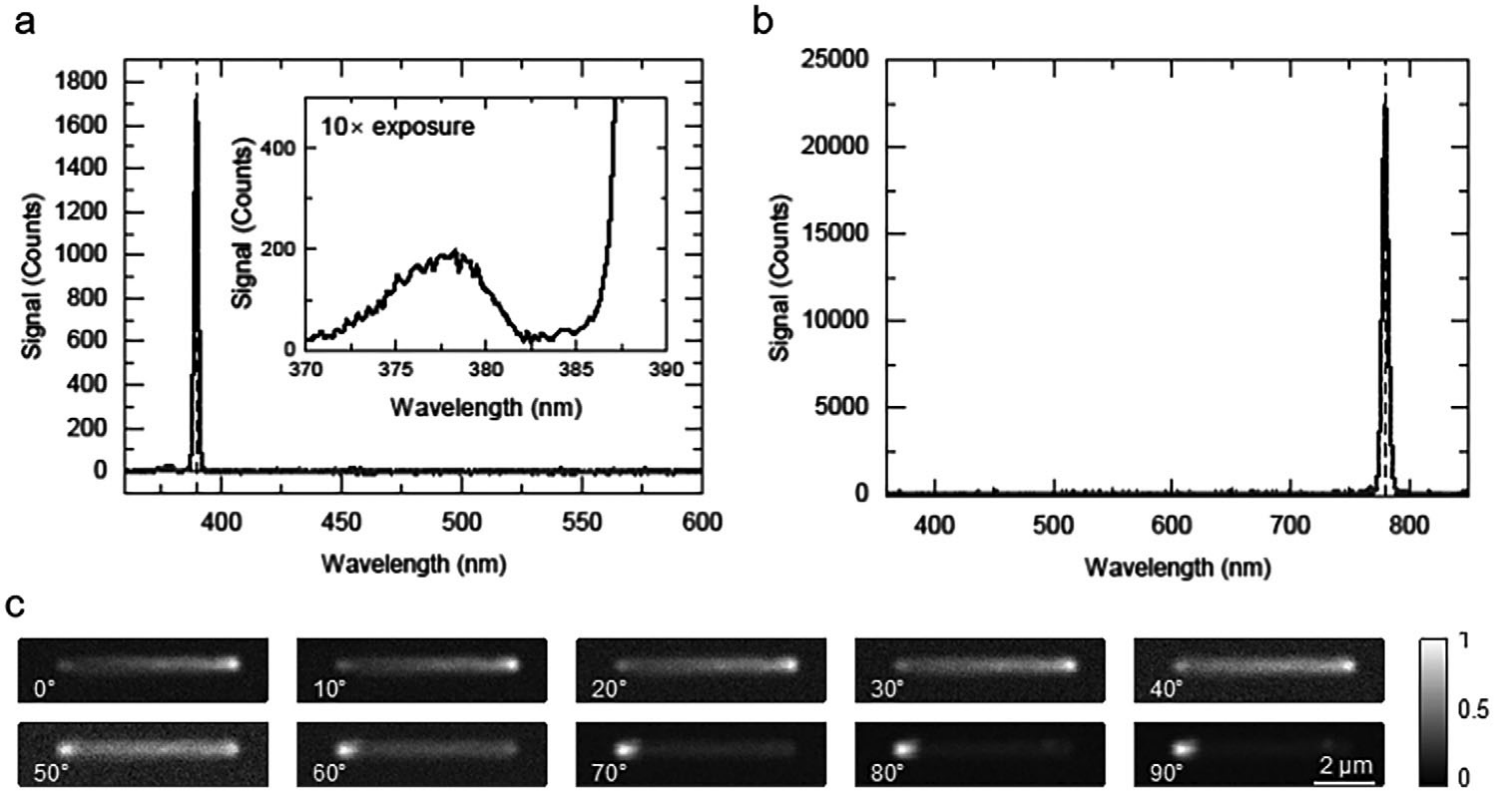
Measurements of second harmonic generation from laser printed ZnO. a) Recorded spectra of SHG from ZnO wire printed at 1 um s^−1^ and 1.85 mW (2.08 MW cm^−2^) printing laser power under 4.6 mW (0.196 TW cm^−2^) exposure from a 780 nm fs laser source. An exposure time of spectrometer CCD was 500 ms. The vertical dashed line indicates 390 nm. Inset shows a zoomed‐in view of an emission peak centered around 377.4 nm using a 10 ×  longer exposure of the CCD camera (5 s). b) Recorded spectra of the 780 nm fs laser used for generating SHG in the ZnO. The vertical dashed line indicates 780 nm. c) Mappings of SHG signal from a laser‐printed ZnO wire (printed right to left) by scanning the sample through the incident fs laser focus. Mapping is repeated for various incident laser polarizations, indicated in the panels, where 0° corresponds to incident polarization perpendicular to the direction of ZnO printing and 90° corresponds to incident polarization aligned with the direction of ZnO printing. The incident power varied from 4.2 mW (0.179 TW cm^−2^) to 3.45 mW (0.147 TW cm^−2^) from 0 to 90° due to some polarization‐sensitive optics in the beam path. Each mapping is normalized to its own maximum signal.

Our electron microscopy analysis has clearly shown the crystalline nature of the laser‐printed ZnO. The observed ZnO wurtzite crystal structure is non‐centrosymmetric. The lack of a center of inversion symmetry is a critical requirement for observing even‐order non‐linear optical effects such as second‐harmonic generation (SHG).^[^ ^16^, ^70^, ^71^, ^72^, ^73^ ^]^ We evaluate this property of our printed ZnO by focusing a fs laser with 780 nm center wavelength onto a ZnO wire using a microscope objective lens (NA  =  0.95) and collecting the reflected/scattered light. The measured SHG signal is shown in **Figure** **5**a. A strong narrow peak centered at 390 nm matches the expected frequency doubling of the 780 nm laser (Figure 5b). A broader, weak emission with a peak at 377.4 nm (using a Gaussian fit) is observed which we attribute to band‐edge luminescence.^[^ ^74^, ^75^, ^76^ ^]^ We observed no photoluminescence in the visible region (refer to Section S14, Supporting Information) which would typically have been indicative of O vacancies and lattice defects.^[^ ^77^, ^78^ ^]^ Since the efficiency of the SHG effect is dependent on the incident light polarization in relation to the material crystal direction, we performed a 2D mapping of the SHG intensity across a ZnO wire for varying incident laser polarizations (Figure 5c). As the incident laser polarization is rotated 90° the intensity profile of the SHG signal subtly shifts from one end of the ZnO wire to the other. We attribute this change in SHG efficiency along the wire to the rotation of the crystal axis described above.

## 3 Conclusion

We introduce the first photothermal laser‐printed sub‐µm single‐crystalline semiconductor, ZnO, which requires no post‐processing and consists simply of a single precursor in a solvent. Electron microscopy analyses confirm the single‐crystalline ZnO structure with a continuous ZnO lattice rotation. The latter depends on the starting orientation and relative changes in the printing direction, but no comprehensive theory for predicting the lattice rotation was found. Printing of these single semiconductor crystals is not limited to straight wires but can be extended to various 2D curves and geometries providing considerable flexibility in the design of future devices. Furthermore, the non‐linear optical and piezoelectric properties enabled by the crystal structure of the printed ZnO make it a new option for the additive manufacturing of novel micro‐optical and micro‐electronic devices. The relatively low laser powers required for printing and the use of a CW laser make the capability for printing site‐specific single microcrystals widely accessible in a format that can be readily translated to a multi‐material printing system utilizing, for example, a microfluidic ink‐exchanging system. The potential applications for these printed submicron single crystalline ZnO include micro‐lasers,^[^ ^79^ ^]^ nano‐wire transistors,^[^ ^80^ ^]^ and nanogenerators.^[^ ^81^ ^]^ We anticipate that our methodology will enable the direct laser printing of microelectronics in a much more affordable and user‐friendly fashion.

## 4 Experimental Section

### Materials

All chemicals and solvents were used as received from the supplier without further purification. Zinc formate (98% Thermo Fisher), DMSO (dry over 4 Å molecular sieves, 99.8% Acros Organics), isopropanol (≥99.9% Carl Roth GmbH), Deconex OP 146 (4 vol‐%), Deconex OP 12PA‐x (2 vol‐%) and Deconex 171 (2 vol‐%) (Borer Chemie AG).

### Ink formulation

A quantity of 500 mg (Zn(HCO_2_)_2_) (3.22 mmol) was filled in a crimp cap vial (20 mL, 75.5 × 22.5 mm), closed, and dispersed with 10 mL dry DMSO (septum bottle over 4 Å molecular sieves). The vial was immersed into a sonicator for 15 min and submitted to an additional 4 h of agitation in an orbital shaker at 30 °C (400 rpm). Afterward, the suspension is left for 12 h without agitation at ambient temperature. The suspension is then filtered through a 0.20 µm hydrophobic PTFE syringe filter (Ø = 25 mm, VWR) into another 20 mL crimp cap vial previously purged with a stream of nitrogen. The ink is stored in the crimp cap vial prior to use.

### Photothermal Laser Printing

A 532 nm CW laser (Coherent, Verdi‐V5) was used for the laser printing. The laser power was modulated using an acoustic‐optic modulator (AA Opto Electronic, MT80‐A1.5‐VIS). The beam was then expanded to overfill the rear aperture of an oil‐immersion microscope objective lens (Zeiss, Plan‐APOCHROMAT 100 × /1.4 Oil) which focused the laser, using type F (*n* = 1.518) immersion oil between the lens and the glass coverslip window, onto the substrate for printing. The objective was mounted on a piezoelectric stage (Physik Instrumente, P‐733.ZCL, 100 µm travel) to translate the focus along the optical axis. The sample was translated horizontally using a combination of a piezo stage (Physik Instrumente, P‐734.2CL, 100 µm × 100 µm travel) and a motorized stage (Physik Instrumente, PM686.D64, 25 mm × 25 mm travel). The sample interface was determined by using reflection of a weak 675 nm diode laser (Thorlabs, LPS‐PM675‐FC), introduced via dichroic beamsplitter (AHF, zt 640 RDC), off the sample surface in a confocal detection scheme. In situ monitoring of the print process was done in transmission illumination mode using a USB camera (FLIR, BFS‐U3‐50S4C‐C) and a red LED source (Thorlabs, M625F2). Quoted laser powers correspond to the measured power passing through an ≈5.5 mm aperture placed at the objective lens location. The intensity values provided are the peak values at the laser focus assuming a Gaussian profile with the full‐width half maximum determined via gold bead scanning (see Section S5, Supporting Information) and an assumed transmission of the objective lens of 93%. The scanning speed during printing was 1 µm s^−1^ unless otherwise noted. The printing system was controlled via custom software using MATLAB and NI data acquisition cards (National Instruments, PCIe‐6351 and PCIe‐6353).

### Sample Preparation and Development

For printing substrates, borosilicate glass coverslips (Marienfeld, 1.5H 22 mm × 22 mm ×  0.17 mm) were first cleaned by sonication at 60 °C in individual aqueous solutions of 4 vol‐% Deconex OP 146, 2 vol‐% Deconex OP 12PA‐x, and 2 vol‐% Deconex OP 171, respectively. Each cleaning step was followed by sonication in water. All sonication steps were for 10 min and only ultrapure water was used. Substrates were then blown dry with nitrogen and baked at 120 °C to remove residual water. A Si layer was then deposited onto the substrates using magnetron sputtering (BOC Edwards, Auto 500) with a power of 200 W and 3.5 SCCM argon inflow. A second sputtering step of 100 W and 7.5 SCCM of atmosphere inflow was used to deposit 18 nm SiO_2_ on top of the Si. Substrates were mounted in the sandwich mode for printing with the ink contained between the sputtered side of the substrates and an additional unmodified coverslip using one layer of Kapton tape (thickness 61 µm) as a spacer. After printing, samples were developed in 20 mL baths of DMSO for 10 min, isopropanol for 10 min, and again in isopropanol for 10 min. Samples were then blown dry with nitrogen.

### Sample Characterization—Second Harmonic Generation (SHG)

For the SHG measurements, a 780 nm fs laser (Coherent, Chameleon Ultra II) was introduced into the same setup used for printing. The 780 nm laser was combined with the printing laser path through the interface finding path via a dichroic mirror (AHF, T 680 DCSPXR). The laser power was modulated using an acousto‐optic modulator (AA Opto Electronic, MTS40A3750.850) and expanded to overfill the rear aperture of an air microscope objective lens (Leica, HC PL FLUOTAR 100 × /0.95 CORR). The developed samples after printing were remounted in the sandwich mode without ink (air gap). Light from the sample was recollected by the objective lens and 10% (AHF, F21‐032SG) was diverted for detection. Spectrometer measurements from the sample were recorded by focusing the light with a 50 mm focal length lens into a spectrometer with a liquid nitrogen‐cooled CCD (CHROMEX, Inc., 250i; Princeton Instruments, 7509–0002). The entrance slit of the spectrometer was 10 µm and internal grating was set to blaze 390 nm. Two glass 335–610 nm bandpass filters (Thorlabs, FGB37 M) were used before the lens to reject any residual fundamental 780 nm light. For the SHG mappings, the diverted light was instead passed through a glass bandpass filter (Thorlabs, FGB37 M) and a 390 nm narrow bandpass filter (Thorlabs, FBH390‐10) before focusing with a lens (Thorlabs, AC254‐030‐A‐ML) onto an avalanche photodetector (APD) (Laser Components, A‐Cube‐SU500‐01). During the measurements, the laser was modulated at 4 kHz with a 50% duty cycle square wave. The APD signal was measured with a lock‐in amplifier (Stanford Research Systems, SR830). All quoted laser powers are measured through a 6 mm aperture located at the objective lens position using 100% duty cycle of the laser. The 780 nm fundamental laser spectrum was measured by scattering the laser into a compact spectrometer (Ocean Optics, USB4000, 100 ms integration time). The intensity values provided are the peak values at the laser focus assuming a Gaussian profile with the full‐width half maximum determined via gold bead scanning (see Section S5, Supporting Information), a 140 fs pulse width, 80 MHz repetition rate, and an assumed transmission of the objective lens of 88%.

### Sample Characterization—Electron Backscatter Diffraction Analysis (EBSD)

A Thermo Scientific Helios G4 FX combined FIB and SEM dual beam system was used to characterize the crystallinity and crystal orientation of the printed ZnO with EBSD. The latter technique requires a polished sample surface, for which FIB milling was used. First, the sample is mounted on a 45° pre‐inclined holder. Then, the sample is tilted so that the sample surface is aligned at an angle of 0.3° relative to the FIB pole piece. The sample is then polished at this angle using a low nominal FIB current between 26 and 90 pA at 30 keV. Following the polishing process, the sample is mounted on a 10° inclined holder and tilted at an angle of 60°, and a working distance of 15 mm is set.^[^ ^82^ ^]^ The effective sample tilt is then 70°. The EBSD patterns were acquired at an electron energy of 20 keV and a nominal electron‐beam current of 6.4 nA using a Bruker eFlash HD detector. The Bruker Esprit 2.1 software was used for the collection of the EBSD patterns. EBSD data processing was performed with the Bruker Esprit 2.3 software. The Kikuchi wires in the EBSD patterns were indexed using the Hough transform. The lattice parameters *a*, *b*, and *c*, utilized for indexing the hexagonal ZnO wurtzite structure (*P*6_3_ *mc*, space group 186), are 3.243, 3.243, and 5.195 Å. Only patterns with a minimum of 6 indexed bands and a maximum deviation angle of 1.5° were considered for subsequent orientation analysis using the MTEX toolbox (version 5.10.2)^[^ ^83^ ^]^ for MATLAB. The analysis of these patterns provides PF, IPF maps, and misorientations.^[^ ^84^ ^]^ PFs are shown in upper and equal‐angle projections. Kikuchipy^[^ ^85^ ^]^ and Fiji^[^ ^86^ ^]^ were used for indexing and creating the video of (indexed) EBSD patterns for the different printed angles.

### Sample Characterization—Transmission Electron Microscopy (TEM)

Cross‐section samples for TEM were prepared using the in situ lift‐out technique in an FEI Strata 400S Ga^+^‐ion FIB/SEM instrument.^[^ ^87^ ^]^ First, two wires were milled with a small nominal FIB current of 9 pA on the sides of the printed ZnO wires to mark the center of the ZnO wires for the final thinning steps (refer to Figure S27, Supporting Information). Then, Pt/C protection layers were deposited by SEM and FIB to protect the underlying material during FIB milling. A low Ga^+^‐ion energy of 3 keV was used during the final polishing steps to minimize the thickness of amorphous surface layers on the TEM sample caused by FIB milling.^[^ ^88^, ^89^ ^]^

HRTEM images and selected‐area electron diffraction (SAED) patterns were acquired on an FEI Titan^3^ 80–300 operated at 300 keV on a TVIPS TemCamXF‐416 (R) camera. The ZnO crystal structure was analyzed by comparing experimental and simulated diffraction patterns and Fourier transforms (FT) from HRTEM images. ReciPro^[^ ^61^ ^]^ was used to index and simulate the diffraction patterns with crystallographic information files obtained from the inorganic crystal structure database (ICSD).^[^ ^90^ ^]^

An FEI Tecnai Osiris equipped with ChemiSTEM technology^[^ ^91^ ^]^ operated at 200 keV was used for STEM‐EDXS measurements and high‐resolution HAADF‐STEM imaging. The high‐resolution HAADF‐STEM images were denoised using a pre‐trained deep‐learning model.^[^ ^62^ ^]^ A convergence semi‐angle of about 10.7 mrad was used. The HAADF‐STEM signal (collection semi‐angle of about 57–200 mrad) was used for sample‐drift correction during STEM‐EDXS acquisition with the Bruker Esprit 1.9 software. Subsequent STEM‐EDXS data processing was performed using Bruker Esprit 2.3 and HyperSpy.^[^ ^92^ ^]^ The shown elemental maps display background‐corrected X‐ray‐peak intensities. For showing the distribution of low‐concentration elements such as S and Ga in Figure 4, a 5 × 5 pixel binning was first applied, followed by a weighting of neighboring EDS spectra with a Gaussian kernel, and PCA to increase the signal‐to‐noise ratio of the STEM‐EDXS dataset.^[^ ^93^, ^94^ ^]^ Peak fitting is then used to separate overlapping X‐ray transitions, e.g., S Kα (2.31 keV) and Pt Mγ (2.33 keV). The chemical concentrations were quantified from summed‐up raw EDS spectra using a standardless Cliff‐Lorimer approach.^[^ ^95^ ^]^ No absorption correction was applied since the sample thickness and density of the different sample layers were not known with reasonable accuracy.

### Sample Characterization—Scanning Electron Microscopy (SEM)

Some SEM imaging was performed with a Zeiss Leo 1530 scanning‐electron microscope operated at 10 keV electron energy. Prior to imaging, the samples were coated by a ≈10 nm thin Au layer.

### Statistical Analysis

Average rotation rates in degree per µm were calculated from *i*) the scalar misorientation values calculated by MTEX and *ii*) the distance between two selected points in the misorientation maps (start and end of the primary orientation). The exact positions were chosen manually and a possible polycrystalline region at the start was treated as an outlier and neglected. No further pre‐processing of the data was performed. The average rotation rates for the same structure and processing parameters (wires, angles, or circles) were averaged. The statistical dependence of these averaged rotation rates was evaluated with a two‐sided *t*‐test for two independent samples and a significance level of α  =  0.05 using the *scipy* Python library (*scipy.stats.ttest_ind* function).^[^ ^96^ ^]^ A homogeneous variance was assumed, which was checked and confirmed using Bartlett (*scipy.stats.bartlett*) and Levene (*scipy.stats.levene*) tests. In all tested cases, the resulting *p*‐value was greater than α, meaning that the observed differences in average rotation rates between the different printed structures are not statistically significant.

## Conflict of Interest

The authors declare no conflict of interest.

## Author Contributions

M.S., P.S., K.K., and L.G. contributed equally to this work. M.S., P.S., and F.F. conceived and designed the ink formulations and fabricated all the samples. M.S., P.S., and S.K. designed the substrates. M.S. and F.F. designed the ZnO ink. M.S., P.S., S.K., F.F., K.K, and L.G. analyzed the size and quality of the samples. K.K., L.G., and Y.M.E. designed the workflows for electron diffraction and microscopy and interpreted the results on the local structure, crystallinity, and chemical composition of the printed samples at the nm‐scale. All authors participated in the discussion, interpretation of the data, and drafting of the manuscript. M.W., C.B.K, C.F., and Y.M.E. conceptualized the project and led the study.

## Supporting information



Supporting Information

Supplemental Video 1

## Data Availability

The data that support the findings of this study are available at Zenodo.
