# Supplementary material for: Photothermal Laser Printing of Sub‐Micrometer Crystalline ZnO Structures
Source: Adv Sci (Weinh). 2024 Dec 4;12(4):2410771. doi: 10.1002/advs.202410771 (PMC11789601; doi:10.1002/advs.202410771)
Supplement: Supplementary file 1 — Supporting Information [file ADVS-12-2410771-s001.docx]

Supporting Information

Photothermal Laser Printing of Sub-micrometer Crystalline ZnO Structures

Matthias Steurer^‡^, Paul Somers^‡^, Kristian Kraft^‡^, Lukas Grünewald^‡^, Steven Kraus, Florian Feist, Bastian Weinert, Erich Müller, Stephanie Dehnen, Claus Feldmann,* Yolita M. Eggeler,* Christopher Barner‑Kowollik* and Martin Wegener*

Table of Contents

S1. Absorbance of zinc formate in DMSO 3

S2. Substrate Si layer thickness 4

S3. Identification of the printing window 7

S4. Influence of laser power and printing speed on ZnO crystallinity and quality 8

S5. Measured laser spot sizes 12

S6. ZnO wire cross-section 14

S7. EBSD on different geometries 15

S8. Texture analysis of all investigated straight wires 19

S9. Electron Backscatter Diffraction Analysis of a 100 µm long wire 26

S10. Chemical Analysis by Energy-dispersive X-ray Spectroscopy (EDXS) 28

S11. HRTEM of the SiO_2_/ZnO Interface 36

S12. Crystallites/Grains observed by HRTEM 38

S13. ZnO Lattice Rotation Analysis in the TEM 40

S14. Photoluminescence of printed ZnO 44

S15. TEM-sample preparation by FIB milling 45

S16. References 47

# Absorbance of zinc formate in DMSO

The ultraviolet-visible (UV-Vis) absorbance of the zinc formate in the DMSO solution was measured using the ZnO ink that was used for printing. The measurement was carried out using a Cary 5000 spectrophotometer with the slit set to full and using 100% energy. A cuvette with 10 mm pathlength was used. A blank was measured with a cuvette filled with only extra dry DMSO and then subtracted from the measured sample. The resulting spectrum is shown in **Figure S1**. There is no absorption at the 532 nm laser wavelength as well as most of the visible range.


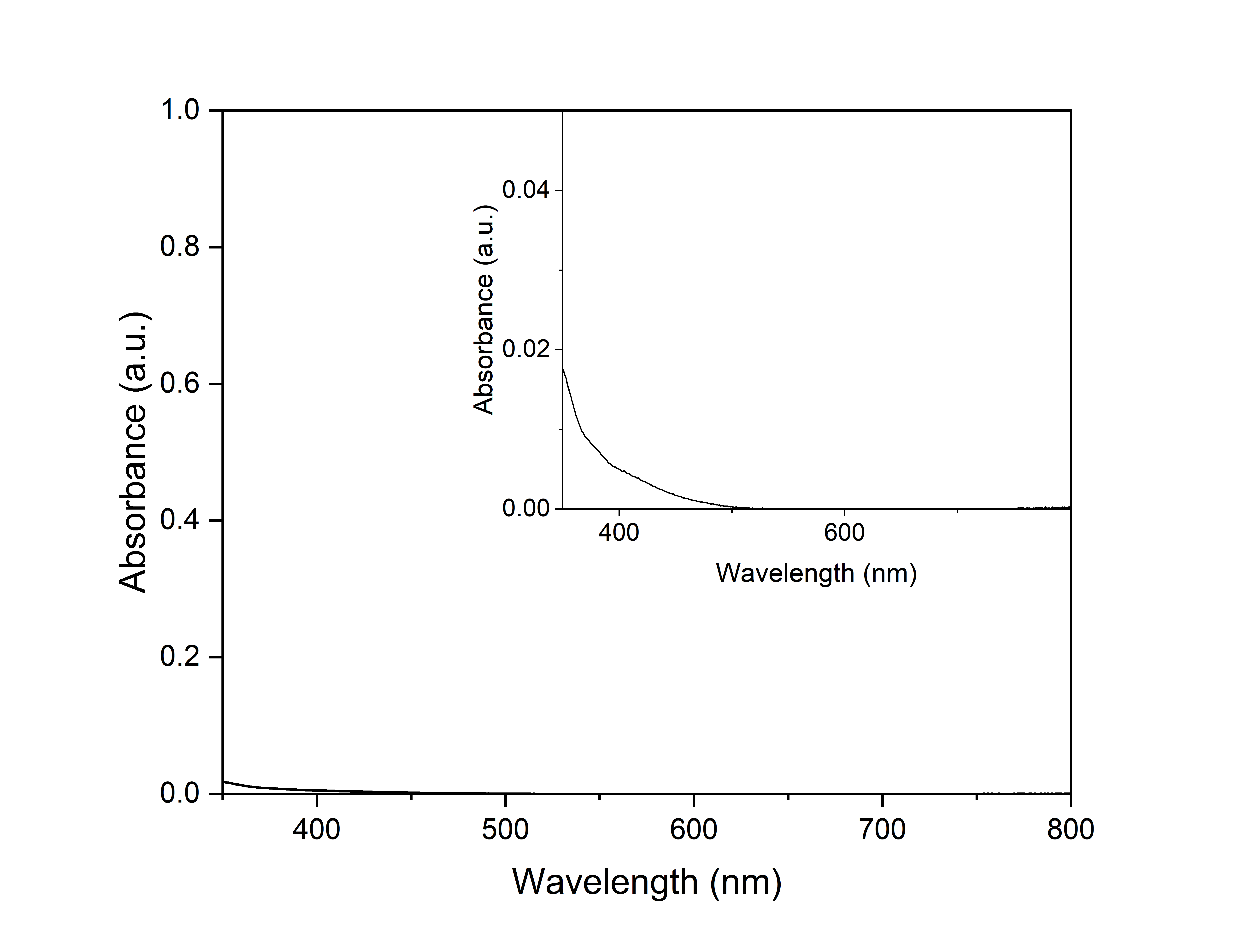


**Figure S1.** Absorbance of zinc formate in DMSO as a saturated solution. Inset shows a magnified view of the data.

# Substrate Si layer thickness

We performed simulations of the maximum temperature change Δ*T* at the ink/substrate interface under constant printing laser power and varying Si layer thickness using COMSOL. Two cases were considered: crystalline Si like a commercial wafer and amorphous Si from magnetron sputtering (**Figure S2**). The plot clearly shows that amorphous silicon reaches significantly higher temperature changes. There is also a Si thickness that provides a peak temperature change followed by a decreased temperature with further increasing Si thickness. This can be rationalized by the fact that there is an increase in the conduction of heat away from the laser spot as the heat can begin moving perpendicular to the surface as well as in the plane of the Si layer. The crystalline silicon experiences very little temperature increase over the range of thicknesses tested. It is thus not surprising that for printing on a silicon wafer that printing cannot be observed within the same laser power ranges as those used for printing on a sputtered Si layer. The curves in **Figure S2** are intended primarily as a qualitative representation as the COMSOL model does not fully capture all the dynamics that occur during printing. The key parameters for the simulation are given in **Table S1**, while its setup is as follows.

The simulations were carried out as a time-dependent study using the parameters in **Table S1** and the “Heat Transfer in Solids and Fluids” interface. The model is set up as a rotationally symmetric 2D simulation, therefore simulating a spherical shape composed of 4 material layers. These are oriented perpendicular to the laser propagation direction, with infinite elements at the outer boundaries. At the center point, where the laser meets the silicon layer, the mesh needs to be sufficiently fine to resolve the smallest feature. The element size therefore starts at sub-nm values and greatly expands to several hundred nm towards the simulation boundaries. The simulation uses an ambient starting temperature of 22 °C. For each silicon layer thickness, the dynamics are simulated until a time of 25 ms, following which there are no further observable changes in the temperature profile. Similar to the conditions during printing, the order of the layers in the direction of laser propagation is DMSO, silica, silicon, and glass. For computational efficiency, the laser is implemented as a shaped heating source only affecting the silicon layer. The shape corresponds to a Gaussian profile with attenuation perpendicular to the interface. According to literature, parameters of silicon can vary depending on fabrication circumstances, temperature, and film thickness. For amorphous Si, the values are taken from literature (cf. **Table S*1***). For crystalline Si, variable parameters do not significantly affect the maximum temperature changes. As such, all parameters for crystalline Si are kept constant at their room temperature and bulk value throughout the simulation.


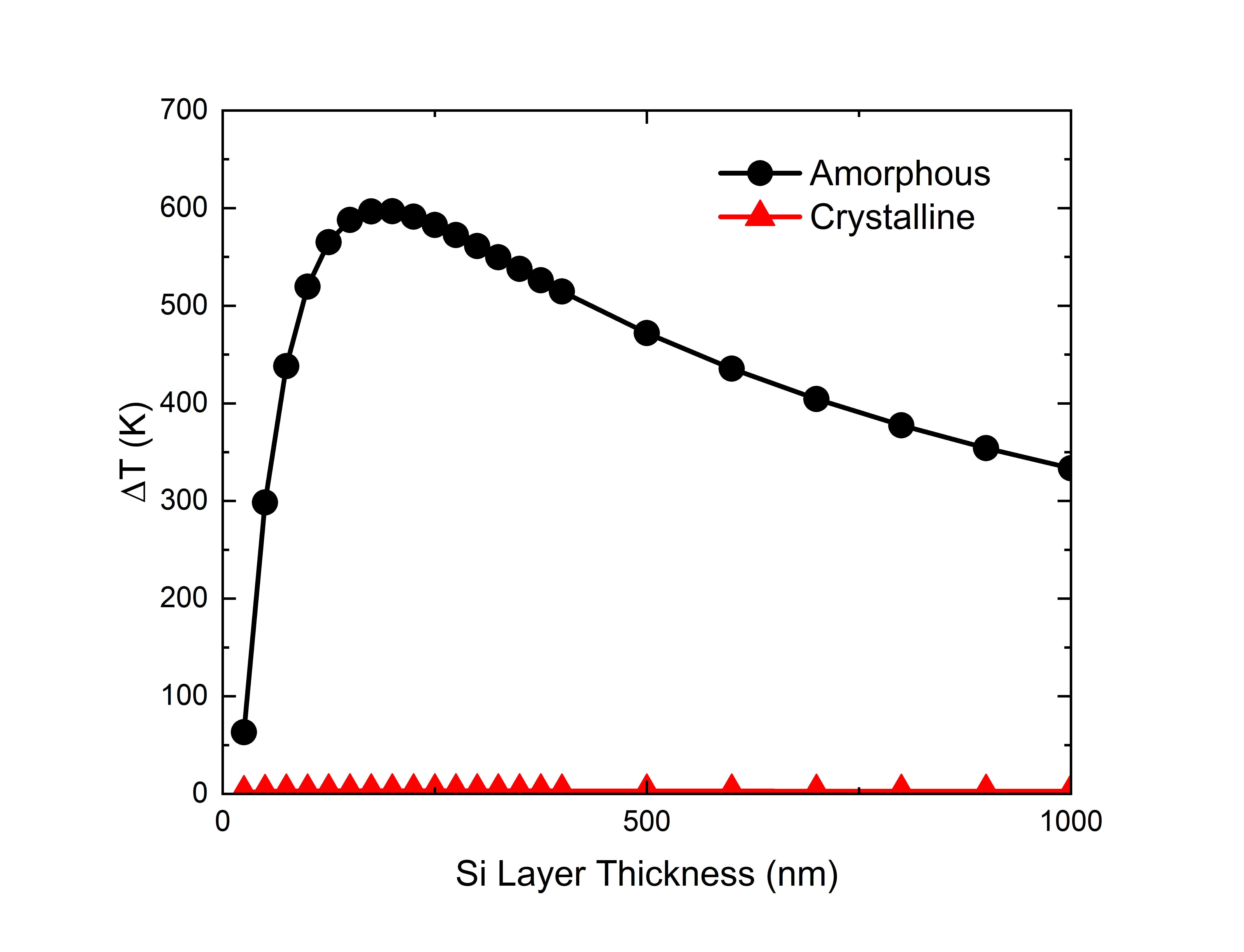


**Figure S2.** Simulation of maximum temperature change at the surface of the ink/substrate interface for varying Si layer thicknesses for an incident laser irradiation of 1 mW and focusing conditions similar to the experimental setup.

**Table S1.** COMSOL parameters for Si layer thickness simulation.

| Parameter | Value | Unit | Reference |
| --- | --- | --- | --- |
| silica thickness | 20 | nm |  |
| silica thermal conductivity | 1.4 | W∙m^-1^∙K^-1^ | COMSOL |
| silica specific heat | 730 | J∙kg^-1^∙K^-1^ | COMSOL |
| silica density | 2200 | kg∙m^-3^ | COMSOL |
| DMSO thermal conductivity | 0.18 | W∙m^-1^ ∙K^-1^ | ^[1]^ |
| DMSO specific heat | 1912 | J∙kg^-1^∙K^-1^ | ^[2]^ |
| DMSO density | 1100 | kg∙m^-3^ | ^[3]^ |
| substrate thermal conductivity | 0.96 | W∙m^-1^∙K^-1^ | ^[4]^ |
| substrate specific heat | 820 | J∙kg^-1^∙K^-1^ | ^[4]^ |
| substrate density | 2510 | kg∙m^-3^ | ^[4]^ |
| laser power | 1 | mW |  |
| laser radius | 250 | nm |  |
| laser wavelength | 532 | nm |  |
| laser profile | Gaussian |  |  |
| crystalline silicon thermal conductivity | 130 | W∙m^-1^∙K^-1^ | ^[5]^ |
| amorphous silicon thermal conductivity^a^ | 1.1-2.7 | W∙m^-1^∙K^-1^ | ^[6]^ |
| crystalline silicon 90% extinction | 2867 | nm | ^[7]^ |
| amorphous silicon 90% extinction | 176 | nm |  |
| silicon specific heat | 711 | J∙kg^-1^∙K^-1^ | ^[8]^ |
| silicon density | 2329 | kg∙m^-3^ | ^[9]^ |

a Thermal conductivity for varying film thickness taken from reference [6].

# Identification of the printing window

During initial printing tests, the printing parameters including speed and laser power were varied to identify the optimal conditions. An example of one of the test prints is shown in **Figure S3**. In a test print, several short wires are printed at varying laser focus heights relative to the substrate, along the horizontal direction in the figure, ensuring that proper focusing of the print laser on the substrate is achieved during the test. This is repeated for increasing laser powers, along the vertical direction in the figure. All the wires in each print test are printed at the same speed. The following section discusses the influence of printing speed on the shape and crystallinity of the ZnO wires*.*


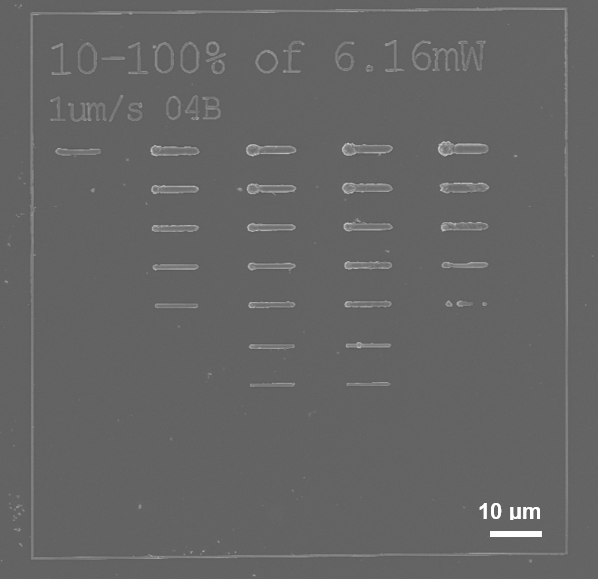


**Figure S3.** Example of a test print for searching the printing parameter space. Printing speed was 1 µm s^-1^ and laser power was stepped in 10% increments of a total of 6.16 mW (6.94 MW cm^-2^).

# Influence of laser power and printing speed on ZnO crystallinity and quality

Different laser powers and printing speeds were tested to produce ZnO wires. **Figure S4** shows backscattered electron (BSE) SEM images, IPF-X, and IPF-Y maps for 5 different printing speeds (1 to 40 µm s^-1^) and for laser powers of 2.16 (2.43), 3.08 (3.47) and 4.00 (4.50) mW (MW cm^-2^). The IPF-Z map is not shown as it is similar to the IPF-X and IPF-Y map. The printing direction is set to align along the real-space direction *x*. The printing speed increases for each row from top to bottom. In addition, the plots in **Figure S5** show the measured width and height from the wires in dependence of the printing parameters. Overall, lower printing speeds lead to more deposited material, resulting in wider and thicker ZnO wires (left column in **Figure S4** and **Figure S5**). At lower printing speeds, the laser is focused on the substrate surface for a longer duration, resulting in a longer time for ZnO crystal growth. Noticeable at 5 µm s^-1^ (and also for 1 µm s^‑1^) printing speed is that a larger amount of ZnO has been deposited at the beginning of the ZnO wire compared to the rest of the wire. This may be caused by the initially isotropic heat profile from the substrate on the surface (i.e., in the *x-y* plane), leading to a symmetrical, circular ZnO formation. Afterwards, the heat profile is modified by the presence of already-deposited ZnO upon printing. The shape of the 10 µm s^-1^ wire is not uniform, which can have various causes: vibrations may have disturbed the printing process, contamination on the substrate surface (or in the ink), or a nonuniform temperature profile due to changes in heat dissipation as ZnO is deposited. In contrast to the 10 µm s^-1^ wire, the 20 and 40 µm s^-1^ wires have a more uniform shape.

The IPF-X and IPF-Y maps for the 1 µm s^-1^ wire show an initial grain with a different orientation that then changes to the larger, primary ZnO orientation upon printing. The latter has a color gradient, indicating the rotation of the ZnO single crystal. White pixels in the IPF maps of the ZnO wires show unindexed patterns.

Similarly, the IPF maps of the ZnO wire printed at 5 µm s^-1^ show only a single grain with a rotation. It is noticeable here that compared to the wire printed at 1 µm s^-1^, there are fewer unindexed white pixels. The higher indexing fidelity of the EBSD patterns indicates a higher quality of the ZnO crystal. A possible explanation for this may be related to the rotation rate. For the 1 µm s^-1^ ZnO wire, the rotation rate is 2.93° µm^-1^ and for the 5 µm s^-1^ wire it is 1.45° µm^‑1^. The reduced rate may help with the crystalline quality leading to more indexed pixels for 5 µm s^-1^ printed wires.

The 10 and 20 µm s^-1^ wires show that higher printing speeds lead to an increased number of unindexed patterns. For the 40 µm s^-1^ wire, it was not possible to record any EBSD patterns. However, the experimental artifacts from FIB preparation or possibly a higher porosity of these printed wires might also influence the EBSD results, which are used to determine the crystallinity. For higher printing speeds crystalline ZnO may not form. A similar effect was observed by other groups for even higher printing speeds (200 µm s^-1^) which may result from different experimental setup and material.^[10]^ Other groups have observed that in molten glass for higher printing velocities (40 to 200 µm s^-1^) crystallization growth of uniform crystal structures occurs. Lower speeds resulted in the lattice rotation deviating from the printing direction. This is due to the formation of large angular grain boundaries, as crystal growth is faster and dominant in the <0001> direction.^[10]^ This aspect is in contrast to our findings for our experimental setup, where lower printing speeds (1 to 5 µm s^-1^ showed the highest crystallinity) and may be explained by different experimental setup and materials.

Finally, the reduced wire heights for faster printing speeds (**Figure S5**) make it more challenging to polish the ZnO surfaces without removing too much material for subsequent EBSD measurements. This can then cause variations in indexing quality in the IPF maps.

For higher printing speeds, the single crystalline growth experiences a change in crystallinity due to the limitations in the diffusion of chemical components. The available time for the diffusion of the atoms to the growth front is significantly reduced. Therefore, there is not enough time for the atoms to migrate and arrange into a crystalline structure. As a result, the formation of stable nucleation seeds becomes less likely, hindering the growth of a crystal lattice.^[10]^ Qualitatively, for higher print speeds, we also observe a general trend of increasing wire roughness and poor wire quality which can be attributed to a diffusion-limited process during printing. Further, at higher speeds, larger laser powers are required to supply a necessary energy dose and at some point, the laser power cannot go above a power which will cause a temperature increase large enough to induce boiling of the solvent. Thus, while laser power can be increased to compensate the energy dose required for material deposition during high-speed printing, the solvent boiling point and chemical diffusion are the ultimate limiting factors for maintaining continuous high-quality wires.


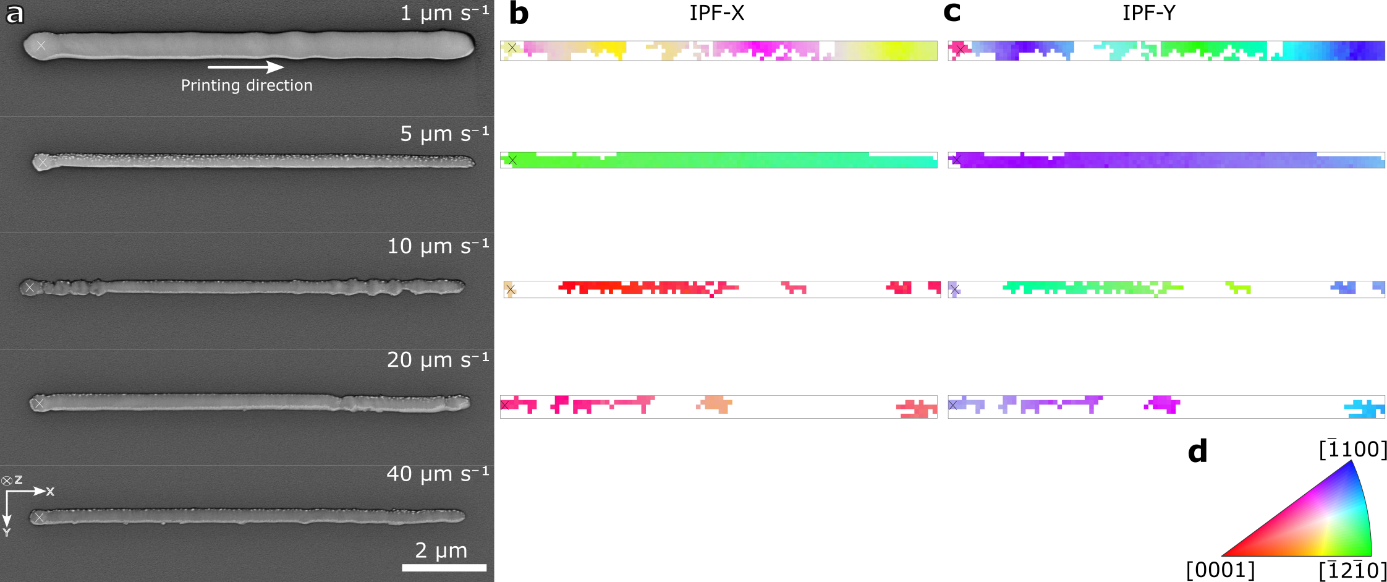


**Figure S4.** **a** Top-view BSE-SEM images of polished ZnO wires printed at different printing speeds. The printing start position is marked with a cross. **b** and **c** Corresponding IPF-X and IPF-Y maps of the polished ZnO top surface. **d** Color legend for the IPF maps.

Furthermore, we investigated the width and the height of the printed ZnO wires at different laser powers and printing speeds from SEM images (**Figure S5**). The wire height was measured by tilting the sample by 52° and acquiring SEM images (not shown here). To determine the actual height from the SEM images, a tilt correction was applied to the measured height. In **Figure S5a**, the width of the ZnO wires is shown for the different printing parameters. One can conclude that, on the one hand, a higher laser power leads to an increasing width. On the other hand, higher printing speed leads to a decrease in average width. Similar observations were made for the height of the ZnO wires (**Figure S5b**). A higher laser power leads to an increasing height of the ZnO wires. An increase in the printing speed reduces the height of the wires. These observations are consistent with the idea that higher printing rates lead to less ZnO deposition. Similar results were observed for the growth of ZnO nanowires created by the decomposition of zinc acetate on ZnO nanoseeds.^[11]^


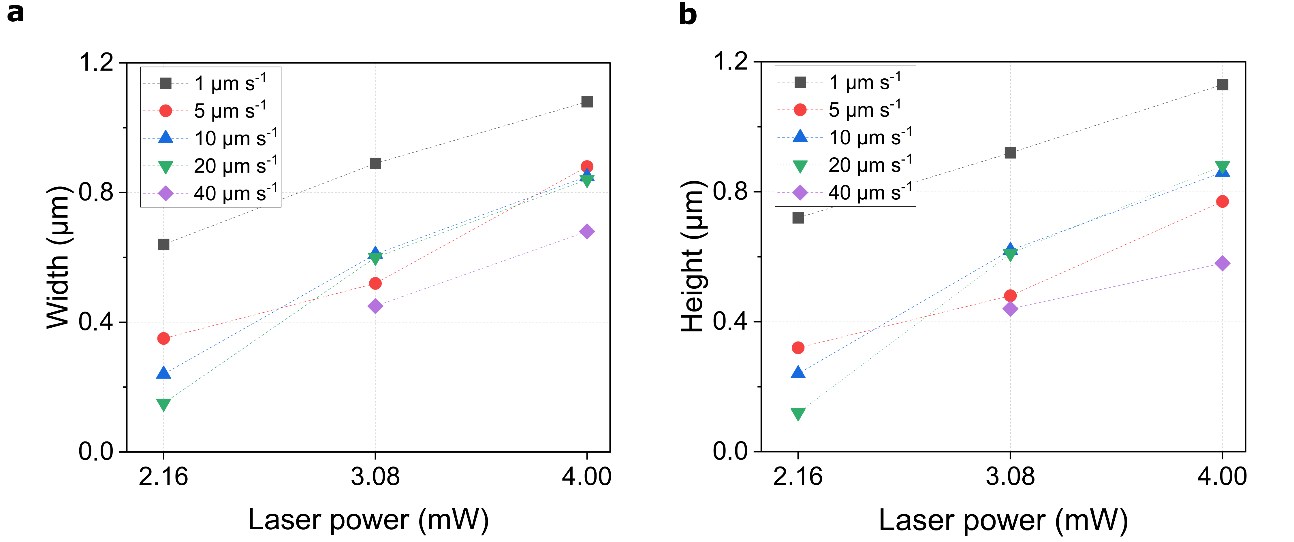


**Figure S5.** **a** Width in µm of ZnO wires printed at 2.16 (2.43), 3.08 (3.47) and 4.00 (4.50) mW (MW cm^-2^) laser power (intensity) for 1, 5, 10, 20 and 40 µm s^-1^ printing speed. **b** ZnO wire height in µm printed at different laser power and printing speeds.

# Measured laser spot sizes

The laser spot size used for printing was experimentally measured by scanning the laser focus with an 80 nm diameter gold bead. In order to as closely match the printing conditions as possible, the measurement was done in the following way. The surface of a glass coverslip was functionalized with thiol groups and coated with a sparse arrangement of gold beads. The gold bead coated coverslip was mounted in the same way as **Figure 1**, where the surface with the gold beads was in the same position as where the Si/SiO_2_ layers would normally be located during printing. A single gold bead on the surface of the coverslip was scanned through the laser focus and the backscattered light was collected with an avalanche photodetector (similar setup as used in **Figure 5c**). The resulting scans are shown in **Figure S6**. A Gaussian fit was performed across the center of **Figure S6a** yielding a full width at half maximum (FWHM) spot size of 270 nm.

Similarly, the laser spot size of the 780 nm laser used for performing the SHG experiments was measured using a 200 nm gold bead. A larger gold bead was required to increase the backscatter signal of the gold bead to overcome the background reflection off the glass coverslip in air conditions. The sample was again prepared to as closely match the experiment conditions as possible and the 0.95 NA objective was used. The resulting scans are shown in **Figure S7**. We attribute the low intensity artifact in the scans to a polarization dependent response of the large gold beads. A Gaussian fit was performed across the center of **Figure S7a** yielding a FWHM spot size of 405 nm.


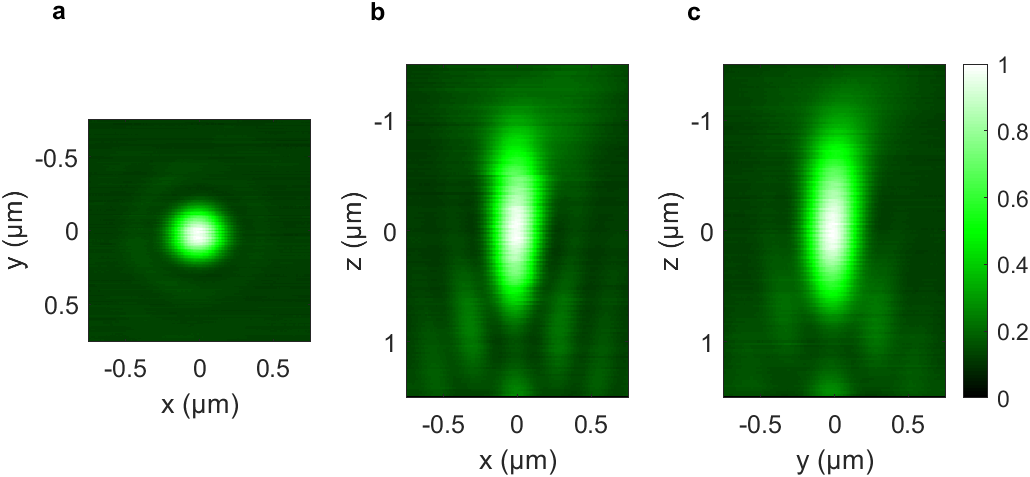


**Figure S6.** Printing laser beam profile as measured by scattering from an 80 nm gold bead. Data is normalized to the peak value. Each panel is a 2D slice in each of the planes **a** XY, **b** XZ, and **c** YZ.


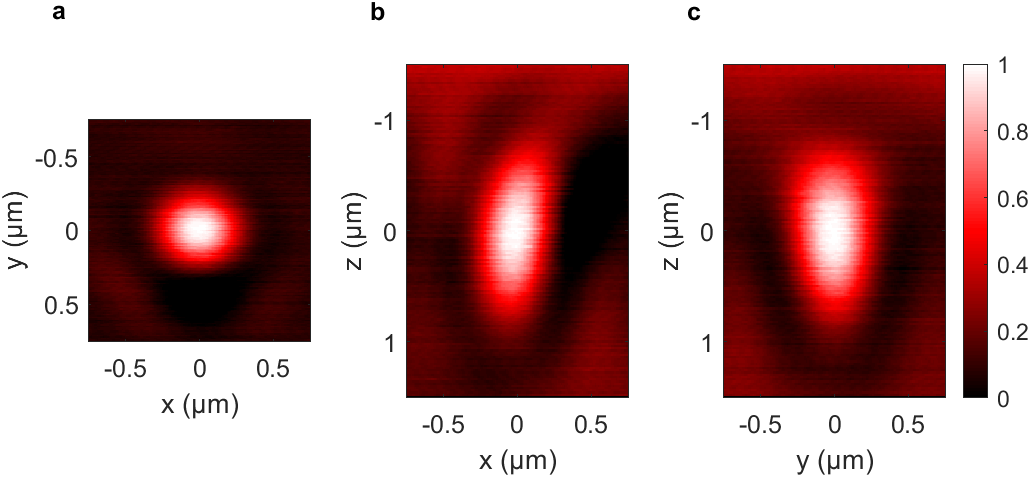


**Figure S7.** 780 nm laser beam profile as measured by scattering from a 200 nm gold bead. Data is normalized to the peak value. Each panel is a 2D slice in each of the planes **a** XY, **b** XZ, and **c** YZ.

# ZnO wire cross-section

By cutting focused ion beam (FIB) cross-sections of the ZnO wires, the inner structure can be examined (**Figure S8**). The SE-SEM image acquired at a 52° tilt angle reveals the cross-section of a ZnO wire. In ZnO, nm-sized pores are distributed within the ZnO film. A few larger pores are marked with arrows. A clear boundary between the substrate and ZnO is visible. The solid red line marks the border between the printed ZnO cross-section and sample surfaces coated with a Pt layer.


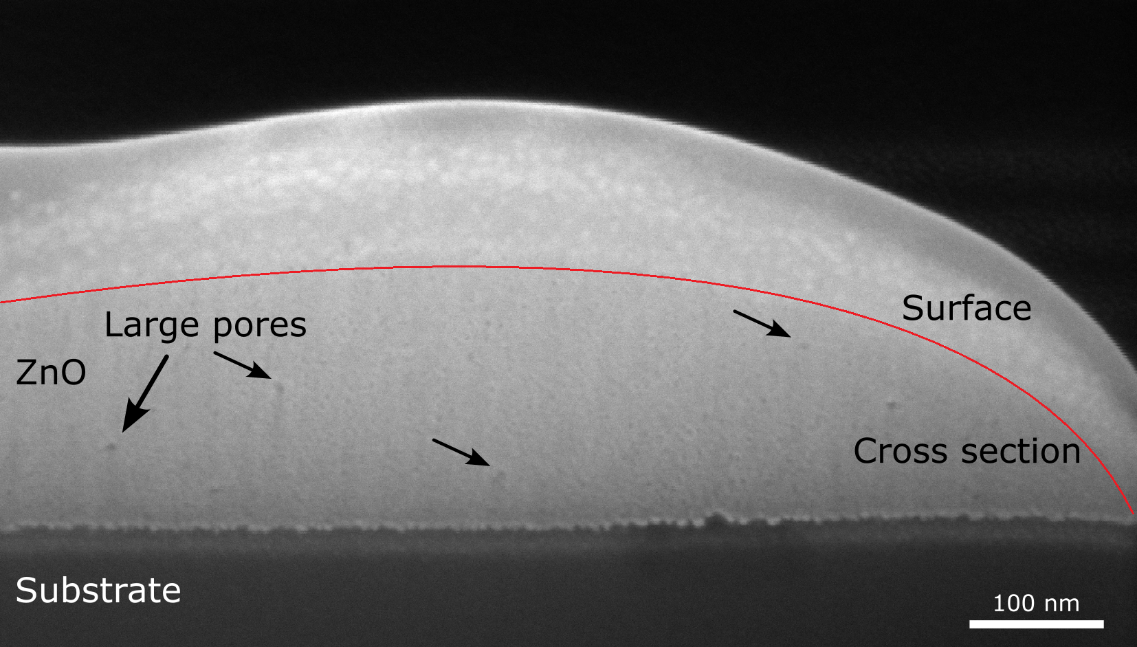


**Figure S8.** Tilted SE-SEM cross-section image of a 10 µm long ZnO wire printed at a laser power of 2 mW (2.25 MW cm^-2^) and laser scanning speed of 1 µm s^-1^ showing a porous ZnO microstructure. The pores have a diameter of a few nm and are barely resolved by SEM imaging. Large pores in the ZnO cross-section are indicated with the arrows. The red line separates the printed ZnO cross-section and the surface coated with a Pt layer.

# EBSD on different geometries

Secondary electron (SE) SEM top view images in **Figure S9a** and IPF maps in **Figure S9b-d** of one acute (60°) and two obtuse (120 and 150°) printed ZnO angles are shown. The printing direction is initially along the *x*-direction starting at the horizontal part and continues with the diagonal part after the bend. The IPF maps for a specific direction, e.g., IPF-Z, show three different colors at the start of the printing process. This shows the initial random orientation of ZnO. One single color gradient can be seen in the printing direction in the IPF maps of the 60 and 120° angles, which indicates that the crystal continuously changes its crystallographic orientation (left and middle column in **Figure S9**).

For the 150° angle, however, there is an additional abrupt color change in the horizontal part of the wire (marked with arrows in the IPF maps in the right column in **Figure S9**, especially visible in the IPF-X map). This indicates a grain boundary in the middle of the horizontal part of the 150° angle. Such an additional grain boundary in the primary orientation was only observed for 5 of 35 investigated printed wires (excluding the polycrystalline structure at the starting point of printing).

Another noticeable feature is that the color gradients of the different angles suggest varying rotation rates before and after the bend, that is, after changing the printing direction. In the horizontal part of the 60° angle in all 3 IPF maps, there is only a slight color change, which indicates that the crystal rotates minimally. In contrast, the 120° angle shows a more pronounced color gradient over the entire printed distance, indicating the crystal has a higher rotation rate. The total scalar misorientations for the horizontal part of the 60 and 120° angle are 6.70 and 28.22°, respectively. For horizontal lengths of 9.22 and 9.45 µm, this corresponds to average rotation rates of 0.73 and 2.99° µm^-1^, respectively. The horizontal part of the angle ranges from the starting point of the printing until the bend and the diagonal part from the bend to the end of the angle. After the bend, the rotation rates are 3.36° µm^-1^ for the 60° angle with 8.20 µm diagonal length and 4.77° µm^-1^ for the 120° angle with 9.18 µm diagonal length. As a result, the rotation rates after bending have increased and likely depend on the printing direction, which is similarly observed for the angles shown in the main text. This aspect is less prominent (and hence was not measured here) for the 150° angle due to the presence of a grain boundary in the horizontal part.


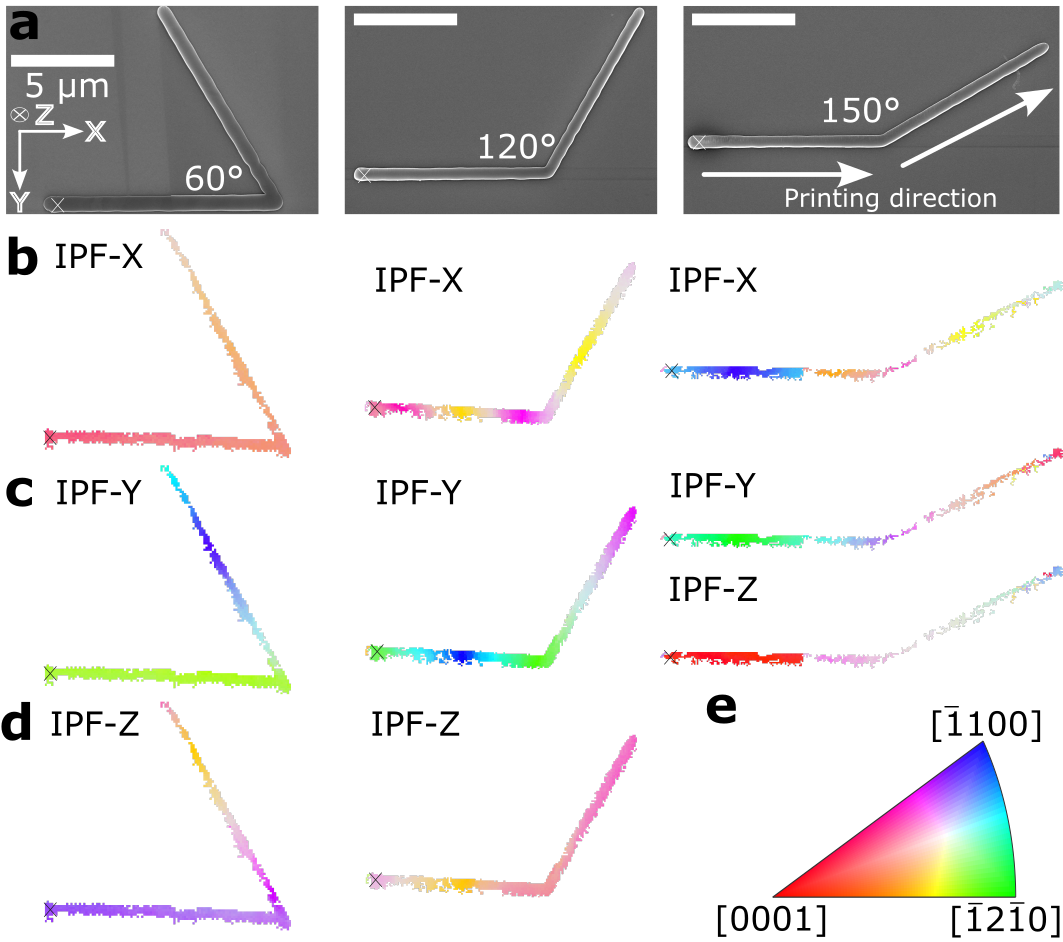


**Figure S9.** **a** Top-view SE-SEM images of different angles (60, 120, and 150°) printed at a laser power of 2 mW (2.25 MW cm^-2^) and laser scanning speed of 1 µm s^-1^. The printing start position at the lower left side in all cases and is markes with a cross. **b-d** IPF maps of the three angles. **e** Color legend for the IPF maps.

In addition, the misorientation values along the printing direction of the 10 µm ZnO wire (**Figure 2** in the main text) and the 120° angle (**Figure S9**) were analyzed and plotted (**Figure S10**). In **Figure S10a**, dashed line in the IPF-X maps indicate where the misorientation was calculated. The start and end positions are marked and correspond to the printing direction. For the 10 µm wire, the printing direction is in the *-x* real-space direction (and *vice versa* for the angle). The corresponding plot (**Figure S10b**) illustrates a linear increase of the misorientation, indicative of a constant rotation rate.

The 120° angle was split into its horizontal and diagonal parts. Here, the printing direction is in positive *x* real-space direction (and diagonal after the bend). The plot in **Figure S10b** for the horizontal part reveals a non-linear misorientation change. From 0 to 6 µm the misorientation first increases toward a maximum misorientation. Then, from 6 µm on, the crystal misorientation reduces and forms a local minimum around 7 µm. The reasons for this behavior in this case are not clear, but it is possible that the crystal reached an unfavorable orientation around 6 µm and gradually rotated along another direction which has a smaller overall misorientation relative to the reference point at 0 µm.

Notably, this behavior of a non-linear misorientation rate is an exceptional case like those where the rotation rate saturates (e.g., 5 µm circle in **Figure S11**). The diagonal part of the angle (right IPF-X map) shows an increasing misorientation with a more linear rotation rate. The distance at which the diagonal part starts corresponds to the position where the horizontal part ends. It is evident from the two plots for the 120° angle that the misorientation, and consequently the rotation rate, is influenced by the printing direction.


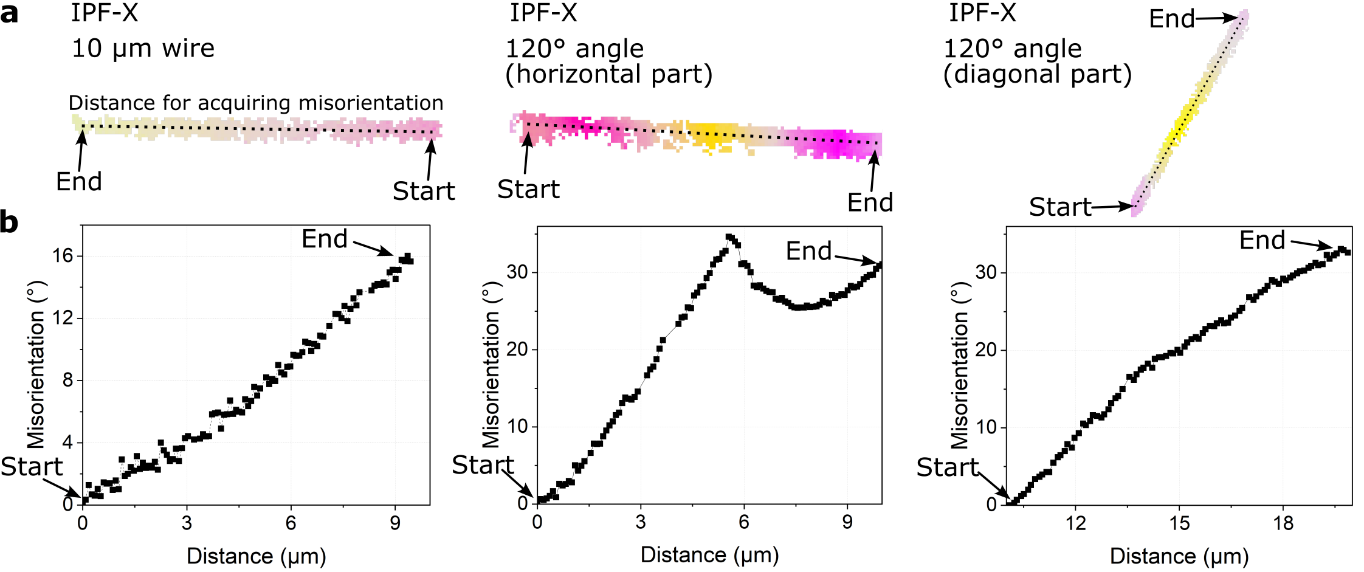


**Figure S10.** **a** IPF-X map of 10 µm ZnO wire (from **Figure 2**), horizontal, and diagonal part of 120° angle (from **Figure S9**). The dashed lines indicate the distance where misorientation values are calculated. The start and end position of the misorientation are shown and correspond to the printing direction. **b** Misorientation graphs over the dashed lines from **(a)** with corresponding start and end position. The start position of the diagonal part of the 120° angle corresponds to the end position of the horizontal part.

In addition to the angles, EBSD measurements were also performed on circles with larger radii than shown in the main text (1 µm). In **Figure S11**, SE-SEM images (**Figure S11a**) and IPF maps (**Figure S11b-d**) of representative circles with 2.5 and 5 µm radius are shown. The circles are printed clockwise starting at the 12 o'clock position. The IPF maps of both circles have in common that roughly the first quarter to a third of the circle are *not* single crystalline but instead show small grains. This can be recognized by the small regions with a different crystallographic orientation at the beginning of the printed circles in the IPF maps (**Figure S11b-d**). Similar to the straight wires and angles, a primary ZnO orientation may be selected from a polycrystalline seed upon printing. In the progression of the circles, single crystals (i.e., a single main IPF map color) with a color gradient are shown, indicating rotating ZnO single crystals. The initial crystallographic orientation is different for both circles since the ZnO crystals at the beginning are aligned in a random crystallographic orientation and a different primary orientation grows upon printing.

In addition to the IPF maps, misorientation maps are shown (**Figure S11e**). Two reference points are marked with solid and dashed arrows in the misorientation maps. The solid arrow marks the beginning of the ZnO single crystal between 1 and 3 o'clock (marked with a solid arrow in **Figure S11e**). The dashed arrow close to the 12 o’clock position marks the endpoint (coinciding with the maximum misorientation value). Remarkable is the misorientation of the circle with a radius of 5.0 µm. Here, the misorientation increases from the beginning of the ZnO single crystal at 1 o'clock to approximately 7 o'clock. From 7 to 12 o'clock, the misorientation is constant, recognizable by the non-changing color gradient in the misorientation plot. This indicates a non-linear rotation rate (i.e., no change at all in this region). In contrast, for the 2.5 µm circle, the misorientation increases along the entire circumference (upper plot in **Figure S11e**).

The total scalar misorientations are (15 and 29°) resulting in average rotation rates (1.25, 0.99° µm^-1^) for the shown circles with radii (2.5 and 5.0 µm). The values for the mean rotation rate of the circle with 5 µm radius must be carefully interpreted, as the rotation rate is not linear. The “effective” rotation rate for 5 µm radius between the 1 and 7 o’clock positions is 1.81° µm^­1^.


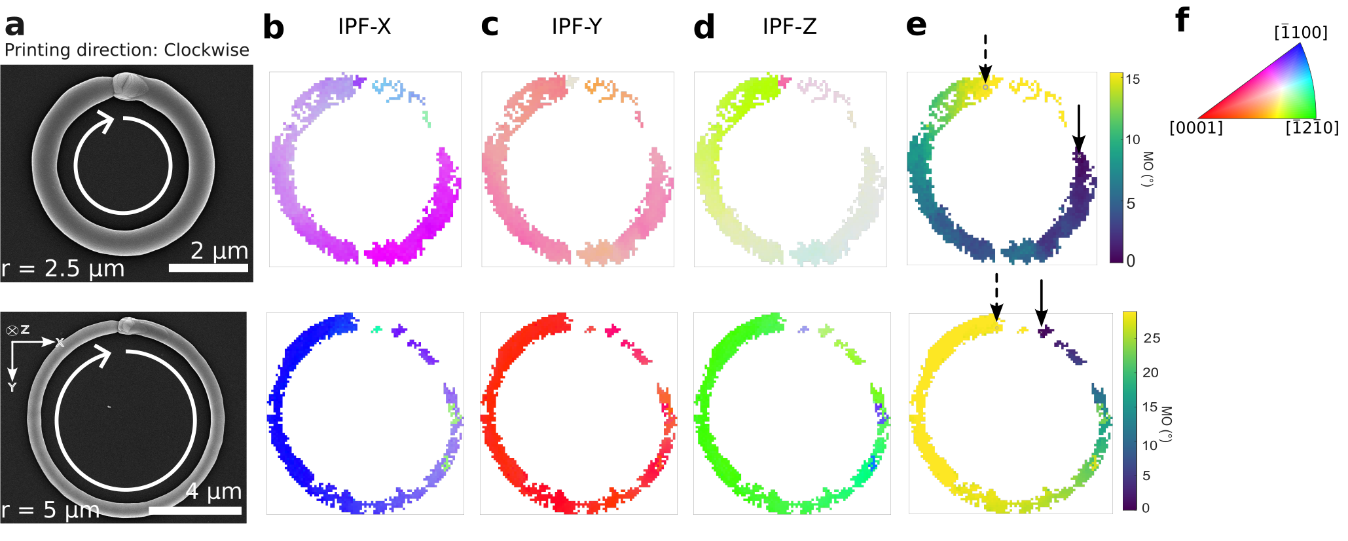


**Figure S11**. **a** Top-view SE-SEM image of circles (printed at 2 mW (2.25 MW cm^-2^) and 1 µm s^-1^) with 2.5 and 5 µm radius with clockwise printing direction starting at the top. **b-d** Corresponding IPF maps. **e** Misorientation map with reference point at approximately 3 and 1 o’clock (marked with solid arrows). Dashed arrows mark the endpoints. **f** Color legend for the IPF maps.

# Texture analysis of all investigated straight wires

The EBSD data from all investigated ZnO structures containing straight wires in the horizontal direction *x* are analyzed in this section. This includes single, straight wire but also the horizontal parts of the angles. These parts were chosen since they are printed under the same conditions, that is, the laser writing starts at a pristine spot without any ZnO and moves straight. In contrast, the diagonal parts after the bend for angles are also straight, but the “starting position” of these wires is after a bend from another straight ZnO wire. As a result, the initial printing conditions are not the same (different heat profile and predefined texture from the preceding ZnO) as from a pristine spot on the substrate. In total, 24 straight ZnO wires are shown in the following. The printing direction for the investigated wires is either in the *+x* or *-x*‑direction. Only 150 to 180 random orientations are plotted for each wire for brevity, which is enough to indicate the tendency of lattice rotations.

**Figure S12** presents all orientations in the [0001] pole figure (PF). Each color represents a different ZnO wire. The black markers (⋆ for 1 and × for 5 µm s^-1^ printing speed) indicate the initial reference orientation after the formation of the primary ZnO orientation close to the starting point of the printing. The continuous change in ZnO orientation is visible as continuous lines in the PF. The wire with a rotation rate of 1.69 ° µm^-1^ in the top-left region in **Figure S9** corresponds to the ZnO wire shown in **Figure 2** in the main text. The numbers close to the marker denote the average rotation rate in ° µm^-1^. Regarding the latter, the average rotation rate for all wires and different printing speeds are (3.13 ± 0.34) ° µm^-1^ for 1 µm s^-1^ printing speed and (1.60 ± 0.25) ° µm^-1^ for 5 µm s^-1^ printing speed. The errors denote the standard errors for *N* = 21 and *N* = 3 wires for 1 and 5 µm s^-1^, respectively. The standard deviations are 1.56 and 0.42 ° µm^-1^. Even though only three values were available for the higher printing speeds, a higher average rotation rate for slower crystal growth rates was similarly observed in other bending crystal lattices^[12]^ but may also be related to the reduced ZnO height for higher printing speeds (**Figure S5b**).^[13]^

The spread of starting points in the PF in **Figure S12** exemplifies again that the starting orientations of ZnO are likely random on the amorphous substrate. Nucleation (or formation of the primary ZnO orientation) close to the *z‑*direction in some cases leads to a tendency for an alignment of [0001]||*z* (solid arrows in **Figure S12** mark four examples). However, the opposite trend can also be observed (dotted arrows in **Figure S12** mark two examples), so that overall no clear tendency toward [0001]||*z* alignment is observed.

For completeness, the (inverse) PFs of the other main crystallographic directions of the hexagonal wurtzite structure are shown in **Figure S13** to **Figure S16**. Notably, the chosen IPF directions are *z* (surface normal, **Figure S15**) and *x* (printing direction, same as *-x*, **Figure S16**). The IPF in *z*‑direction (**Figure S15**) shows similar trends as the [0001]-PF in **Figure S12**, where some wires tend toward the [0001] pole (i.e., [0001]||*z* alignment), while others “move” away from it.


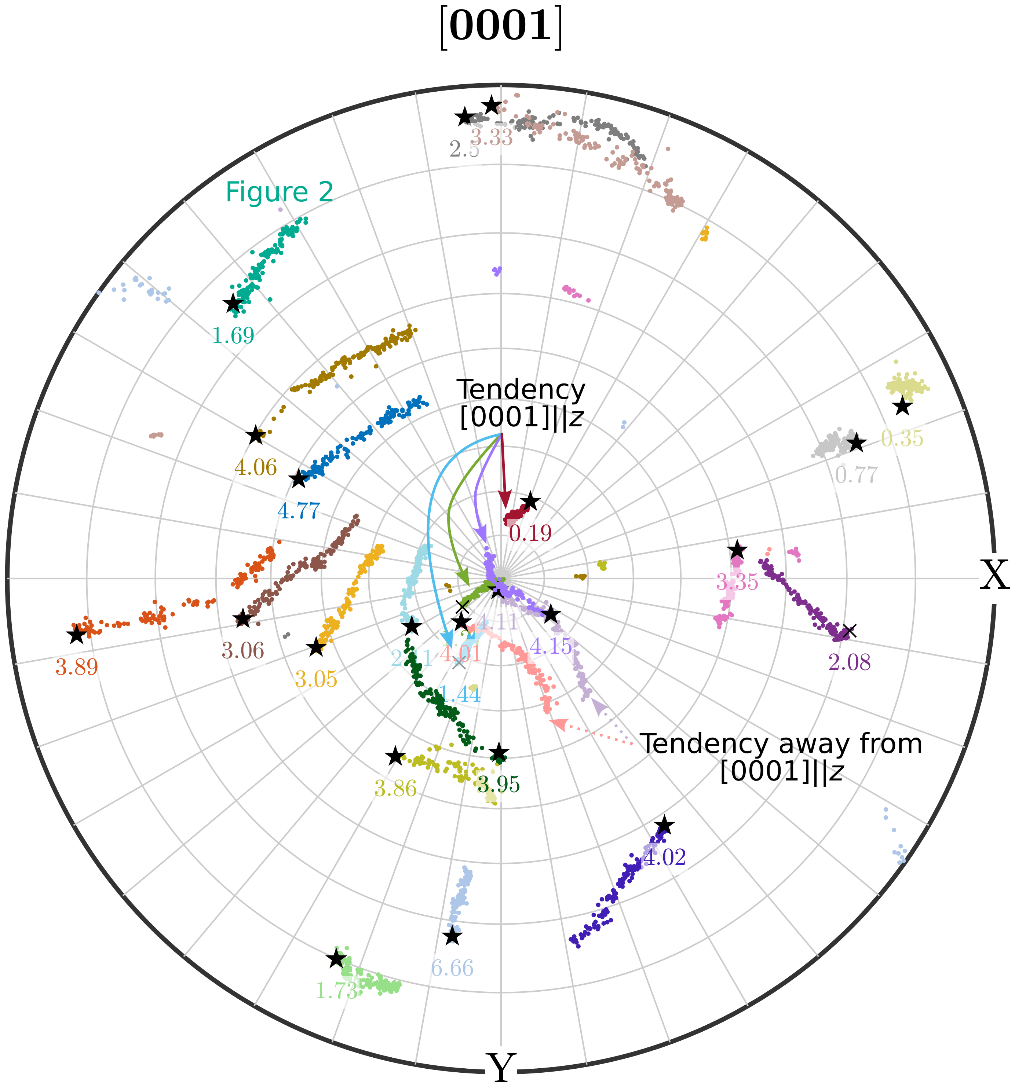


**Figure S12.** Pole figure for the [0001] direction for all investigated straight ZnO wires. The markers indicate the starting orientation of ZnO after the formation of the primary ZnO orientation in the printing process. The numbers indicate the average rotation rate in ° µm^-1^. Four wires starting close to a [0001]||z show a tendency toward [0001]||z (solid arrows), whereas two wires show an opposite tendency (dotted arrows).


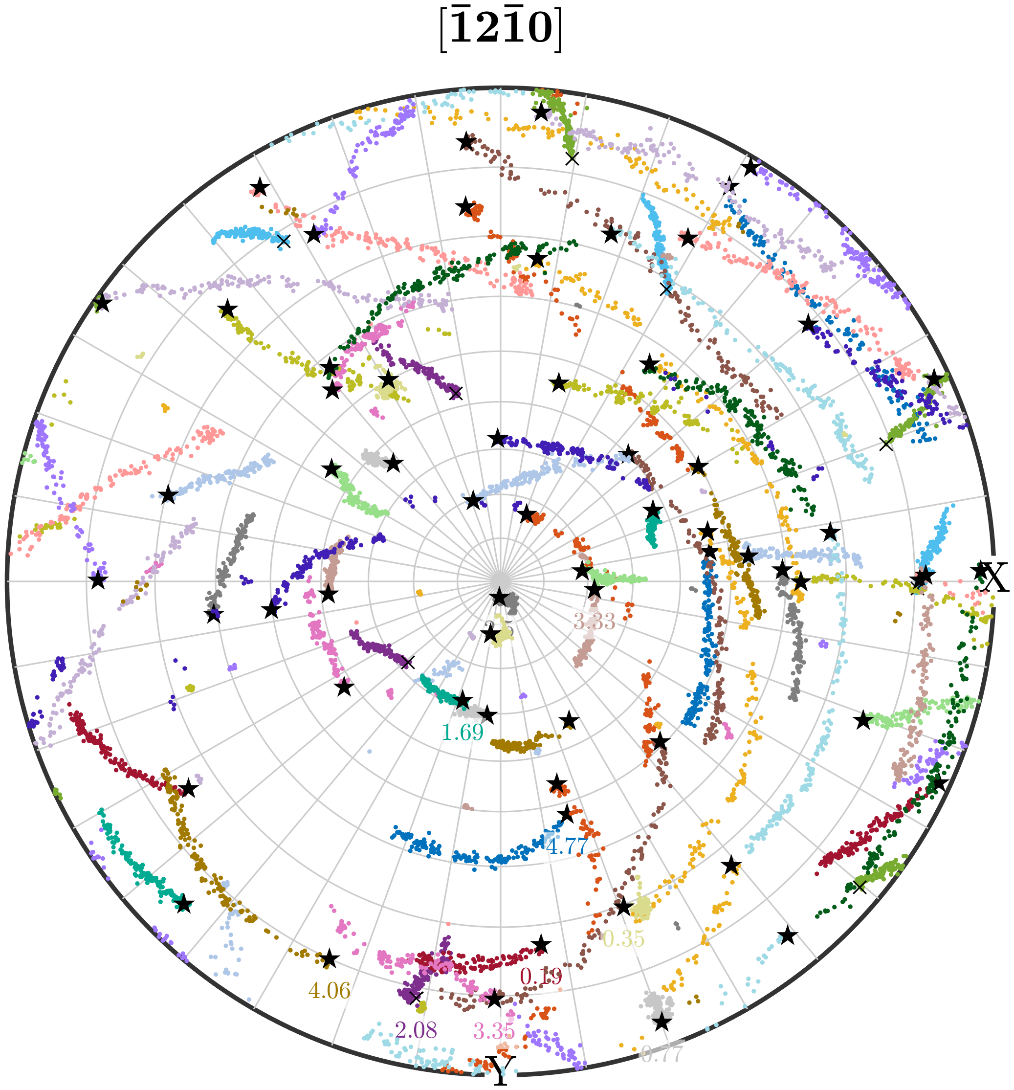


**Figure S13.** Pole figure for the [20] direction for all investigated straight ZnO wires. The markers indicate the starting orientation of ZnO after the formation of the primary ZnO orientation in the printing process. The numbers indicate the average rotation rate in ° µm^-1^.


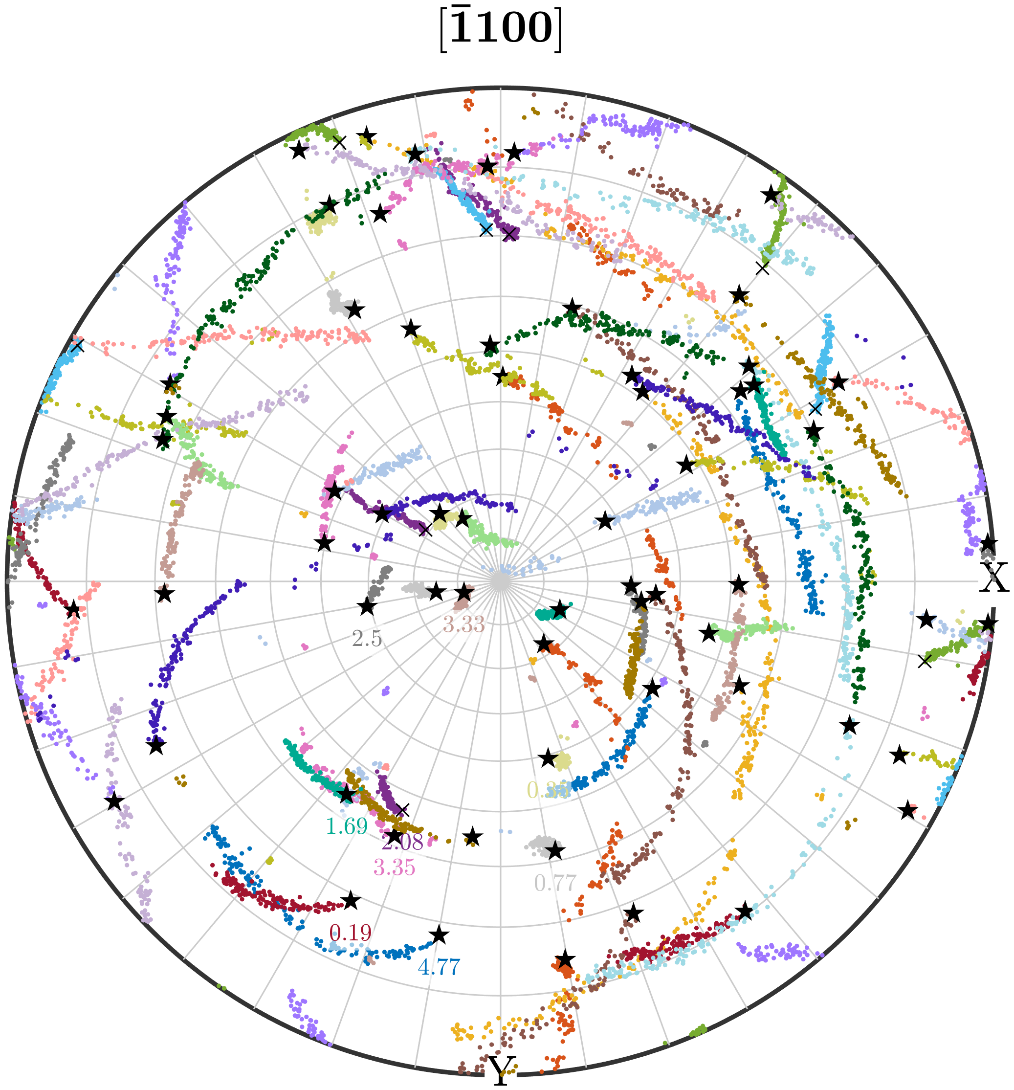


**Figure S14.** Pole figure for the [100] direction for all investigated straight ZnO wires. The markers indicate the starting orientation of ZnO after the formation of the primary ZnO orientation in the printing process. The numbers indicate the average rotation rate in ° µm^-1^.


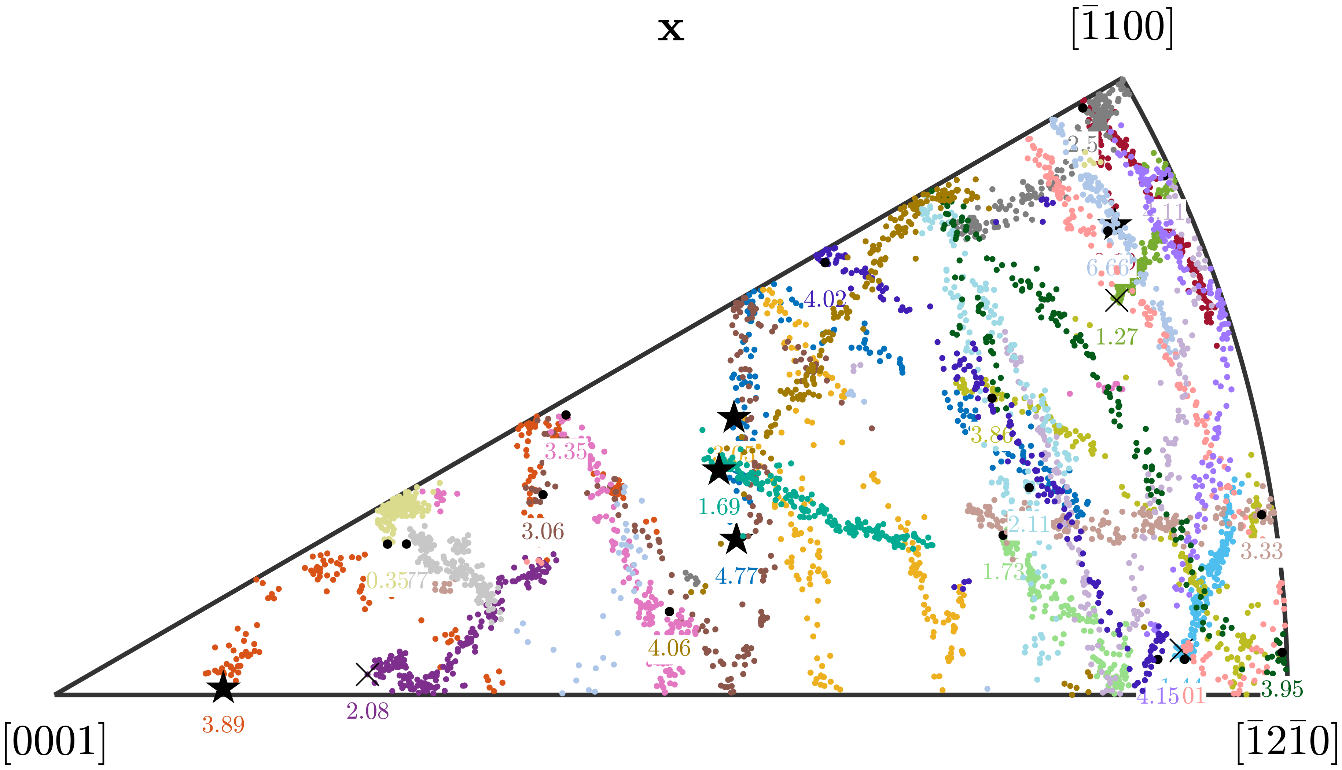


**Figure S15.** Inverse pole figure for the x‑direction corresponding to the printing direction. for all investigated straight ZnO wires. The markers indicate the starting orientation of ZnO after the formation of the primary ZnO orientation in the printing process. The numbers indicate the average rotation rate in ° µm^-1^.


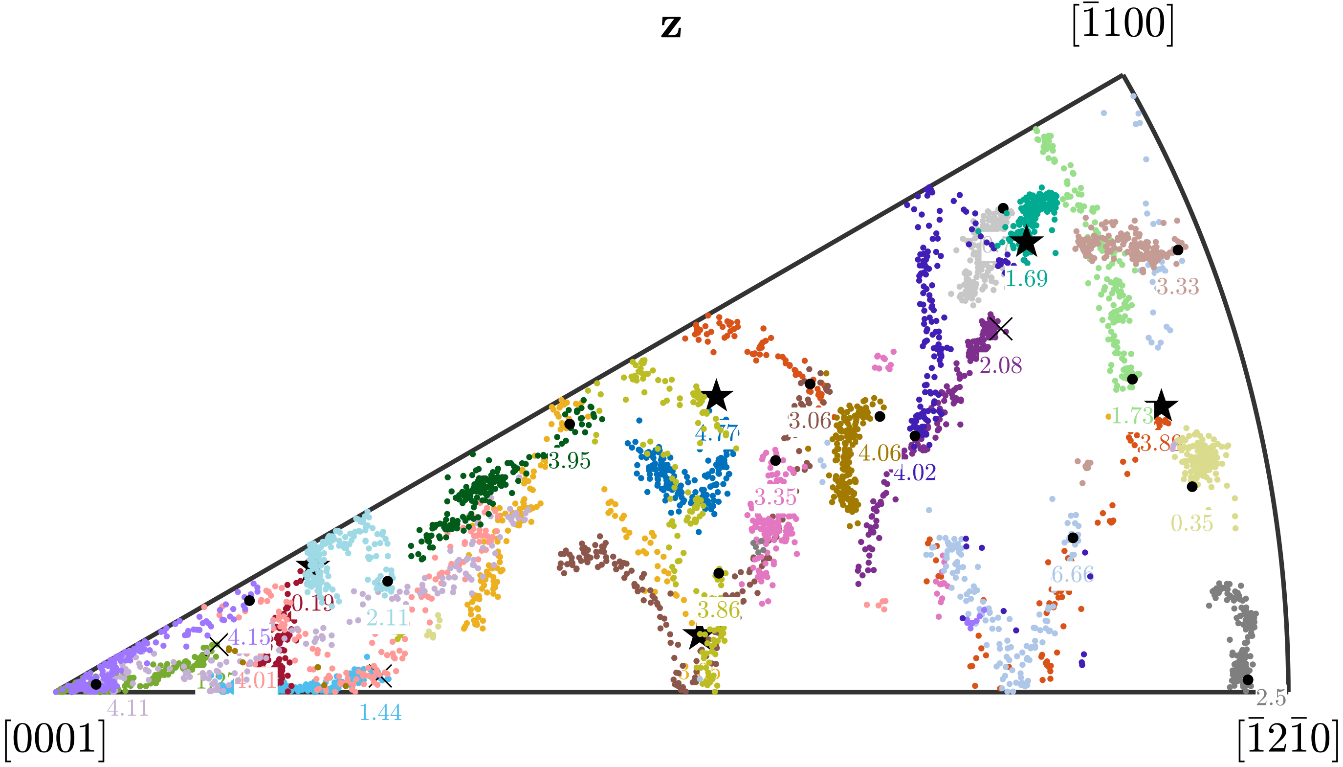


**Figure S16.** Inverse pole figure for the z‑direction corresponding surface normal (but antiparallel, pointing into the sample) for all investigated straight ZnO wires. The markers indicate the starting orientation of ZnO after the formation of the primary ZnO orientation in the printing process. The numbers indicate the average rotation rate in ° µm^-1^.

**Figure S17** presents pole figures (PFs) for the lattice planes that are initially (i.e., at the reference orientation at the start of the printing or after the formation of the primary ZnO orientation from a polycrystalline seed) parallel to the surface, i.e., their plane normal is parallel to *z*. Thus, the starting orientation is aligned at the *z*‑direction, which is in the center of the shown PFs. The Miller-Bravais indices (*hkil*) for each such plane for each ZnO wire are given in the PF titles. Note that *i* = -(*h*+*k*) for interpreting the indices. For example, one PF in the last row corresponds to the data shown in **Figure 2** in the main text. Here, (*hkil*) = (115) corresponds to *h* = , *k* = 11, *i* = 0, *l* = 5. The printing direction (PD) is marked in each PF and varies between *x* and *-x*. The value in the center of the PFs denotes the average rotation rate in ° µm^-1^. The PF directions were calculated from the reference orientations using the *round2Miller* function in MTEX version 5.10.2^[14]^ and slight deviations of the starting point from the center position of the PFs (*z*-direction) result from rounding of the Miller-Bravais indices.


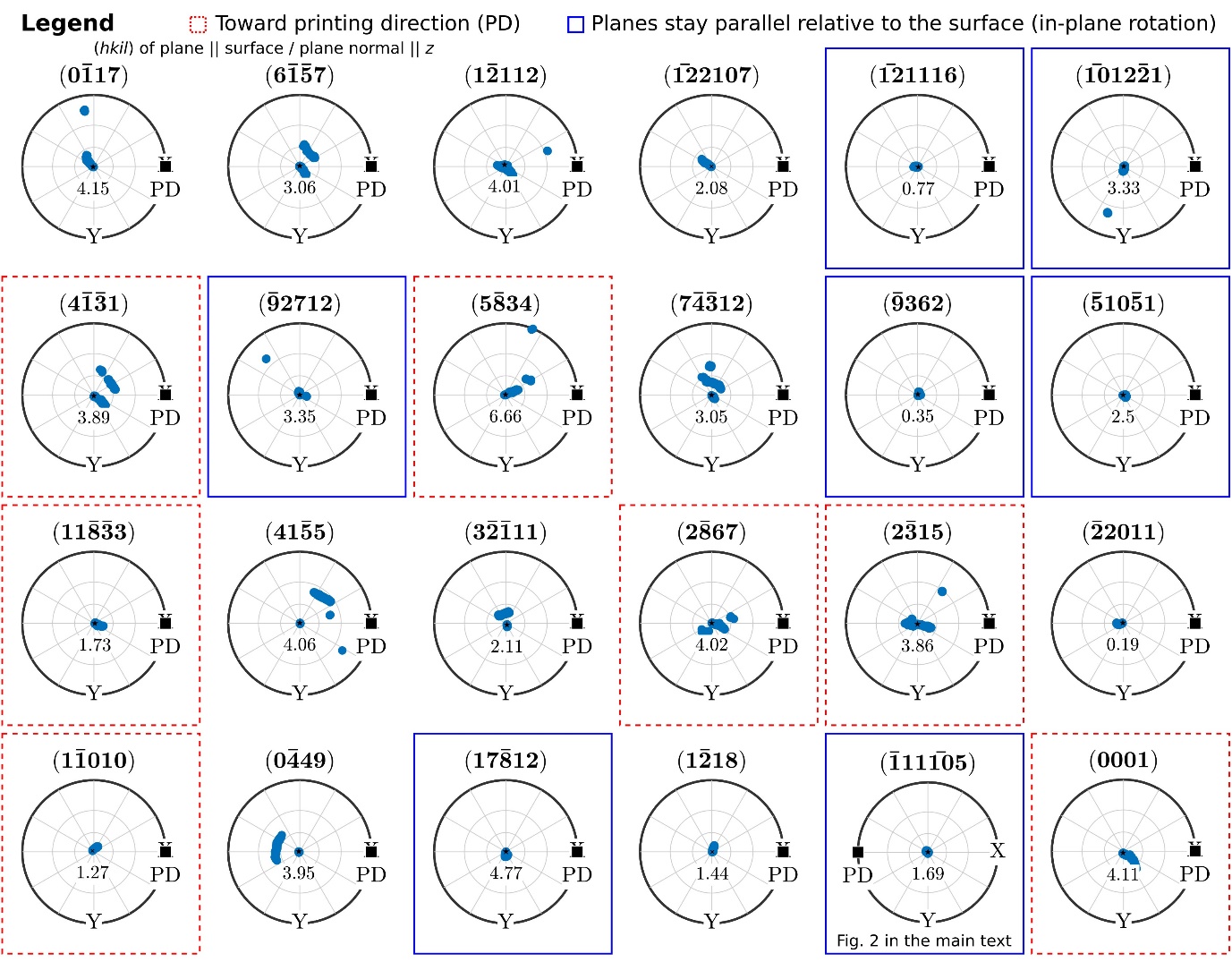


**Figure S17.** Pole figures (PFs) for the lattice planes initially aligned parallel to the surface at the start of the printing process (with plane normal || z). The titles show the Miller-Bravais indices (hkil) of these lattice planes. The printing direction (PD) is either in the x or -x‑direction and denoted in the PFs. PFs marked with dashed red frames show a tendency of lattice planes “rolling off” along PD. PFs marked with a solid blue frame show no or only a slight change in orientation of the lattice plane relative to the z-direction, indicating an in-plane rotation of ZnO around the z-direction. Unmarked PFs show the rotation of the lattice planes in other directions than PD. Numbers in the PFs denote the average rotation rates in ° µm^-1^.

The rotations of the shown lattice planes upon printing are subjectively classified into different categories by visual inspection. Firstly, the PFs marked with a solid blue frame show no or only slight variations of lattice orientations away from the *z*-direction. In these cases, the rotation is essentially an in-plane rotation of the ZnO crystal around the *z*-direction. The example ZnO wire shown in **Figure 2** in the main text falls into this category (cf. last row in **Figure S14**). Secondly, PFs marked with a dashed red frame show a tendency of the lattice planes to “roll off” along the PD. This effect was observed in other works with similar rotating crystal structures which were, however, produced with different techniques.^[13,15]^ Thirdly, the unmarked PFs show rotations in various directions that are *not* along the PD. For example, the first PF in the top-left corner shows a rotation of the lattice plane away from *z*toward the ‑*y‑*direction (perpendicular to PD). Sudden discontinuities in the PFs may be caused either by grain boundaries or symmetrically equivalent planes.

Overall, based on the differences of 24 shown PFs, no clear trend for the alignment and rotation of the initially surface-parallel lattice planes can be observed, which is in contrast to similar structures with a more predictable “roll-off” behavior toward the PD.^[13,15]^ Also, the bottom right corner shows an example where the (0001) planes are initially aligned parallel to the surface plane. Upon printing, the ZnO crystal rotates away (with a slight tendency toward PD) from this highly symmetrical configuration even in this “ideal” case of a (0001)-oriented texture of ZnO.^[16]^

# Electron Backscatter Diffraction Analysis of a 100 µm long wire

A few 100 µm long ZnO wires were printed with a laser scanning speed of 1 µm s^-1^ and a laser power of 2.5 mW (2.815 MW cm^-2^). Representative results for one wire are shown in the following. The crystal orientation and rotation were analyzed by EBSD as described in the main text. Three separate measurements of the one long wire were recorded and combined in post-processing to cover the relatively large area with sufficient real-space sampling with the electron probe. The printing direction is shown in **Figure S18a** in the positive real-space direction *x*. A single primary color is observed in the inverse pole figure (IPF) maps (**Figure S18b-d**), which indicates a single ZnO grain (or ZnO single crystal). In addition, a smooth color gradient for the single crystal can be seen for each IPF map. This indicates that the shown 100 µm long wire has a lattice rotation. The latter is also seen based on the different orientations of the schematic hexagonal ZnO unit cells at the beginning (**Figure S18e**) and at the end of the printed wire (**Figure S18j**).

With the help of the pole figures (**Figure S18f-h**), it can be seen in which direction the ZnO crystal rotates. The pole-figure colors show the scalar misorientation between a reference point at the start and the end of the printing (marked in the IPF-Z map in **Figure S18d**). The misorientation values are given in the color bar in **Figure S18h**. For this wire, the [0001] direction is initially closely aligned with the *z‑*direction, i.e., the surface normal (**Figure S18e** and **f**). Upon printing, the [0001] crystal direction aligns even more with the *z*‑direction (**Figure S18f** and **j**) and the crystal rotates clockwise around the *z*‑direction/[0001] axis. The latter aspect can be seen in the [20] and [100] pole figures (**Figure S18g** and **h**). Similar to the 10 µm ZnO wire in the main text (**Figure 2** in the main text), this ZnO crystal essentially rotates around the *z*‑direction. However, the crystal direction initially aligned close to *z* between the two wires differs, i.e., [100] for the 10 µm wire (**Figure 2j** in the main text) and [0001] for the 100 µm wire (**Figure S18f**). This again shows that the (i) initial orientation and (ii) the forming primary orientation differ between printed wires on the amorphous substrate. Also, the crystal rotation is not always around the *z*‑direction (**Figure S17**). The total scalar misorientation is 18.7° and the rotation rate is 0.19° µm^-1^ for the 100 µm wire. The latter is relatively small compared to the other investigated wires.


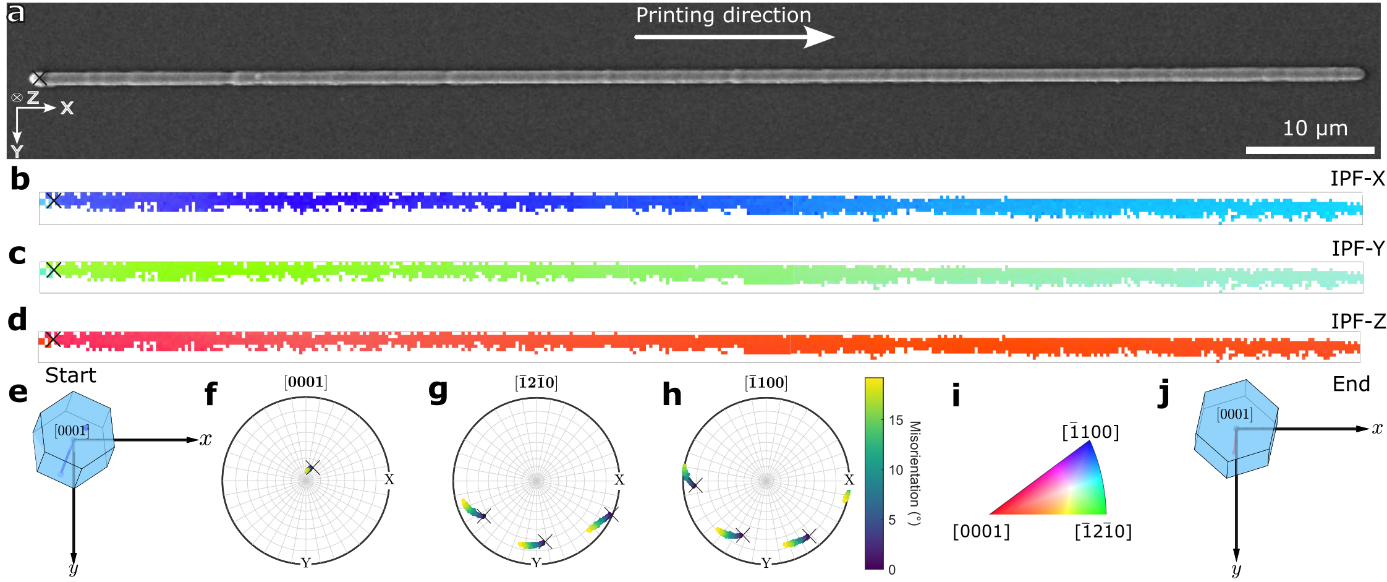


**Figure S18. a** Top-view SE-SEM image of a 100 µm long ZnO wire with marked printing direction. **b-d** Colored inverse pole figure (IPF) maps merged from three individual EBSD measurements. **e** Schematic representation and orientation of hexagonal lattice at the beginning and (**j**) at the end of the printed ZnO wire. **f-h** Pole figures for the [0001], [100], and [20] directions. The color bar in **h** shows the scalar misorientation relative to the selected start point in the IPF-Z map up to the endpoint. **i** Color legend for IPF maps of ZnO.

# Chemical Analysis by Energy-dispersive X-ray Spectroscopy (EDXS)

STEM-EDXS was used to investigate the chemical composition of two printed ZnO wires and the substrate (**Figure S19**). Cross-section TEM samples were prepared from (i) a ZnO wire previously measured by EBSD (**Figure S19a**) and (ii) an as-printed wire, which was not modified after laser printing (**Figure S19b**). The magnification for STEM-EDXS was chosen to cover all relevant deposited layers, i.e., glass coverslip substrate, Ar^+^-sputtered Si (measured 265 nm), and amorphous SiO_2_ (measured ~18 nm), and laser-printed ZnO (here about 175 nm and 270 nm, **Figure S19a** and **b**, respectively). Note that for EBSD measurements the ZnO wire was polished by FIB which removes part of the ZnO from the surface and leads to a reduced ZnO layer thickness (175 nm) compared to the as-printed wire (270 nm) for the same printing parameters (cf. HAADF-STEM images in **Figure S19**). It is likely that around 100 nm ZnO was removed from the EBSD-measured ZnO wire by FIB polishing. The FIB polishing of the surface for EBSD leads to a homogeneous ZnO layer thickness (or flat surface), whereas the as-printed wire shows slight thickness variations. In addition, the two samples may not be cut at the same lateral position of the wires during TEM-sample preparation, which can also lead to the difference in ZnO layer thicknesses observed by TEM.

The different elemental maps for (top rows) Zn, O, Si, Pt, and (bottom rows) C, S, Ar, Ti, and Ga are shown and mainly reveal the different layer compositions and qualitatively confirm the expected element distributions. Zn is only observed in ZnO, whereas O is predominantly observed in ZnO, SiO_2_, and the glass coverslip (which consists mainly of SiO_2_) in varying concentrations. The Zn and O signals are reduced at the pores (cf. Zn and O maps with the HAADF-STEM signal), indicating that the pores do not contain Zn and O (or have reduced Zn and O concentrations). Si is visible in the SiO_2_ layers and the Si layer. Pt and C signal on top of the ZnO film results from a protective, FIB-deposited Pt/C mixture that protects the ZnO.film during TEM sample preparation.


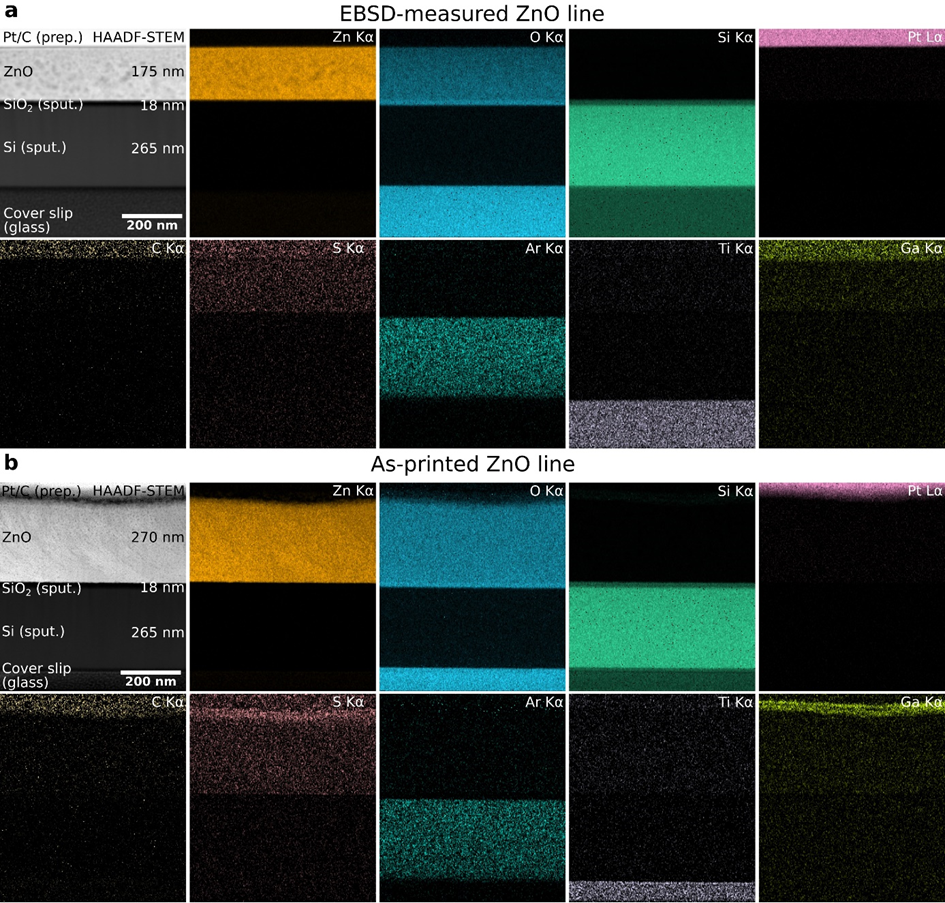


**Figure S19.** Chemical analysis of the layer composition by STEM-EDXS for **a** EBSD-measured and **b** as-printed ZnO wires. The upper (lower) rows show the elements with high (low) concentrations. The ZnO layer consists mainly of Zn and O with minor signals of S and C.

Elemental maps for elements with minor concentrations (< 3 at%) are also shown in **Figure S19**. Ar is observed in the Ar^+^-ion-sputtered, thicker Si layer, but not in the thinner SiO_2_ layer. The Ti map is displayed to show the glass-coverslip region, where Ti is a minor element in the used glass. The Ga signal results from Ga^+^-ion FIB preparation. We suspected a pronounced Ga signal on the surface of ZnO for the EBSD-measured wire since Ga is typically implanted into the first few nm by FIB polishing (here for EBSD measurements). However, we find such a Ga-rich layer only for the as-printed sample instead of the EBSD-measured sample. The Ga-rich layer for the as-printed sample may be explained by unintended Ga-implantation during FIB imaging during sample navigation for FIB-based EBSD polishing and/or TEM sample preparation. Ga bombardment will lead to the observed Ga implantation and possible amorphization of the ZnO surface. Besides Ga, an increased S signal is observed at the Ga-rich surface layer for the as-printed sample. Residual S in the ZnO film prepared from DMSO-based solution might preferentially diffuse into the partly amorphized ZnO surface.

The shown elemental maps correspond to background-corrected X-ray peak intensities (net intensities) and the displayed intensity range was adjusted for good visibility for each element. Quantitative wire profiles along the vertical direction starting from the protective Pt/C layer and ending in the glass-coverslip region were extracted from the STEM-EDXS data to get a better understanding of the layers’ chemical compositions (see **Figure S20** for the EBSD-measured wire and **Figure S21** for the as-printed wire). For the wire profiles, a higher signal-to-noise ratio than the elemental maps is achieved by integrating all spectra along the horizontal direction, which is beneficial for analyzing elements with minor concentrations. The top row shows the wire profiles with on a scale from 0 at% to 100 at%, whereas the middle row reveals minor concentration with the ordinate axis ranging from 0 at% to 3 at%. The lower row shows the 90°-rotated, gamma-adjusted HAADF-STEM signal (relative to **Figure S19**).

Starting with ZnO layer, the EBSD-measured wire shows roughly 60 at% Zn and 40 at% O for the EBSD-measured wire (**Figure S20a**) and about 50 at% Zn and 50 at% O for the as-printed wire (**Figure S21a**). The large discrepancy in O concentration for the EBSD-measured wire (**Figure S20a**) from the nominal 50 at% Zn and O expected for stoichiometric ZnO results primarily from X-ray absorption of low-energy O Kα X-rays (525 eV) in the TEM sample on the way toward the EDXS detector. Absorption will lead to an underestimation of O (and also other light elements such as C) as observed here. Indeed, the TEM samples were prepared relatively thick (about 120 nm to 150 nm sample thickness) to keep the structural integrity during thinning of a relatively large electron-transparent region for SAED measurements (see **Figure S25**). The TEM sample of the EBSD-measured wire is likely thicker than the TEM sample from the as-printed wire, which explains the differences in O concentration between the two wire profiles (cf. **Figure S20a** and **Figure S21a**). A reduction in O concentration results in an increase in Zn concentration since standardless EDXS quantification normalized the concentration to 100 at% (and not to measured standards/analytical total). Absorption correction was not applied since the densities of the layers are unknown, especially for porous ZnO. Still, the Zn/O ratio is close to the expected value of 1 for the as-printed sample, fitting with the observed ZnO structure in high-resolution (S)TEM and SAED measurements.


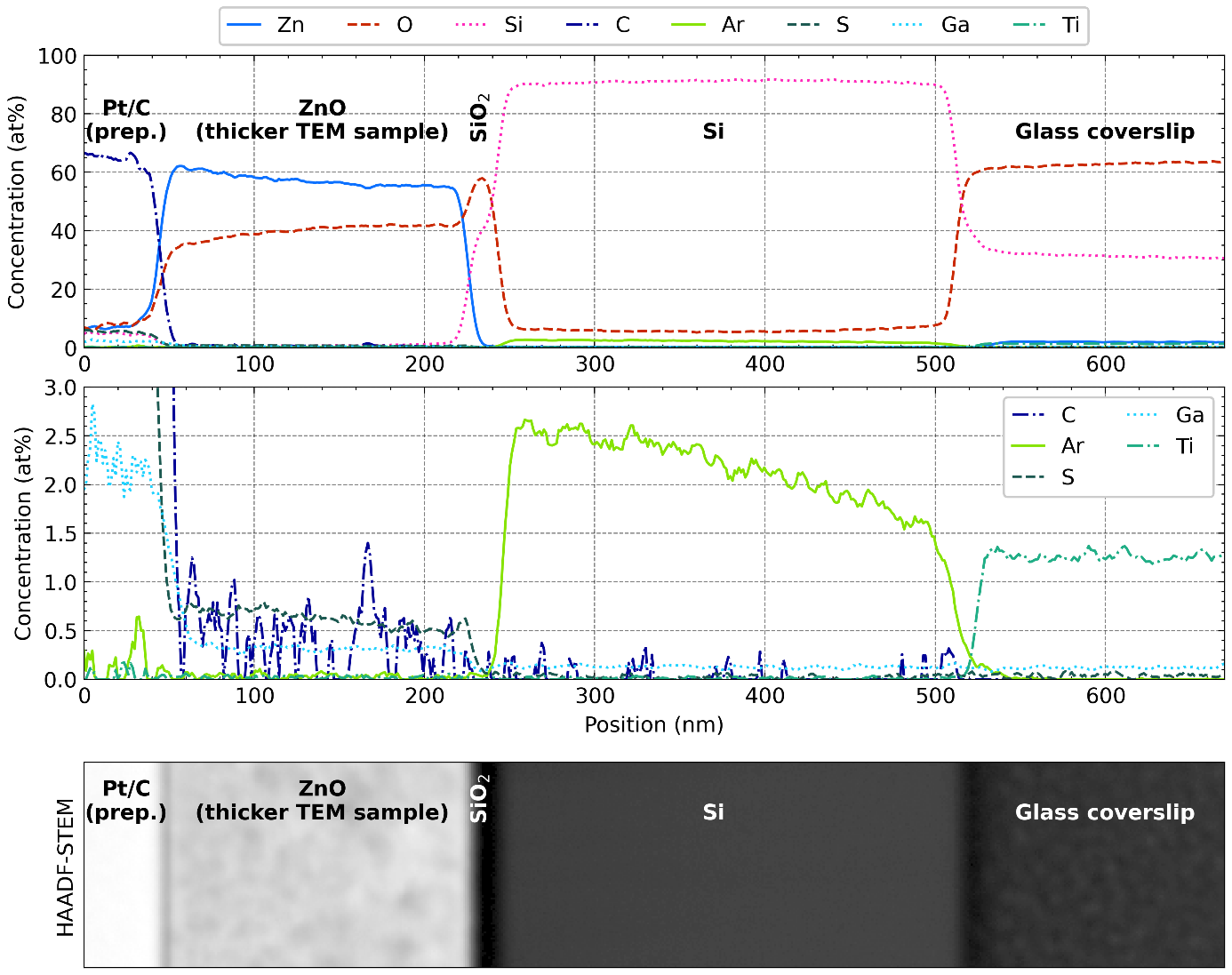


**Figure S20.** Wire profiles of quantified chemical compositions for the EBSD-measured ZnO wire. The top row shows the elemental concentrations with the ordinate scaled between 0 at% and 100 at%, whereas the middle row is scaled between 0 at% and 3 at% for the elements with minor concentrations. The bottom row shows the 90°-rotated HAADF-STEM signal. Note that this TEM sample was relatively thick, which leads to strong absorption effects and subsequent underestimation of low-Z elements such as C and O.


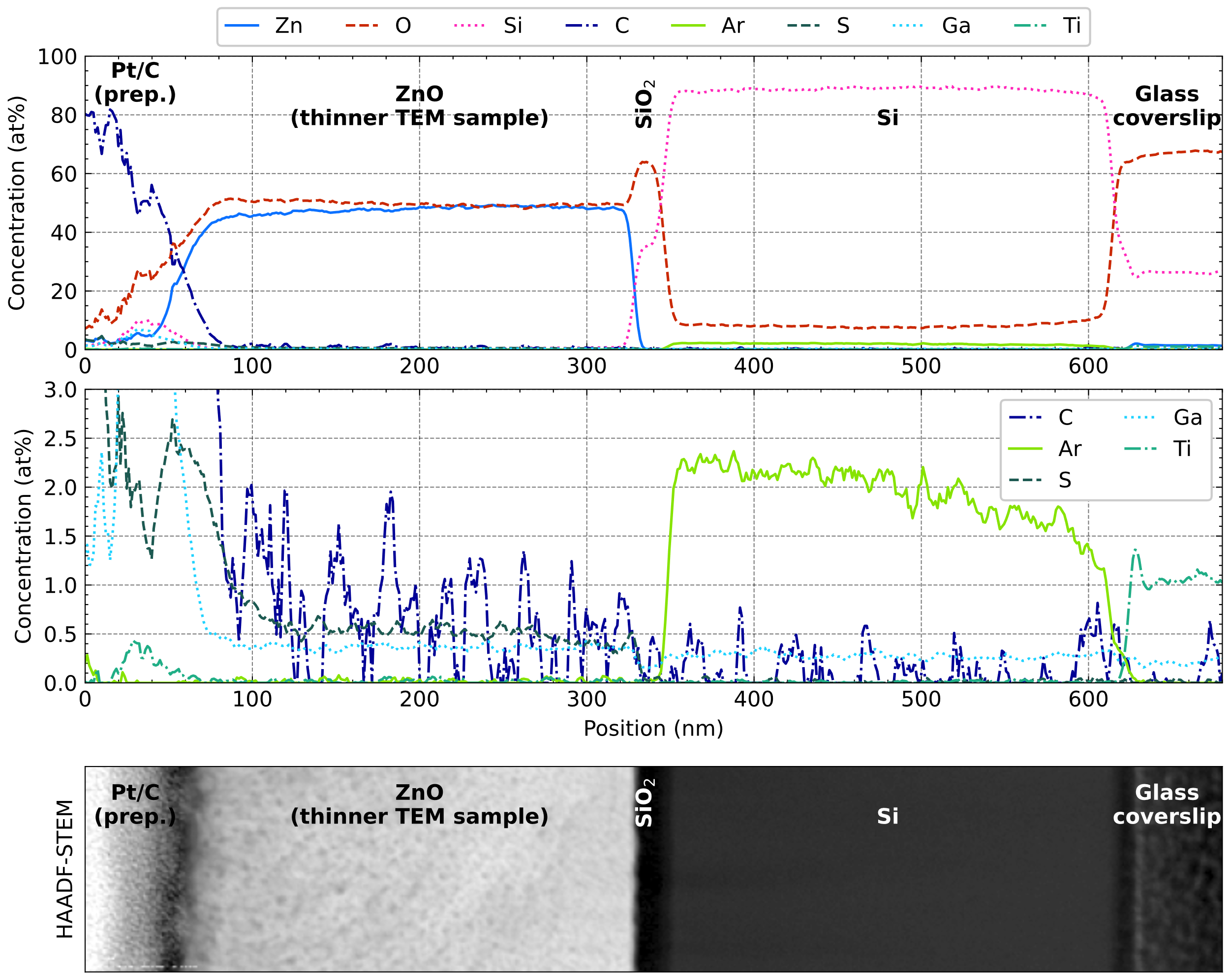


**Figure S21.** Wire profiles of quantified chemical compositions for the as-printed ZnO wire. The top row shows the elemental concentrations with the ordinate scaled between 0 at% and 100 at%, whereas the middle row is scaled between 0 at% and 3 at% for the elements with minor concentrations. The bottom row shows the 90°-rotated HAADF-STEM signal.

In both wire profiles, slight gradients in Zn/O signals are observed. These gradients may be caused by TEM-sample thickness variations but could also stem from genuine O-concentration differences in the ZnO layer from substrate to the surface. Oxygen vacancies are common in ZnO and can alter its properties,^[17]^ which would lead to a reduced O concentration measured by EDXS. However, this aspect requires careful standards-based EDXS measurements and could not be clarified based on our standardless EDXS quantification.

Besides Zn and O, small concentrations of S and C are observed in the ZnO layers (**Figure S20** and **Figure S21b**). Even though the C signal is quite noisy, a clear reduction in C signal is observed from the ZnO layer toward the substrate layers (SiO_2_ and Si), indicating that C is present in ZnO. S and C are present in DMSO (C_2_H_6_OS) and may (i) either be residual ink in the pores or (ii) be incorporated into the ZnO lattice. Regarding the pores, no significant change in S and C signals at the pores can be observed (in contrast to the Zn and O maps) in the elemental maps shown in **Figure S19**. This may be caused by a too low signal-to-noise ratio of minor concentrations of S and C in ZnO to spatially resolve small changes in their concentration around the pores for the given signal-to-noise ratio in the dataset. The S signal in the Pt/C protection layer may result from an overlap of the S Kα (2.31 keV) and Pt Mγ (2.33 keV) X-ray lines, resulting in a false S signal in the Pt-rich layer. A 5×5 pixel binning, convolution of neighboring spectra with a Gaussian kernel, and finally principal components analysis (PCA) was applied to denoise the STEM-EDXS dataset and to investigate the spatial distributions of S and C (**Figure S22a**). The local reduction of Zn, O, and S correlates with the reduced HAADF-STEM intensity at the pores. This suggests that S is incorporated in the ZnO lattice. In contrast, the C map shows no significant signal in the ZnO layer, in contrast to the wire profiles above (cf. C map in **Figure S22a**). However, after adjusting the contrast and brightness settings of the displayed C map in **Figure S22a**, small C-rich clusters are observed in the ZnO layer (**Figure S22b**). The C-rich spots seem to correlate with the pore positions in the HAADF-STEM image (cf. solid arrows in **Figure S22b**), which show a reduced HAADF-STEM signal due to reduced electron scattering in the pores. This indicates that most of the C is probably present in the pores instead of the ZnO lattice. However, it cannot be ruled out that small C concentrations are also present in the ZnO lattice. The presence of C in the pores may also explain the relatively noisy C line profiles in **Figure S20** and **Figure S21**: The local C concentration along the surface normal (which is horizontally aligned in in **Figure S20** and **Figure S21** and vertically aligned in **Figure S22**) depends on the pore density along the same direction in the analyzed region. This will lead to a relatively high (or low) C signal if many (or only few) pores are averaged along the vertical direction in **Figure S20** and **Figure S21** in the line profiles, ultimately leading to C-signal variations along the line profile. These variations of the C signal are observed in **Figure S20** and **Figure S21**.

Regarding **Figure S22b**, the dashed lines indicate the interfaces between the different layers. The increased C signal near the Pt/C-layer/ZnO interface (see dashed arrow in **Figure S22b**) may be explained by electron-beam broadening into the C-rich Pt/C layer when the electron probe scans near the interface. Since the C signal seems quite noisy and is affected by possible sample contamination in the TEM and X-ray absorption effects, it could not be clarified if and how much C is present in the ZnO film.


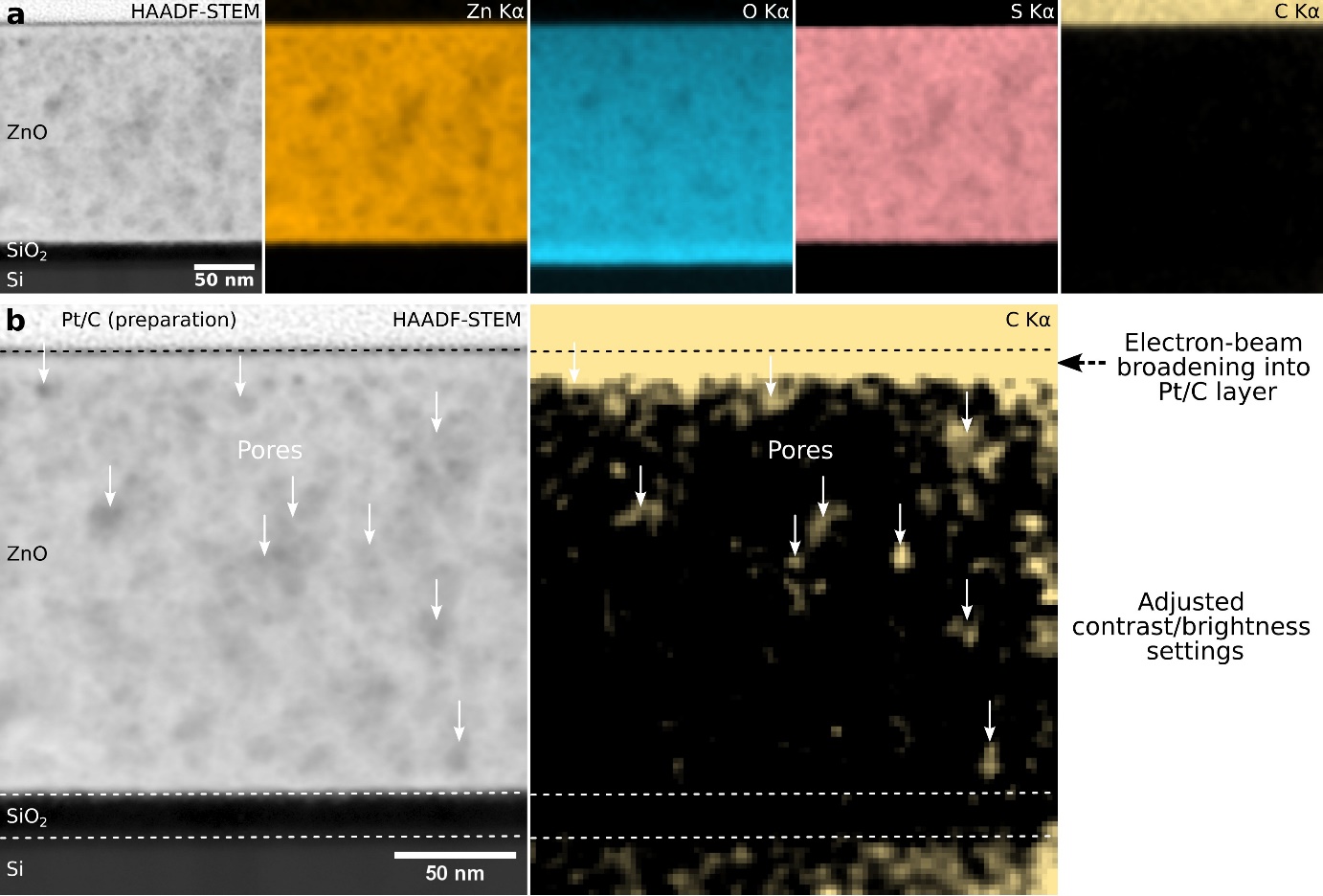


**Figure S22. a** Elemental maps extracted from the PCA-denoised STEM-EDXS dataset. The Zn, O, and S signals are reduced at the pores. C is mostly detected in the Pt/C protection layer.
**b** Magnified HAADF-STEM image with contrast- and brightness-adjusted C map. The latter reveals small C-rich clusters which positions correlate with the pore positions in the HAADF-STEM image (examples are marked with arrows). The high C signal near the Pt/C-layer/ZnO interface at the top likely results from electron-beam broadening into the C-rich Pt/C layer when the electron beam is scanned near the interface.

Regarding the ZnO lattice, S and C can be incorporated into ZnO in small concentrations without significantly affecting the ZnO crystal structure (e.g., for S,^[18–20]^ and for C).^[21,22]^ In principle, ZnS can form a wurtzite structure (space group P6_3_mc, number 186) with larger lattice parameters (*a* = *b* = 0.382 nm, *c* = 0.626 nm) than ZnO (*a* = *b* = 0.325 nm, *c* = 0.521 nm). However, we only observed Bragg reflections and lattice spacings fitting with ZnO in our TEM analyses. Hence, S (and maybe C) may be sparsely distributed in the ZnO host lattice and not form separate crystalline phases. Most C might be present in the pores.

Continuing with the Ar^+^-sputtered SiO_2_ layer, only Si and O signals are observed as expected. Similar to the discussion for ZnO, self-absorption of O Kα X-rays leads to an underestimation of O relative to Si. In addition, beam broadening in the TEM sample leads to signal contribution from the surrounding sample regions (ZnO and Si layers). The nominal concentration is 33.3 at% O and 66.7 at% Si for SiO_2_. Notably, the S and C signals are reduced compared to ZnO, indicating that these elements are only present in ZnO.

The Ar^+^-sputtered Si layer mainly consists of Si (~90 at%) with small concentrations of O (~7 at% to 9 at%) and Ar (~1.5 at% to 2.5 at%). A higher Ar concentration is observed closer to the Si/SiO_2_ interface, and the O concentration behaves vice versa. We suspect that sputtering of the native oxide on the Si target may cause the increased O concentration near the Si/coverslip interface (i.e., during the beginning of the Si deposition), which then gradually reduces as pristine Si is sputtered on the substrate. Finally, the glass coverslip mainly consists of Si and O but also shows other impurity elements such as Ti.

Summarizing the EDXS measurements for the ZnO layer, we mainly observe Zn and O signals with small concentrations of S and C (< 1 at%). Sulfur seems to be mostly incorporated into the ZnO lattice, whereas C is mostly present in the pores. The C and O concentrations are most strongly affected (i.e., underestimated) by absorption effects in the TEM samples. Carbon contamination of the TEM sample is also possible, which can obscure the genuine C signal from the sample. As a result, the measured concentrations should be interpreted carefully.

# HRTEM of the SiO_2_/ZnO Interface

**Figure S23a** shows an aberration-corrected high-resolution TEM (HRTEM) phase-contrast image of the interface region between Si, SiO_2_, and ZnO. The crystalline ZnO layer shows periodic lattice fringes whereas the other two layers have an amorphous structure. The corresponding squared moduli of the Fourier transforms (FT) from the marked regions of the three layers are shown in **Figure S23b**. The interfaces are marked with dashed lines for clarity.

The FT of the ZnO layer shows a distinct set of lattice reflections and reveals a single ZnO<21> orientation starting from the ZnO/SiO_2_ interface. The Ar^+^-sputtered Si and SiO_2_ are amorphous. The FT of the amorphous Si and SiO_2_ layers show a different ring-like signal, which results from differences in mean atomic distances in the two amorphous materials. As a result, a slight difference between the speckle-like contrast for Si and SiO_2_ is also visible in the real-space image. The absence of a polycrystalline seed layer for ZnO indicates that the crystal grows from the preceding crystalline ZnO instead of forming new (randomly oriented) ZnO seeds on the amorphous SiO_2_. In this way, the overall primary ZnO orientation is preserved upon printing.


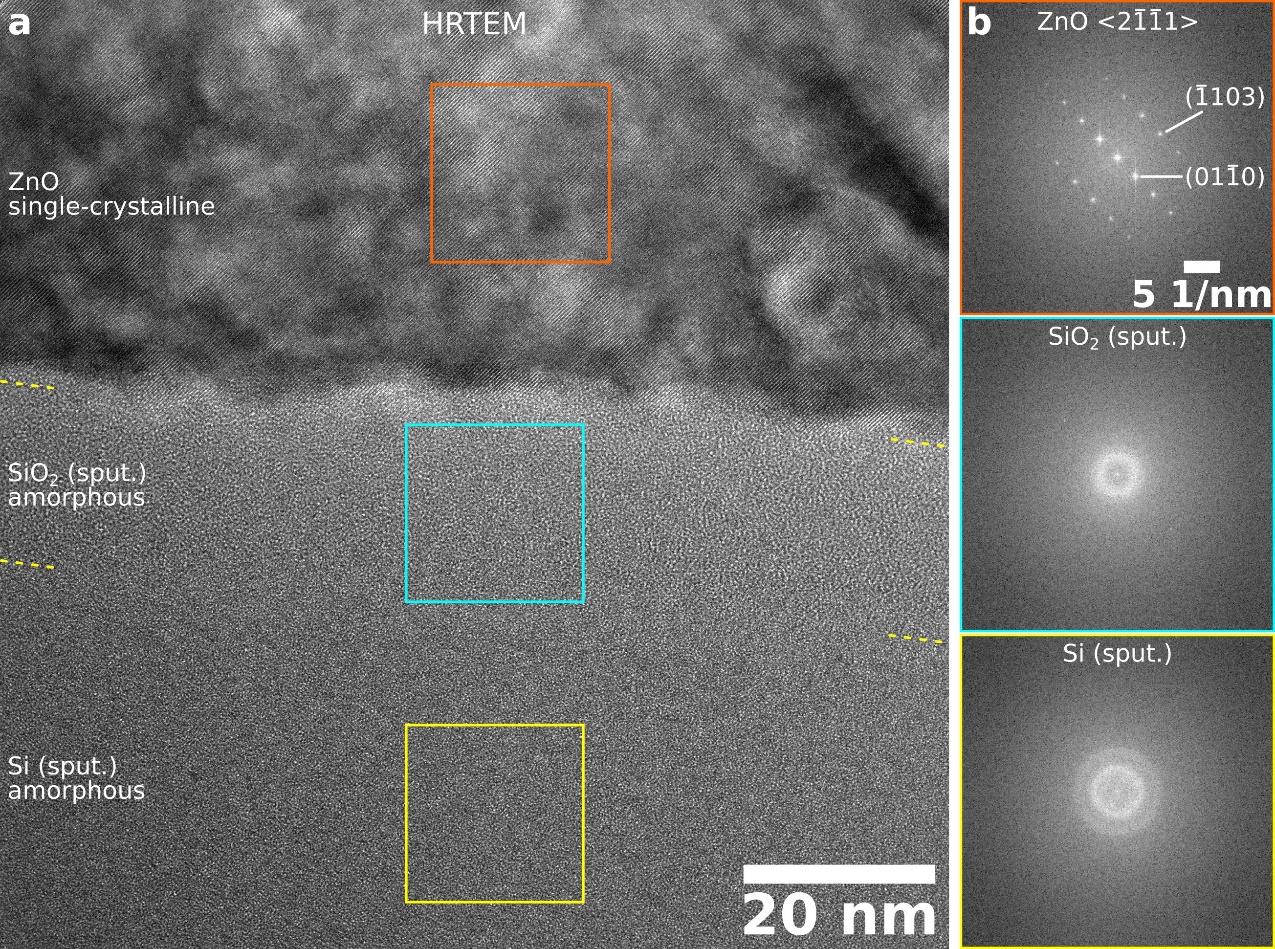


**Figure S23.** Confirmation of single-crystalline ZnO growth on amorphous SiO_2_. **a**High-resolution TEM image of the interfaces between crystalline ZnO and amorphous SiO_2_ and Si. The interfaces are indicated with dashed lines. **b**Corresponding Fourier transforms of the regions marked in a. ZnO shows a distinct set of reflections fitting with the ZnO<20> zone-axis orientation. The other two layers show typical rings for amorphous structures. The different radii result from a difference in mean atomic distances in the two amorphous materials.

A note about the ZnO orientations: The shown HRTEM image was acquired with the sample being aligned to the closest zone axis after loading it in the TEM (here ZnO<21> for this particular ZnO wire). In this way, the electron-beam direction is nearly parallel to the interfaces of the different layers. In contrast, the same TEM sample (from the “EBSD-measured” ZnO wire) was mostly investigated in the low-index ZnO<20> zone axis for the other images shown in this study. This required more tilting of the sample in the TEM and, hence, will lead to a tilted view onto the interfaces.

# Crystallites/Grains observed by HRTEM

For most ZnO wires, one main orientation of ZnO is preserved throughout the printed structure (excluding the starting point). Still, high-resolution HRTEM imaging revealed additional crystallites in some of the HRTEM images as shown in **Figure S24**. The FFT of the HRTEM image in **Figure S24a** shows primarily the spots corresponding the the lattice spacings for ZnO (here in [30] orientation given in 3-index notation, **Figure S24b**). Here, the indexing results of the ReciPro software^[23]^ are shown directly, which uses 3-index notation [*uvw*] for the lattice direction instead of 4-index (Weber) indices [*UVTW*]. The numbered (blue) crosses mark the fitted reflections and the additional unnumbered (green) crosses display the simulated (i.e., expected) spots in the FFT corresponding to the identified crystal phase. Notably, the experimental and simulated positions for the strongest peaks in **Figure S24b** fit well.

Besides the ZnO[30] reflections, additional spots are visible in **Figure S24b**. These correspond to additional lattice planes in **Figure S24a**, that are not readily visible by simple inspection of the image. Fourier-filtering of the extra spots with subsequent Gaussian blurring was used to indicate the spatial location of the nanoparticle causing the extra spots in the FFT (**Figure S24c**). The extra crystallite is located in a (projected) area of about 10 nm × 10 nm.

Next, the positions of the spots in **Figure S24b** were compared with different possible phases, i.e., ZnO (**Figure S24d**), ZnSO_4_ (**Figure S24e**), and ZnCO_3_ (**Figure S24f**) in different crystalline orientations (noted in the top-left corners). These phases are exemplarily shown but other phases (e.g., ZnS) were also checked. In all cases, slight deviations between the simulated and measured spot positions are observed. The simulated higher-order spots of the extra phases seem to align with the main ZnO[30] phase. This indicates that the crystallite grows coherently within the surrounding ZnO matrix. However, no clear results for the exact phase can be obtained from the HRTEM image alone and no chemical analysis was conducted from this specific sample location. The relatively small crystallite may be additionally strained, leading to deviations in lattice parameters (and spot positions) from the simulated values typically measured from bulk crystals. Extra elements such as S and C are present in the ZnO film in small concentrations and may segregate into the extra phases (**Figure S20**-**Figure S22**). However, nm-sized misoriented ZnO grains could also be present in the ZnO film. These crystallites were only detected in a few HRTEM images and the printed ZnO film consists mainly of one single ZnO orientation that changes its orientation along the printing direction.


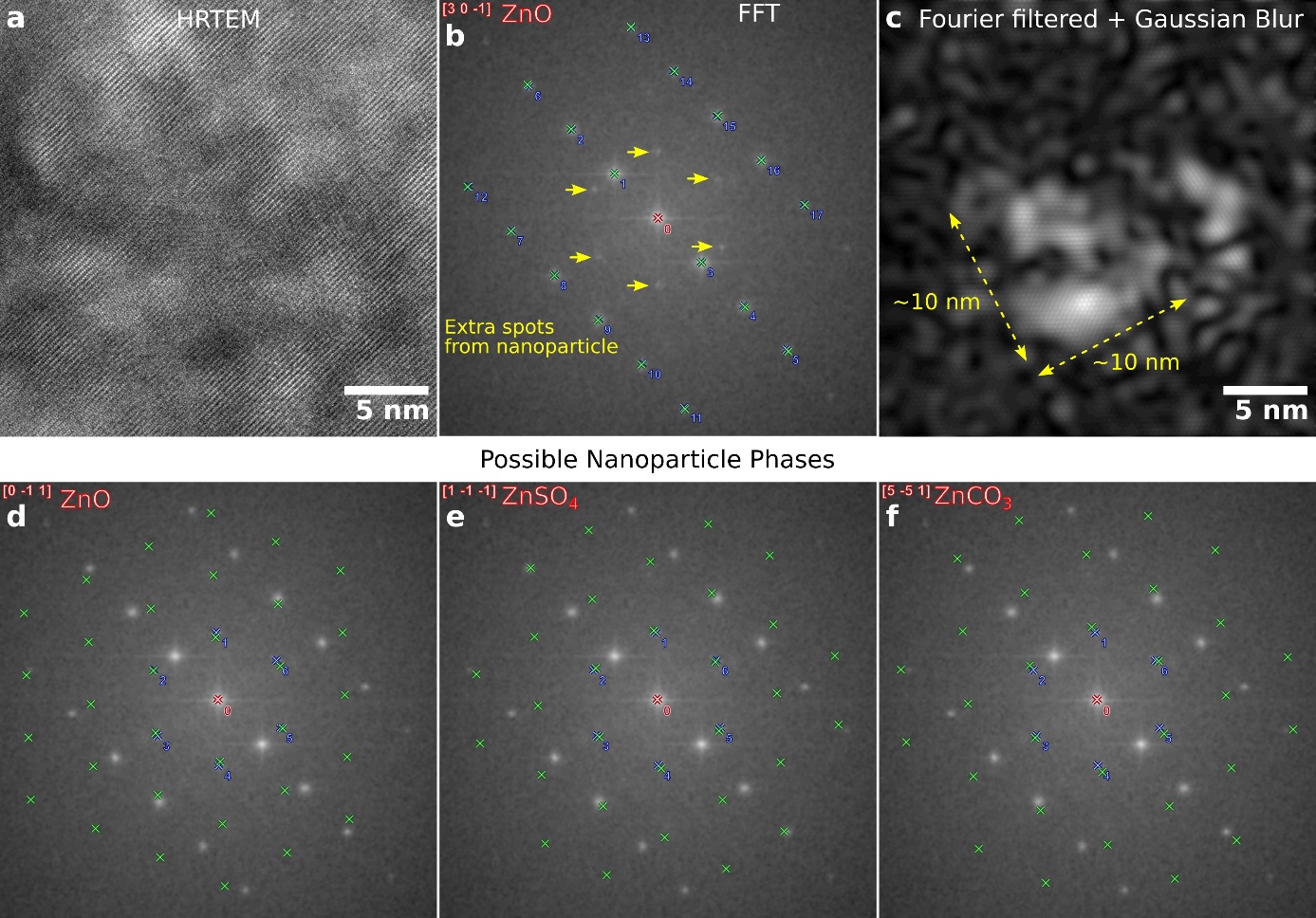


**Figure S24.** HRTEM analysis of nm-sized crystallites found in ZnO. **a**HRTEM image and **b** corresponding FFT with the indexed ZnO[30] main orientation. Numbered (blue) crosses show fitted spots and unnumbered (green) spots show simulated spot positions. A few extra spots are visible, which originate from the embedded crystallite. **c** Fourier-filtered and Gaussian blurred image highlighting roughly the position of the extra spots in the real-space HRTEM image in (a). **d-f**Indexing results for possible crystal phases (ZnO, ZnSO_4_, and ZnCO_3_) for the observed extra spots/lattice planes. All phases fit within reasonable accuracy and the exact phase could not be determined.

# ZnO Lattice Rotation Analysis in the TEM

The observed rotation of ZnO lattice along the printing direction was analyzed by TEM (**Figure S25**). Two TEM samples were extracted and analyzed: one from a wire measured by EBSD (**Figure S25a**) and another one from an as-printed wire (**Figure S25b**). The TEM results from the EBSD-measured wire in **Figure S25a** are compared with the EBSD results.

Overview bright-field TEM images show the relatively large electron-transparent window with a width of about 8 µm (**Figure S25a** and **d**). The relatively large width was chosen to track the lattice rotation over a relatively large area. The TEM sample thickness was kept at around 120 nm to 150 nm for these measurements (and for STEM-EDXS measurements) to minimize any bending of the TEM lamella during thinning. TEM-sample bending would interfere with measuring the genuine lattice rotation of ZnO.


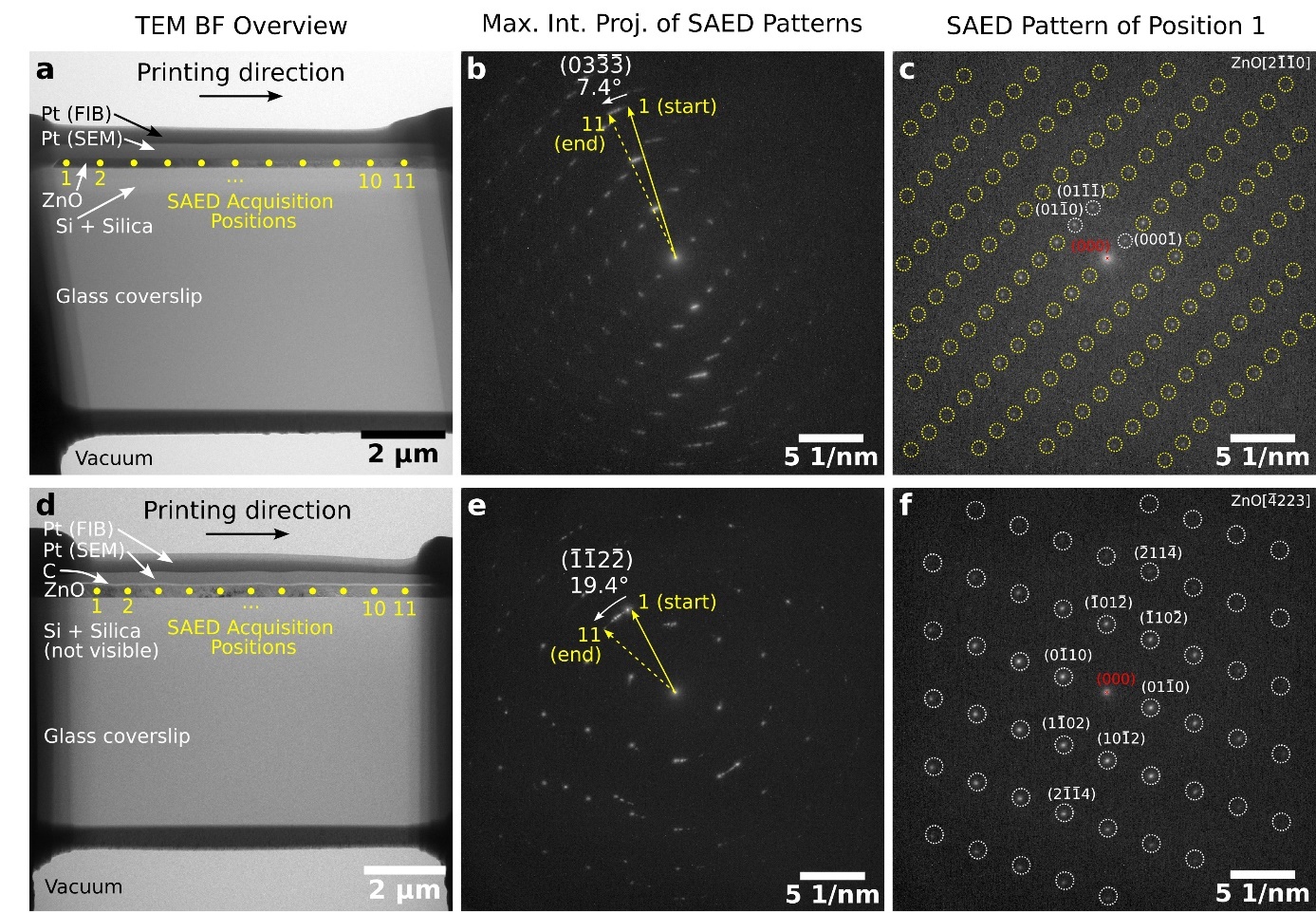


**Figure S25.** Analysis of ZnO lattice rotation in the TEM for the **a-c** EBSD-measured and **d-f** as-printed ZnO wire. **a** and **d** Bright-field TEM overview images with annotations for the different layers and marked SAED acquisitions positions. **b** and **e** Maximum intensity projection of SAED patterns shown all diffraction spots and revealing a counter-clockwise rotation of the ZnO lattice along the printing direction. The SAED-pattern intensities are displayed on a logarithmic scale for better visibility. **c** and **f** Indexed SAED patterns from position 1, where the samples were aligned to the closest zone-axis orientation. The dashed circles mark the simulated spot positions in the patterns for the identified ZnO zone-axis orientations.

Selected-area electron diffraction (SAED) was used to acquire diffraction patterns at 11 different positions in ZnO (cf. circle markers in **Figure S25a** and **d**). With this technique, the orientation of ZnO can be conveniently analyzed over larger sample areas compared to HRTEM imaging at higher magnification. The approach is similar to that of Da *et al.*,^[24]^ even though they used aforementioned HRTEM imaging instead of SAED patterns. The SAED acquisition positions were (i) roughly equidistantly spaced in the electron transparent window along the wire and (ii) also roughly in the center of the ZnO wire (i.e., between the ZnO/SiO_2_ and the ZnO to Pt/C interface). The used SAED aperture with 10 µm diameter selects a ~100 nm diameter circular region of the ZnO layer from which the observed diffraction pattern is formed. The printing direction (i.e., laser-writing direction) is from left to right, starting at SAED pattern position 1. The sample was aligned at this starting position to the closest zone axis (e.g., ZnO[20] in **Figure S25c**). These diffraction patterns at the starting position 1 are indexed in **Figure S25c** and f and fit well with ZnO (**Figure S25c** and **f**). The dashed circles mark the simulated diffraction-spot positions using ReciPro.^[25]^ The orientations for the two wires are different since the initial orientation of the laser-printed ZnO on SiO_2_ is random. Note that kinematically forbidden reflections in ZnO such as (0001), (0003), …, are visible in the SAED patterns (cf. **Figure S25c**) due to multiple-scattering effects in the relatively thick TEM sample.

For each wire, all 11 diffraction patterns were loaded as a stack into Fiji.^[26]^ A pixel binning and a logarithmic intensity scaling were applied for better visibility of the Bragg reflections. Then, a maximum projection was calculated from the stack, resulting in a single image with essentially all Bragg reflections overlaid (**Figure S25b** and **e**). The lattice rotation of ZnO from position 1 to position 11 is observed as a counter-clockwise (CCW) rotation of the diffraction pattern. A distinguishable Bragg reflection with high intensity in all SAED patterns was chosen to measure the lattice rotation (cf. solid and dashed arrows in **Figure S25b** and **e**). This procedure yields a total rotation of ~7.4° for the EBSD-measured wire ((03) reflection) and 19.4° for the as-printed wire ((2) reflection) between positions 1 and 11.

For the EBSD-measured wire and a length of ~7.7 µm between positions 1 and 11, the measured rotation rate of 7.4°/7.7 µm = 0.96° µm^-1^ is significantly smaller than the value of 1.7° µm^-1^ determined by EBSD (cf. **Figure 2** in the main text). The reason for the lower rotation rate in TEM is that only the 2D-projected lattice rotation along the beam direction is tracked, whereas EBSD measures the 3D misorientation (to the reference starting point). Hence, the rotation-axis components not parallel to the electron-beam direction are not measured in our SAED patterns.

The observed CCW rotation of the lattice along the printing direction is an interesting observation since it is *not* in agreement with a proposed model for rotating lattice single crystal growth on amorphous substrates by Koi and De Hosson^[13]^ and Lutijes *et al.*.^[15]^ In their model, the atomic planes that are initially oriented parallel to the surface bend *toward* the substrate. In our case, we would expect a CW rotation of the diffraction spots in the SAED patterns, but a CCW rotation is observed, which is essentially a rotation of the lattice planes *away* from the substrate along the printing direction. The same CCW rotation direction is observed in both wires. However, as seen from the analysis of the lattice planes in **Figure S17**, the re-orientation direction relative to the printing (and growth) direction of the initially surface-parallel lattice planes varies between different wires.

Still, different experimental aspects may explain these differences of our ZnO structure with the literature:

Firstly, the experimental setup and materials are different. In our case, the ZnO crystals are grown from a liquid Zn-containing ink on a solid, amorphous substrate. In contrast, similar growth of rotating crystals with lasers by other groups was performed by laser heating of glass The differences in density between liquid ink and the solid substrate may affect the crystal growth.^[27–29]^

Secondly, a prerequisite of the aforementioned growth model is that the crystallization front must be at the crystal’s surface and not on the crystal/substrate interface.^[13]^ In contrast, here we use the Ar^+^-sputtered light-absorbing Si layer as the heat-inducing layer, which means that the highest temperatures are expected close to the ink/crystal to substrate interface (which is simulated in **Figure S2**). This may affect the rotation direction of the lattice planes. The growth front might be located close to the ZnO/SiO_2_ interface and inclined relative to the printing direction due to interactions with the substrate and volumetric changes in the amorphous-to-crystalline phase transition. The cause for the rotation of the crystal may be the temperature difference created due to the fast cooling after the ZnO is exposed to a steep temperature gradient. This temperature profile allows asymmetric crystal growth (bottom thicker and top thinner) with respect to the surface plane. In addition, the difference in the thermal conductivity of SiO_2_ and ZnO enhances the stress.

Thirdly, our printed ZnO film contains pores which may affect the ZnO RLS crystal’s ability to rotate. In contrast, no pores are mentioned in the previous studies on rotating crystals (to the best of our knowledge). The pores may relax strain in the bending crystal.

Fourthly, the crystal growth depends on the crystal structure. For ZnO in the wurtzite phase, the [0001]-direction is associated with the fastest growth (see main text for a discussion). Other materials with other crystal structure (e.g., Sb_2_S_3_ for the references above) may grow with different preferential orientation, which ultimately affects the lattice rotation.

Overall, a better understanding of the growth process of ZnO for comparison with laser-written rotating crystals in glass or other materials with similar crystal structures requires further experiments.

# Photoluminescence of printed ZnO

To further confirm the non-existence of a photoluminescence signal in the visible range from the printed ZnO, we collected the photoluminescence under a longer exposure (15 seconds) on a larger ZnO wire (**Figure S26**). The peaks from the near-band-edge luminescence and the second harmonic generation are clearly visible as in **Figure 5**. However, there is no luminescence peak observable in the visible range.


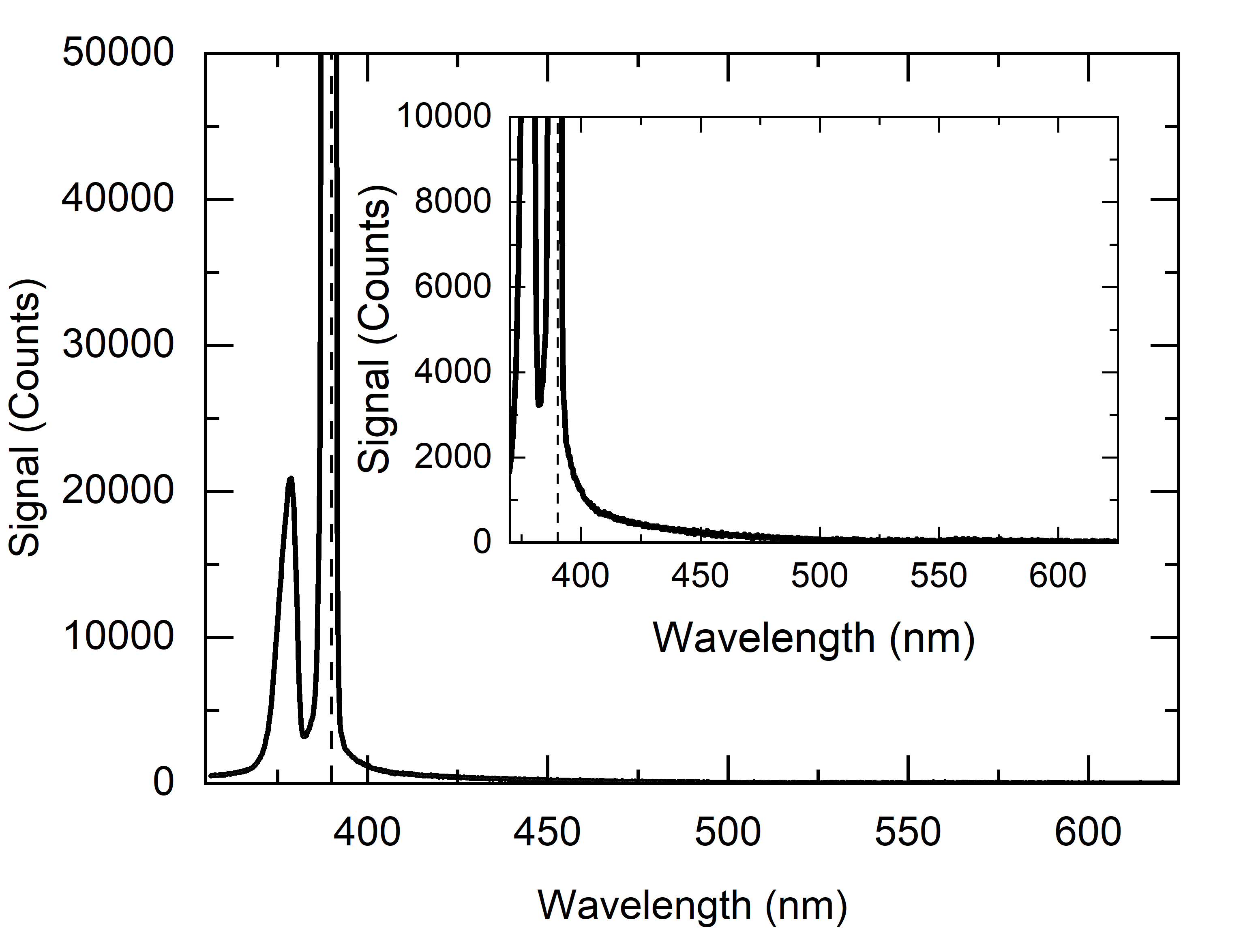


**Figure S26.** Measurements of second harmonic generation and photoluminescence from laser printed ZnO. Recorded spectra from ZnO line printed at 1 µm s^-1^ and 3.08 mW (3.47 MW cm^-2^) printing laser power under 4.6 mW (0.196 TW cm^-2^) exposure from a 780 nm fs laser source. Exposure time of spectrometer CCD was 15 s. Vertical dashed line indicates 390 nm. Inset shows a zoomed-in view of the collected visible range.

# TEM-sample preparation by FIB milling

**Figure S27a** and **b** show top-view SE-SEM images of the two ZnO wires chosen for TEM analyses (length ~10.5 µm, width about 500 nm to 580 nm measured from the shown SEM images). Preceding EBSD measurements were performed on the EBSD-measured wire whereas the as-printed wire is in its genuine state after printing. At the beginning, two wires with a low nominal FIB current of 9 pA were milled at the sides of the wires to easier locate the ZnO film during final thinning to electron transparency (schematically shown with dashed lines in **Figure S27b**). These lines are visible in the top-view SE-SEM of the TEM sample after milling the large trenches before the actual TEM-sample lift out. Note that the deposited protective Pt/C layers are wider than the ZnO wires along the vertical direction so the markers are required to keep track of the ZnO location. Note that because the printed ZnO film does not have a uniform thickness profile after printing, the ZnO layer thickness observed by TEM varies depending on the exact thinning position of the TEM sample during thinning. After the in-situ lift-out with a micromanipulator, the TEM samples are fixed on two sides on a FIB-milled trench (**Figure S27d**, SE-FIB image, tilted view). Two-side fixation is used to prevent any bending of the samples. In addition, the TEM samples were kept at a final thickness of around 120 nm to 150 nm for SAED (and EDXS) measurements to further minimize any bending for the relatively wide electron-transparent TEM windows (about 8 µm width). In all steps, care was taken to track the printing direction of the wires. For example, markers were FIB-milled into the TEM grid to mark the starting side of the printing process.


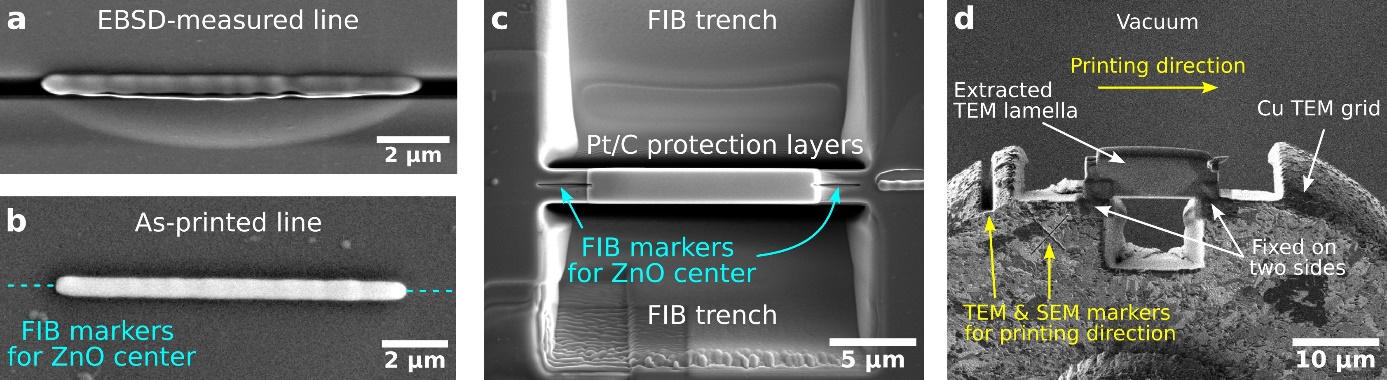


**Figure S27.** Top-view SE-SEM images of **a** EBSD-measured and **b** as-printed ZnO wires just prior to TEM-sample preparation. FIB-milled markers are used to keep track of the ZnO wire centers. **c** Top-view SE-SEM of the TEM sample before the lift out from the bulk sample. The FIB markers are visible as lines on the side of the Pt/C protection layers. **d** Tilted-view SE-FIB image of the fixed TEM sample prior to thinning. The sample is fixed on two sides to increase stability. FIB-milled markers are used to mark the starting side of the printing process.

Final thinning steps were performed at low Ga^+^ energies of 3 keV to minimize Ga implantation and surface amorphization. Notably, the pores observed in cross-section STEM images (**Figure 4** in the main text) could be sample damage caused by Ga^+^-ion bombardment during FIB preparation. However, since (i) a relatively low FIB Ga^+^-ion energy of 3 keV was used for final polishing and (ii) the pores are also confirmed by SEM imaging (**Figure S8**), we conclude that the dark areas in HAADF-STEM images are the result of pores in the ZnO film.

In addition, the local HAADF-STEM image intensity in a pore area depends on its size and the number of pores intersected by the electron beam along the TEM-sample thickness, resulting in a lower image intensity for (i) either larger-sized pores or (ii) a higher number of intersecting pores. As a result, the actual pore-size distribution cannot be easily determined by TEM.

# References

[1] J. Zhou, Y. Che, K. Wu, J. Shen, C. He, *J. Chem. Eng. Data* **2013**, *58*, 663–670.

[2] J.-P. E. Grolier, G. Roux-Desgranges, M. Berkane, E. Jiménez, E. Wilhelm, *The Journal of Chemical Thermodynamics* **1993**, *25*, 41–50.

[3] W. S. MacGregor, *Annals of the New York Academy of Sciences* **1967**, *141*, 3–12.

[4] “MatWeb,” can be found under https://www.matweb.com, **n.d.**

[5] E. Yamasue, M. Susa, H. Fukuyama, K. Nagata, *Journal of Crystal Growth* **2002**, *234*, 121–131.

[6] J. L. Braun, C. H. Baker, A. Giri, M. Elahi, K. Artyushkova, T. E. Beechem, P. M. Norris, Z. C. Leseman, J. T. Gaskins, P. E. Hopkins, *Phys Rev B* **2016**, *93*, 1–5.

[7] C. Schinke, P. Christian Peest, J. Schmidt, R. Brendel, K. Bothe, M. R. Vogt, I. Kröger, S. Winter, A. Schirmacher, S. Lim, H. T. Nguyen, D. Macdonald, *AIP Advances* **2015**, *5*, DOI 10.1063/1.4923379.

[8] M. W. Chase, in *J. of Physical and Chemical Reffernce Data, Monograph 9*, **1998**, pp. 1529–1564.

[9] J. H. Hubbell, S. M. Seltzer, “X-Ray Mass Attenuation Coefficients,” **1996**.

[10] D. Savytskii, E. Musterman, V. Dierolf, H. Jain, *Cryst Growth Des* **2019**, *19*, 6324–6330.

[11] S. Hong, J. Yeo, W. Manorotkul, H. W. Kang, J. Lee, S. Han, Y. Rho, Y. D. Suh, H. J. Sung, S. H. Ko, *Nanoscale* **2013**, *5*, 3698–3703.

[12] V. Y. Kolosov, A. R. Thölén, *Acta Mater* **2000**, *48*, 1829–1840.

[13] B. J. Kooi, J. T. M. De Hosson, *J Appl Phys* **2004**, *95*, 4714–4721.

[14] H. Bachmann, F., Hielscher, R., Schaeben, *Solid State Phenomena* **2010**, *160*, 63–68.

[15] N. R. Lutjes, S. Zhou, J. Antoja-Lleonart, B. Noheda, V. Ocelík, *Sci Rep* **2021**, *11*, DOI 10.1038/s41598-021-94147-y.

[16] S. H. Yoon, D.-J. Kim, *Journal of Crystal Growth* **2007**, *303*, 568–573.

[17] J. Wang, R. Chen, L. Xiang, S. Komarneni, *Ceramics International* **2018**, *44*, 7357–7377.

[18] Y.-Z. Yoo, Z.-W. Jin, T. Chikyow, T. Fukumura, M. Kawasaki, H. Koinuma, *Appl Phys Lett* **2002**, *81*, 3798–3800.

[19] F. Fabbri, L. Nasi, P. Fedeli, P. Ferro, G. Salviati, R. Mosca, A. Calzolari, A. Catellani, *Sci Rep* **2016**, *6*, 27948.

[20] D. F. Wang, L. Liao, J. C. Li, Q. Fu, M. Z. Peng, J. M. Zhou, *Chinese Physics Letters* **2005**, *22*, 2084–2087.

[21] D. T. Ngo, L. T. Cuong, N. H. Cuong, C. T. Son, P. T. Huy, N. D. Dung, *Advanced Functional Materials* **2018**, *28*, 1–6.

[22] X. Zhang, J. Qin, R. Hao, L. Wang, X. Shen, R. Yu, S. Limpanart, M. Ma, R. Liu, *Journal of Physical Chemistry C* **2015**, *119*, 20544–20554.

[23] Y. Seto, M. Ohtsuka, *J Appl Crystallogr* **2022**, *55*, 397–410.

[24] B. Da, L. Cheng, X. Liu, K. Shigeto, K. Tsukagoshi, T. Nabatame, Z. Ding, Y. Sun, J. Hu, J. Liu, D. Tang, H. Zhang, Z. Gao, H. Guo, H. Yoshikawa, S. Tanuma, *Science and Technology of Advanced Materials: Methods* **2023**, *3*, DOI 10.1080/27660400.2023.2230870.

[25] Y. Seto, M. Ohtsuka, *Journal of Applied Crystallography* **2022**, *55*, 397–410.

[26] J. Schindelin, I. Arganda-Carreras, E. Frise, V. Kaynig, M. Longair, T. Pietzsch, S. Preibisch, C. Rueden, S. Saalfeld, B. Schmid, J.-Y. Tinevez, D. J. White, V. Hartenstein, K. Eliceiri, P. Tomancak, A. Cardona, *Nat Methods* **2012**, *9*, 676–682.

[27] D. Savytskii, C. Au-Yeung, V. Dierolf, N. Tamura, H. Jain, *Crystal Growth and Design* **2017**, *17*, 1735–1746.

[28] D. Savytskii, H. Jain, N. Tamura, V. Dierolf, *Sci Rep* **2016**, *6*, DOI 10.1038/srep36449.

[29] E. J. Musterman, D. Savytskii, V. Dierolf, H. Jain, *Scr Mater* **2021**, *193*, 22–26.
